# Coumarin-Based Compounds as Inhibitors of Tyrosinase/Tyrosine Hydroxylase: Synthesis, Kinetic Studies, and In Silico Approaches

**DOI:** 10.3390/ijms24065216

**Published:** 2023-03-09

**Authors:** Jéssica Alves Nunes, Rodrigo Santos Aquino de Araújo, Fabrícia Nunes da Silva, Joanna Cytarska, Krzysztof Z. Łączkowski, Sílvia Helena Cardoso, Francisco Jaime Bezerra Mendonça-Júnior, Edeildo Ferreira da Silva-Júnior

**Affiliations:** 1 Biological and Molecular Chemistry Research Group, Institute of Chemistry and Biotechnology, Federal University of Alagoas, AC Simões Campus, Lourival Melo Mota Avenue, s/n, Maceió 57072-970, Alagoas, Braziledeildo.junior@iqb.ufal.br (E.F.d.S.-J.); 2 Laboratory of Synthesis and Drug Delivery, Department of Biological Sciences, State University of Paraíba, João Pessoa 58429-500, Paraíba, Brazil; 3 Laboratory of Organic and Medicinal Synthesis, Federal University of Alagoas, Campus Arapiraca, Manoel Severino Barbosa Avenue, Arapiraca 57309-005, Alagoas, Brazil; 4 Department of Chemical Technology and Pharmaceuticals, Faculty of Pharmacy, Collegium Medicum, Nicolaus Copernicus University, Jurasza 2, 85-089 Bydgoszcz, Poland

**Keywords:** tyrosinase, melanoma, skin cancer, copper, inhibitors, dynamics, docking

## Abstract

Cancer represents the main cause of morbidity and mortality worldwide, constituting a serious health problem. In this context, melanoma represents the most aggressive and fatal type of skin cancer, with death rates increasing every year. Scientific efforts have been addressed to the development of inhibitors targeting the tyrosinase enzyme as potential anti-melanoma agents due to the importance of this enzyme in melanogenesis biosynthesis. Coumarin-based compounds have shown potential activity as anti-melanoma agents and tyrosinase inhibitors. In this study, coumarin-based derivatives were designed, synthesized, and experimentally evaluated upon tyrosinase. Compound **FN-19**, a coumarin–thiosemicarbazone analog, exhibited potent anti-tyrosinase activity, with an IC_50_ value of 42.16 ± 5.16 µM, being more active than ascorbic acid and kojic acid, both reference inhibitors. The kinetic study showed that **FN-19** acts as a mixed inhibitor. Still, for this compound, molecular dynamics (MD) simulations were performed to determine the stability of the complex with tyrosinase, generating RMSD, RMSF, and interaction plots. Additionally, docking studies were performed to elucidate the binding pose at the tyrosinase, suggesting that the hydroxyl group of coumarin derivative performs coordinate bonds (bidentate) with the copper(II) ions at distances ranging from 2.09 to 2.61 Å. Then, MM/PBSA calculations revealed that van der Waals interactions are the most relevant intermolecular forces for complex stabilization. Furthermore, it was observed that **FN-19** has a binding energy (Δ*E_MM_*) value similar to tropolone, a tyrosinase inhibitor. Therefore, the data obtained in this study will be useful for designing and developing novel coumarin-based analogs targeting the tyrosinase enzyme.

## 1. Introduction

Cancer represents one of the main causes of morbidity and mortality worldwide, accounting for approximately 10 million deaths only in 2020 [1]. According to estimates by Global Cancer Data (GLOBOCAN), about 19.3 million new cases of cancer occurred in the same year. Still, it is estimated a total of 28.4 million new cases will be diagnosed by 2040 [2]. Cancer is related to a group of diseases characterized by the uncontrolled growth of abnormal cells, which have the potential to spread to other organs [3]. Among more than 100 different types of existing cancers, skin cancer stands out, which is broadly divided into non-melanoma skin cancer (NMSC) and melanoma [4,5]. Melanoma is the third most common type of skin cancer and also the most aggressive/fatal form of the disease [6]. In the United States, 88,059 melanoma cases were registered, leading to 8092 deaths only in 2019. For 2022, there was an estimated number of 99,780 new cases (57,180 men and 42,600 women) and 7650 deaths only in the US [7]. The risk of melanoma may be associated with both endogenous and exogenous factors, such as a family history of melanoma, the existence of melanocytic or dysplastic nevi, and excessive exposure to ultraviolet (UV) rays [8,9,10]. Most of the genetic alterations involved in the development of melanoma correspond to somatic mutations acquired throughout life [11]. Melanoma originated from melanocytes, which are pigmented cells responsible for producing melanin, a pigment with a photoprotective effect [12,13].

The biosynthesis of this pigment involves a cascade of enzymatic reactions in which the role of tyrosinase enzymes (also known as tyrosine hydroxylases), tyrosinase-related protein 1 (TYRP-1) and 2 (TYRP-2), is crucial [14]. Excess melanin, resulting from abnormal overexpression of tyrosinase, is associated with hyperpigmentation and melanoma diseases [15]. Tyrosinase is a metalloenzyme that has two copper(II) ions at its active site, which perform coordination bonds with six histidine residues [16,17,18]. It catalyzes the transformation of L-tyrosine to *O*-dopaquinone via two sequential reactions: the hydroxylation, via monophenolase action, from monophenol L-tyrosine to *O*-diphenol L-DOPA, followed by its oxidation to *O*-dopaquinone, via diphenolase action [19,20]. This *O*-quinone is one of the precursors of melanin and, therefore, an overexpression of tyrosinase induces an overproduction of the pigment, which is associated with hyperpigmentation and melanoma diseases [15]. The existence of two copper(II) ions at the active site of tyrosinase, which can exist in different oxidation states, allows them to mediate the redox mechanisms mentioned above [21] and ponders the mode of binding of the kojic acid inhibitor [22]. This mechanism is widely known and used, due to its chelating properties, in the food industry to prevent oxidative browning and in many cosmetics to lighten skin, even being utilized to treat melasma [23]. Considering the central role of tyrosinase in melanogenesis, and its relationships with the development of melanoma, efforts have been directed toward the development of drugs that inhibit its activities, such as anti-melanoma agents [19].

**Figure 1:**
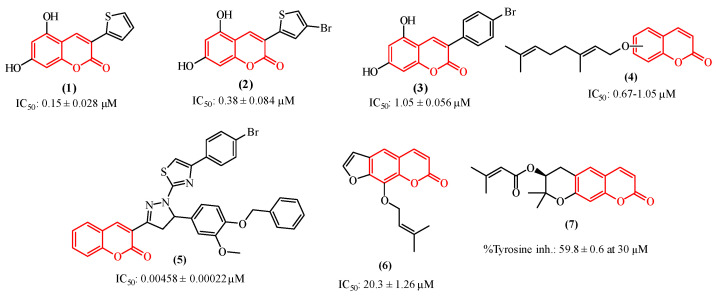
Coumarin-based compounds with activity against the tyrosinase enzyme.

Among the tyrosinase inhibitors, several authors have described the role of secondary plant metabolites, especially phenolic derivatives [24,25,26], including coumarins or 2*H*-1-benzopyran-2-one, which are chemically characterized by the presence of a benzene ring fused to an *α*-pyrone [27]. This class of secondary metabolites arouses great interest in the academic community due to their vast pharmacological applications [28,29,30,31], such as their antitumor activities toward various cancer lineages [32,33,34,35], including human colon cancer (HCT-116) and human adenocarcinoma (HeLa) [36], and also their properties as anti-melasma agents and tyrosinase inhibitors [37,38]. As some examples (Figure 1), we can highlight the studies performed by Pintus et al. and Matos et al., who synthesized a series of heteroarylcoumarins and evaluated their inhibitory activities on mushroom tyrosinase, identifying compounds **1** and **2** as the most promising tyrosinase inhibitors [39,40,41]. Additionally, compounds **2** and **3** were able to inhibit cellular tyrosinase activity and melanin production in B16F10 cells. In addition, Roh observed that among a group of synthetic coumarins, the geranyloxycoumarin derivatives (**4**) displayed tyrosinase inhibition at the lowest concentrations, ranging from 0.67 to 1.05 µM [42]. In a study involving a series of thiazole-containing coumarinyl–pyrazolinyl analogs as potential tyrosinase inhibitors, Saeed et al. found that compound **5** is a promising tyrosinase inhibitor [43]. Pynam & Dharmesh found that marmelosin **6**, a furanocoumarin from the Bael fruit (*Aegle marmelos* L.) displays excellent anti-tyrosinase activity [44]. In addition, Choi et al. explored decursin **7**, a natural component identified in *Angelica gigas* Nakai, and verified that it reduces the production of melanin as well as decreases the intracellular activity of tyrosinase [45].

**Figure 2:**
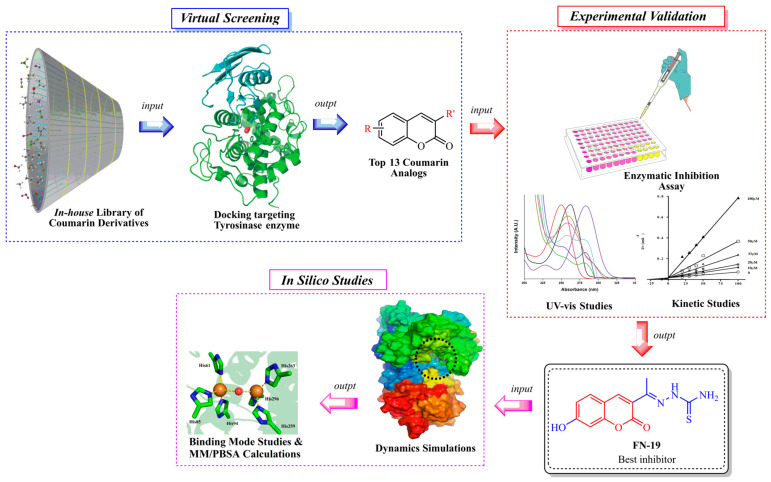
Workflow used in this study to identify tyrosinase inhibitors.

Notwithstanding all these promising coumarin derivatives found in the literature, herein, we performed an investigation focused on an *in-house* library of coumarins, aiming to identify potential inhibitors of the tyrosinase enzyme, which could be promising drug candidates against melanoma. Initially, over 100 coumarin analogs (see Supplementary Information) were virtually investigated for their binding affinity toward tyrosinase. Posteriorly, the most promising coumarins were selected and synthesized for further investigation. Then, enzymatic inhibition assays upon tyrosinase were performed, and the binding modes of the compounds were determined with kinetic studies. Then, in silico studies were carried out to determine the binding modes and complex stability for the best inhibitor complexed with tyrosinase. The workflow used in this study is shown in Figure 2.

## 2. Results and Discussions

### 2.1. Chemistry

**Scheme 1:**
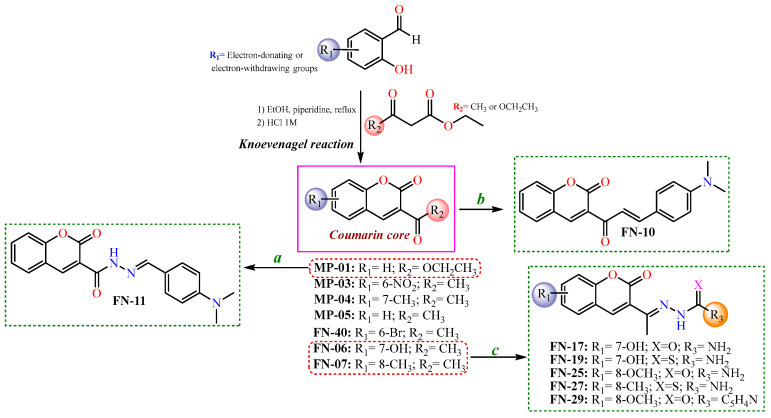
Synthetic routes to obtain coumarin-based compounds. Reaction conditions: (**a**) hydrazine solution, corresponding aldehyde, in ethanol refluxed; (**b**) N,N-dimethylamino-p-benzaldehyde, piperidine catalytic amounts, in ethanol; and (**c**) semicarbazide or thiosemicarbazide, aqueous-ethanolic solution (1:1), acetic acid catalytic amounts.

After the initial virtual screening step, all structures were ranked based on their binding affinities, from the lowest (≤25) to the highest (≥65). Thus, the most promising coumarin candidates (binding affinity ≥55) were selected and synthesized for further experimental investigations. In this context, the synthesis of these coumarin nuclei was performed with the Knoevenagel reactions [46], resulting in coumarins-3-acetyl- (**MP-01**) or 3-ester-substituted (**MP-3-5**, **FN-06**, **07**, and **40**) derivatives. Subsequently, to obtain the coumarins containing semicarbazide, thiosemicarbazide, or isoniazid moieties, nucleophilic addition reactions were performed, involving initially 3-acetyl-coumarins and hydrazides. Regarding the synthesis of coumarin–chalcone (**FN-10**), a different synthetic route was adopted using an aldol condensation reaction. Finally, the coumarin–*N*-acylhydrazone (**FN-11**) was obtained from a coumarin ester (**MP-01**) refluxed with a hydrazine solution, followed by the Knoevenagel reaction, refluxing with the corresponding aldehyde in ethanol [47]. Aiming to develop more efficient tyrosinase inhibitors, in addition to those acetyl coumarin derivates with electron-withdrawing and electron-donating groups in their aromatic ring, a series of hybrid scaffolds were also developed having either a thiosemicarbazone, a semicarbazone, an *N*-acylhydrazone, an isonicotinoylhydrazone, or a chalcone moiety attached to the coumarin ring. All strategies presented to synthesize the coumarin-based compounds in this study are shown in Scheme 1.

All synthesized compounds were chemically characterized using spectroscopic and spectrometric techniques. Initially, their purity (%) and retention time (R_T_) were determined using high-performance liquid chromatography with diode-array detection (HPLC-DAD), in which all final compounds exhibited at least 95% purity and R_T_ values ranging from 2.77 to 3.42 min (see Supplementary Information). Concerning their Attenuated Total Reflectance Fourier-Transformed Infrared (ATR-FTIR) spectra, vibrational stretching (*υ*) was observed ranging from 1761 to 1595 cm^−1^ for the C=O group of ketones, esters, and lactone (coumarin ring). For **FN-11**, **17**, **19**, **25**, **27**, and **29**, their υ(N−H) were verified to vary from 3514 to 3311 cm^−1^. Regarding their hydrogen Nuclear Magnetic Resonance (^1^H NMR) spectra, singlets (*s*) ranging from 8.2 to 8.5 ppm were observed for the coumarin hydrogen at position 4. For **MP-03**, **MP-04**, **MP-05**, **FN-06**, and **FN-07**, their hydrogens from their methyl-ketone group at position 3 were verified to vary from 2.5 to 2.71 ppm. Moreover, broad singlets (*br s*) ranging from 9.43 to 9.52 ppm were identified for NH hydrazone moiety for **FN-17** and **25**. Otherwise, these signals were observed to range from 10.3 to 10.4 ppm for **FN-19** and **27**, whereas it was displayed at 11 ppm for **FN-29**. This could be associated with the presence of the pyridine ring, which affects the electronic resonance of the molecule. **FN-11** exhibits a broad singlet (*br s*) for the NH hydrazone group at 11.96 ppm. Regarding the carbon 13 NMR (^13^C NMR), it was verified that **MP-03**, **04**, and **05**, and also **FN-06** and **FN-07**, exhibit their ketone carbonyl signal ranging from 194.4 to 195.7 ppm. For **FN-19** and **27**, a signal ranging from 179.0 to 179.2 ppm is referent to their thiocarbonyl group. Additionally, the imine group was observed to vary from 146.2 to 157.6 ppm for the thiosemicarbazones (**FN-19** and **FN-27**) and semicarbazones (**FN-17** and **FN-25**). Finally, Gas Chromatography-Mass Spectrometry (CG/MS) analyses revealed that these compounds present retention time (R_T_) varying from 12.0 to 18.3 min, and their fragmentation profiles (*M/Z* ratio) are compatible with the compounds synthesized in this study.

### 2.2. Mushroom Inhibition Assays

All compounds were tested for their inhibitory effects on mushroom tyrosinase using L-DOPA as substrate. The results are summarized in Table 1. In general, the results were compared with standard tyrosinase inhibitors, such as kojic acid and ascorbic acid. All tested compounds showed a higher effect of tyrosinase inhibition than that of ascorbic acid (IC_50_: 386.5 ± 11.95 µM). In contrast, only **FN-19** was more active than kojic acid (IC_50_: 72.27 ± 3.14 µM), whereas **FN-10** can be considered an equivalent inhibitor.

As aforementioned, the best inhibition effect was demonstrated by compound **FN-19**, with an IC_50_ value of 42.16 ± 5.16 µM. Its inhibitory activity is two-fold higher than that of kojic acid (IC_50_: 72.27 ± 3.14 µM) and nine-fold higher than that of ascorbic acid (IC_50_: 386.5 ± 11.95 µM). Activity comparable to kojic acid was shown in screening **FN-10** (IC_50_: 72.40 ± 6.53 µM). In addition, the compounds **FN-06** and **FN-27** showed great inhibitory activity when compared to the other compounds evaluated with IC_50_ values of 86.72 ± 7.29 and 89.75 ± 4.14 µM, respectively. Our data also indicate that compound **FN-17** shows slightly lower inhibitory activity, with an IC_50_ value of 112.79 µM. Activity about two-fold lower than that of kojic acid was observed for compounds **FN-11** and **MP-05**, with IC_50_ values of 158.65 ± 14.63 and 155.68 ± 17.43 µM, respectively. The other tested compounds are characterized by tyrosinase inhibition with IC_50_ values ranging from 192.35 to 317.82 µM, with the smallest tyrosinase inhibition effect observed for compound **FN-40**. Based on these biological results of the inhibition of the tyrosinase enzyme, it was possible to perform the structure–activity relationship (SAR) analysis. Starting from the non-substituted ketone–coumarin nucleus (**MP-05**), which has low activity in the anti-tyrosinase assay (IC_50_: 155.68 ± 17.43 µM). Thus, when a bromine atom is inserted at position 6 of **MP-05**, a compound even less active is obtained (**FN-40**). Posteriorly, when this group is replaced with a nitro group, a poorly active compound is generated (**MP-03**). This activity is slightly increased when a methyl substituent is inserted at position 7 of **MP-05**, leading to compound **MP-04** (IC_50_: 222.03 ± 30.80 µM). When this methyl group is replaced with a hydroxyl group, the activity is drastically improved, obtaining the compound **FN-06** (IC_50_: 86.72 ± 7.29 µM). Subsequently, an 8-methoxyl-containing coumarin analog (**FN-07**) presented reduced activity. Still, **MP-05** generated the compound **FN-10**, which has phenyl dimethylamine attached to a vinyl ketone moiety, an active compound (IC_50_: 72.40 ± 6.53 µM). However, when this vinyl ketone moiety is replaced with an acylhydrazone moiety, a compound with reduced activity is obtained (**FN-11**). By structurally modifying the coumarin **FN-06** and **FN-07** analogs, it was possible to obtain semicarbazones (**FN-17** and **FN-25**), thiosemicarbazones (**FN-19** and **FN-27**), and pyridylacylhydrazone (**FN-29**). In this context, when a hydroxyl group is inserted at position 7 of the **FN-25** analog, a compound with improved activity is obtained (**FN-17**), having an IC_50_ value of 112.79 ± 4.64 µM. In contrast, when a carbonyl group is replaced with a thiocarbonyl group, generating the aforementioned thiosemicarbazones, a meaningful increase is observed in the activity of these analogs. Still, with the insertion of a hydroxyl group at position 7 of compound **FN-27**, the best inhibitor is obtained (**FN-19**), being more active (IC_50_: 42.16 ± 5.16 µM) than the positive controls in this assay. Posteriorly, when a pyridylacylhydrazone moiety replaces the thiosemicarbazone one, having also an 8-methoxyl group, a not soluble compound (**FN-29**) is obtained. Furthermore, it could be stated that the thiosemicarbazone moiety is responsible for improving the activity of the coumarin nucleus. In this context, thiosemicarbazones have been reported as promising tyrosinase inhibitors. These compounds are known to be active as chelating agents toward copper ions, reducing the enzymatic activity of tyrosinase [48,49,50]. Still, some studies have reported that the hydrazone moiety is a pharmacophore group in compounds targeting tyrosinase [51,52]. Concerning the second-best inhibitor, **FN-10**, a study reported the use of chalcone moiety in tyrosinase inhibitors [53]. Finally, it could be observed that hydroxyl-containing coumarins (at position 7) may have an essential role in tyrosinase inhibition, which is corroborated by different studies in the literature [19,39,54].

### 2.3. Kinetic Analysis of the Inhibition of Tyrosinase

**Figure 3:**
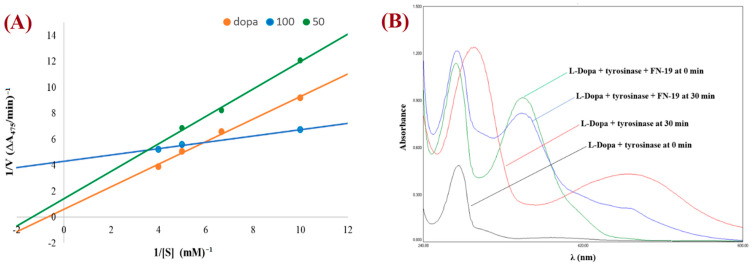
Lineweaver–Burk plot for FN-19 (at 100 mM (blue) and 50 mM (green)) obtained with the Michaelis–Menten equation (**A**) and UV-vis spectra for L-DOPA by tyrosinase in the presence and absence of FN-19 inhibitor (**B**).

The mechanism for tyrosinase inhibition of the tested compounds was determined from Lineweaver–Burk double reciprocal plots. The plot provides a useful graphical method for analysis of the Michaelis–Menten equations used to determine the *K_m_* values for possible competitive, uncompetitive, non-competitive, and mixed mechanisms of inhibition. The graph obtained for the most promising compound, **FN-19**, is shown in Figure 3A. All Lineweaver–Burk plots can be found in the Supplementary Information. As can be easily noticed, the majority of tested compounds are characterized by the mixed mode of inhibition (Table 2). It is a type of enzyme inhibition in which the inhibitor may bind to the enzyme whether or not the enzyme has already bound to the substrate.

The derivative **MP-04** showed a non-competitive mechanism of action, which indicates that the inhibitor and substrate may bind to the enzyme at the same time to form the enzyme–substrate–inhibitor complex, which prevents the formation of the product. Compound **MP-03** shows an uncompetitive mechanism of inhibition, in which an enzyme inhibitor binds only to the complex formed between the enzyme and the substrate. The remaining compound **FN-40** is a competitive inhibitor, which binds to the enzyme at its active site, preventing the binding of the substrate to the enzyme. The compound **FN-29** is characterized by the lowest value of the affinity constant (*K_m_* value of 0.03), exhibiting the strongest inhibition of tyrosinase. To observe the effect of the oxidation of L-DOPA by tyrosinase in the absence and presence of the tested compounds, the UV-Vis spectra were determined. Thus, the UV-vis spectra for the compound with the best inhibition effect, **FN-19**, are presented in Figure 3B. During L-DOPA oxidation by tyrosinase, a characteristic signal at 475 nm is observed in the UV-Vis spectrum, corresponding to the formation of dopachrome. After the addition of inhibitor **FN-19** after 30 min, we observed a significant reduction in the signal intensity at 475 nm in comparison with the absorbance of the mixture of L-DOPA with tyrosinase.

### 2.4. Molecular Dynamics (MD) Simulations and Docking

**Figure 4:**
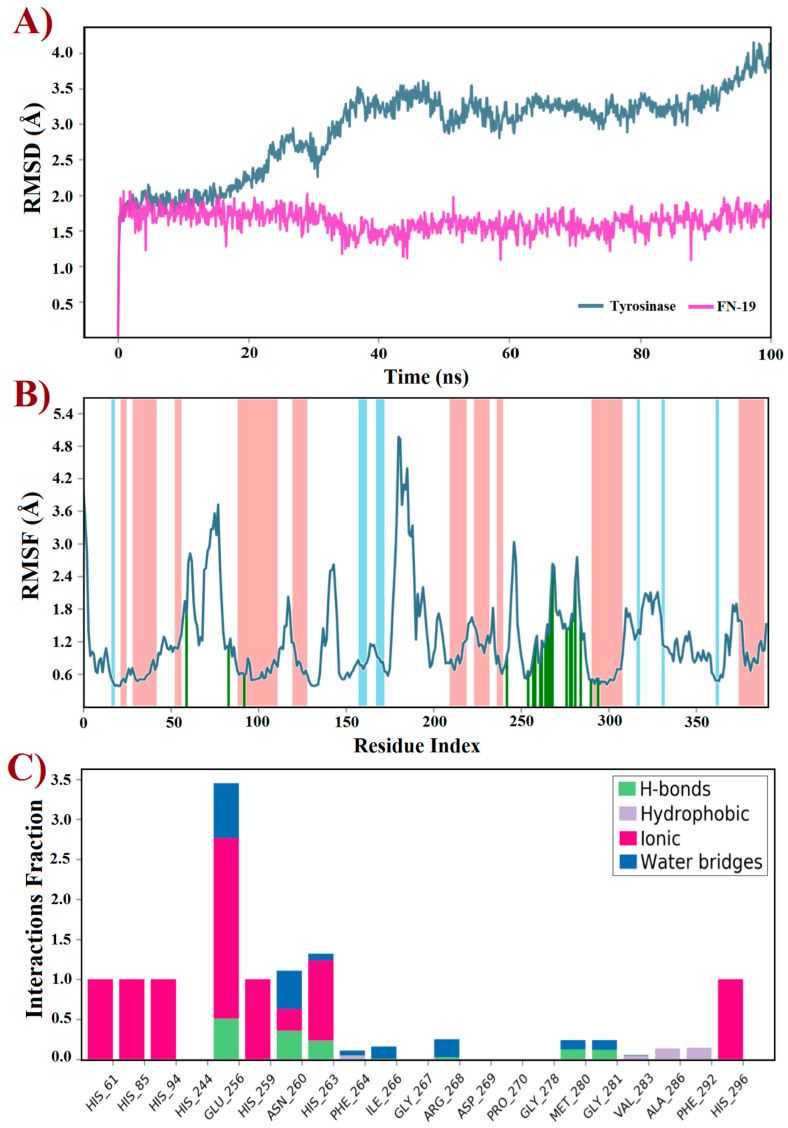
Trajectories for (**A**) RMSD, (**B**) RMSF, and (**C**) interactions fraction obtained using molecular dynamics simulations within 100 ns at 300 K of tyrosinase (PDB id: 2Y9X) complexed with the FN-19 inhibitor. In B, red zones mean alpha-helixes, whereas the beta-sheets are colored in blue; finally, the ligand interactions are shown in green lines.

Molecular docking is an indispensable tool for designing or discovering novel chemical compounds with promising or potential biological properties, which has led to the identification of promising inhibitors targeting tyrosinase [14,55,56,57,58,59]. Typically, docking predicts the binding pose(s) of compounds into a binding site using a scoring function to rank the best of them [60]. Thus, a rigid docking with **FN-19** and tyrosinase was initially performed to generate the ligand–protein complex. Since this in silico uses the target as a rigid structure, being its main limitation, molecular dynamics (MD) simulations emerge as a powerful tool to overcome this issue, providing improved results [61]. This technique predicts the behavior of compounds within a target based on a time-dependent interaction simulation [62]. Thus, it was used to study both free tyrosinase and the **FN19**–tyrosinase complex at a constant pressure of 1 atm, pH 7.4, and temperature of 300 K. Since tropolone is a well-known tyrosinase inhibitor [16,63,64,65], some derivatives have been developed based on the structure of this small seven-membered ring, in which these compounds have been capable of inducing apoptotic cell death of ovarian cancer (OVCAR-3 and OVCAR-8) and colon cancer (HCT-116) by interfering with the ERK signaling pathway [66]. Based on this data, the crystal structure of tyrosinase in complex with tropolone, as an inhibitor, was selected as the target in this study, which was obtained from the Protein Data Bank and coded by PDB id 2Y9X. Then, the tropolone was removed to obtain the apo form of tyrosinase for comparison with the **FN19**–tyrosinase complex. Both free tyrosinase and the **FN19**–tyrosinase complex were submitted to 100 ns MD simulations to investigate their structural stability using the root mean square deviation (RMSD) plot and also other parameters such as the root mean square fluctuation (RMSF) and interactions fraction plots. Initially, the stereochemical quality of the structures generated using MD simulations was checked by analyzing their Ramachandran plots before further studies (see Supplementary Information). For free tyrosinase, it was verified that 86.2% of its residues are placed in the most favored regions, 13.2% in additional allowed regions, and 0.6% in generously allowed regions. Additionally, residues were not found in disallowed regions. For the **FN19**–tyrosinase complex, it was verified that 84.7% of residues are found in the most favored regions, 14.4% in additional allowed regions, 0.6% in generously allowed regions, and 0.3% in disallowed regions, although this unique outlier residue (Asp^3^) does not belong to the binding site of tyrosinase. Thusly, both free tyrosinase and the **FN19**–tyrosinase structures present a great degree of confidence, allowing us to continue our further investigations. The RMSD plot (Figure 4A) was initially generated to describe the stability of atomic systems. Concerning the **FN19**–tyrosinase complex, it was verified that it presents great stability up to 20 ns, then there are several RMSD variations from 21 to 45 ns (Figure 4A). These are possibly associated with the binding of **FN-19** into the tyrosinase, along with protein relaxation. Subsequently, the complex remains stable until 85 ns (characterizing the most stable period of simulation, which was used for further analyses and discussions, after a cluster analysis), which posteriorly decreases its RMSD value and ends with no more alterations. Still, it was verified to have an RMSD_average_ of 0.5 Å, suggesting great stability for the complex. Additionally, it is possible to verify that **FN-19** remains stable during the complete simulated time, with an RMSD_average_ of 0.03 Å. When comparing the **FN19**–tyrosinase RMSD plot with the tropolone–tyrosinase RMSD plot (see Supplementary Information), it is observed that the tyrosinase presents fewer RMSD variations, which was expected since the tropolone presents a small chemical structure, causing minimal steric hindrances (or hydrophobic contacts) in the complexation with the target. The RMSD plot shows the protein deviating from its initial conformation in the first 50 ns and then remaining stable until the end of the simulation. This suggests that the free tyrosinase has a great and constant behavior in the simulated conditions, allowing us to state that its *α*-helixes and *β*-sheets did not undergo huge conformational alterations. Still, tropolone practically did not have any significant RMSD variants within 100 ns MD simulations (see Supplementary Information). Furthermore, the RMSF plot (Figure 4B) revealed the fluctuations in some residues due to the presence of **FN-19** at the binding site of tyrosinase. It is observed that **FN-19** initially induces fluctuations in residues 50–100, 175–200, and 250–300 (green lines) when compared with the tropolone–tyrosinase complex (see Supplementary Information). Additionally, it was verified that **FN-19** mainly interacts with residues comprised from 245 to 295, which are interactions between two *α*-helixes (red zones in the plot). Furthermore, the interactions fraction plot (Figure 4C) was generated to observe the chemical nature of the interactions involving **FN-19** and the tyrosinase binding site. It was suggested that **FN-19** performs mainly ionic (or electrostatic) interactions involving His^61^, His^85^, His^94^, His^259^, His^263^, and His^296^ residues and the hydroxyl group of **FN-19** (acting as a polar group), which should be associated with the complexation with the copper ions. In addition, Glu^256^ and Ans^260^ residues were identified in ionic interactions with **FN-19**. For the tropolone–tyrosinase complex, ionic interactions were also the most frequent intermolecular interactions (see Supplementary Information). In contrast, the hydrophobic interactions are less frequently observed within 100 ns MD simulations. Still, hydrogen-bonding interactions were observed for Glu^256^, Asn^260^, His^263^, Met^280^, and Gly^281^, although with a low frequency. Finally, water bridges were observed for Glu^256^, Ans^260^, His^263^, Phe^264^, Ile^266^, Arg^268^, Met^280^, and Gly^281^. In summary, it was verified that the Glu^256^ residue was found to be the most associated with interactions involving **FN-19**, as expected since its carboxylate group can act by accepting H-bonds with water molecules or other H-donors, and stabilizing positive charges (ionic or electrostatic interactions). In this regard, an in-depth discussion about the **FN-19** interactions will be further provided in the docking section.

### 2.5. Binding Mode Analysis

**Figure 5:**
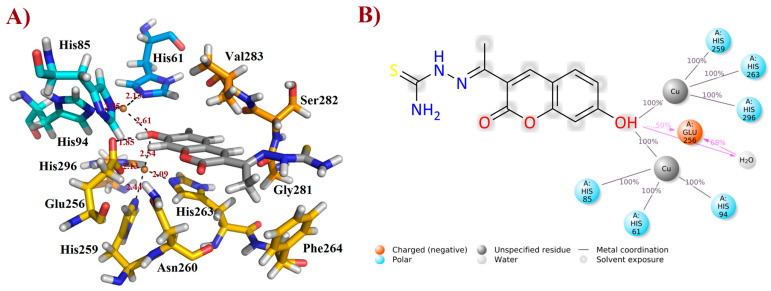
Interactions of FN-19 at the binding site of mushroom tyrosinase enzyme. In (**A**), black dashed lines mean metal coordination and hydrogen bonding interactions, and distance is measured in Ångström. Water molecules were removed from the ligand-target complex for clarification. The illustration was elaborated using PyMOL^®^ v. 0.99. In (**B**), the illustration was created using Maestro^®^ v. 2022.1.

After the MD simulations, the more stable **FN19**–tyrosinase complex was selected for further discussion, based on the aforementioned RMSD plot. Thusly, it was verified that **FN-19** hydrophobically interacts with five amino acid residues, Ans^260^, Phe^264^, Gly^281^, Ser^282^, and Val^283^; whereas a H-bond was observed with Glu^256^ (at a distance of 1.85 Å), in which its hydroxyl group acts as a H-bonding donor, O−H∙∙∙O=C^Glu256^ (Figure 5A), as suggested by MD simulations (see Figure 4C). Additionally, its coumarin core seems to be performing a *π*-stacking interaction with the His^263^ residue, which is complexed with one of the copper(II) ions. Since tyrosinase is a metalloprotein (also known as a copper(II)-containing enzyme), it was verified that the hydroxyl group of **FN-19** seems to be complexed with both Cu^2+^ ions via bidentate coordination, allowing the metallic ions to display a tetracoordinate state, being complexed with three histidine residues and also **FN-19**, with distances of ranging from 2.09 to 2.61 Å. Its oxygen from the hydroxyl group in the coumarin nucleus acts as an electron-donating substituent via its free electron pairs. In general, hydrophobic interactions between tropolone and His^85^, His^259^, Asn^260^, Gly^281^, and Val^283^ residues, and also complexation with Cu^2+^ ion (1.9 Å), have been reported [16], corroborating our findings and suggesting that **FN-19** binds at the same binding site in tyrosinase, as can be seen when superposing both structures (see Supplementary Information). By analyzing the interactions remaining for at least 50% of the 100 ns MD simulations, it is verified that **FN-19** performs bidentate coordination during all simulated times, whereas the hydroxyl group directly interacts with the Glu^256^ residue (for 50% (or 50 ns) of the MD simulation) or via a water bridge (for 68% (or 68 ns) of the MD simulation), as shown in Figure 5B.

### 2.6. Molecular Mechanics Poisson–Boltzmann Surface Area (MM/PBSA) Calculations

MM/PBSA is an efficient and broadly used approach that is able to estimate relatively accurate free binding energies [62,67]. This technique incorporates conformational fluctuations and entropic contributions to binding energies of ligands from MD trajectories [67,68]. MM/PBSA combines (a) changes in potential energies in the vacuum, including both bonded (e.g., bonds, angles, and torsion energies) and non-bonded (van der Waals and electrostatic interactions) terms; (b) the desolvation of the ligand and target, which quantifies the sum of non-polar and polar solvation energies, using an implicit solvation model [69,70,71,72]; and then, (c) the configurational entropy energy related to the complex formation in the gas phase to obtain the free binding energy of molecules complexed into targets [67]. This in silico approach has been used to determine potential inhibitors based on their binding strength with the target [73,74,75]. Then, from the most stable trajectories obtained using MD simulations, MM/PBSA calculations were initially performed for tropolone, a slow-binding and reversible tyrosinase inhibitor [63,64], which was found co-crystallized into the tyrosinase structure (PDB id: 2Y9X), as reported by Ismaya et al. [16]. This is step was required to have a parameter for comparison with the **FN-19** inhibitor. All results are displayed in Table 3, which shows a higher contribution of van der Waals energy (Δ*E_vdW_*) for **FN-19** than tropolone. This was expected since **FN-19** has a larger chemical structure than tropolone, allowing a higher number of hydrophobic contacts. Concerning the electrostatic energies (Δ*E_elec_*), the tropolone presents better electrostatic interactions than **FN-19**, suggesting that the coumarin inhibitor does interact with charged amino acid residues, as observed in the docking analysis. Finally, it was observed that both **FN-19** and tropolone inhibitors have similar binding energy (Δ*E_MM_*) values, suggesting that **FN-19** could be a promising inhibitor of the tyrosinase enzyme.

## 3. Materials and Methods

### 3.1. Chemistry

#### 3.1.1. Synthesis and Characterization of Coumarin-Based Analogs

All reagents and solvents were purchased from commercial suppliers. The reactions were monitored using thin-layer chromatography with silica gel 60 (HF-254 nm, Merck). For column chromatography, Merck silica gel 60G 0.063–200 mm (70–230 mesh ASTM) or 60G 0.2–0.5 mm VETEC^®^ were used. To determine the retention time (R_T_) and purity degree (%), a Shimadzu^®^ (Kyoto, Japan) LC equipment model SIL-20AHT, with a Luna^®^ 5 µm C18(2) 100 Å column (250 × 4.6 mm) was used in wavelengths (λ) of 254 nm. As the mobile phase, methanol HPLC degree (≥99%) was utilized in all HPLC-DAD runs. It was assumed (a) 1 mg/mL for the sample concentration; (b) 1 mL/min for the flow rate; (c) 10 min for the total time; and (d) 5 µL for the volume injected. Finally, all HPLC R_T_ values were computed in minutes (min), while the absorbance was computed as mili-absorbance unities (mAU) [76,77]. Melting points were determined using the MQAPF-301^®^ apparatus and were uncorrected [77,78]. Then, ^1^H and ^13^C NMR, and DEPT-135 NMR spectra were determined in deuterated Acethone-*d*_6_, MeOD, CDCl_3_, or DMSO-*d*_6_ using a Bruker Advanced^®^ DPX spectrometer at 600, 400, 125, and 100 MHz. Chemical shifts (*δ*) are given in parts *per* million (ppm) and coupling constant values (*J*) are given in Hertz (Hz). Signal multiplicities are represented by *s* (singlet), *d* (doublet), *dd* (double doublet), *t* (triplet), *q* (quartet), *m* (multiplet), and *br s* (broad signal) [77,79,80,81]. Finally, all NMR spectra were treated and analyzed using the academic license for Bruker TopSpin^®^ software, version 4.0.8, 2019 (https://www.bruker.com/de/products-and-solutions/mr/nmr-software/topspin.html). Infrared spectra were obtained with an FTIR Thermo Scientific^TM^ Nicolet iS10 spectrophotometer in Attenuated Total Reflectance (ATR) mode. All bond stretches (*v*) and angle deformations (*δ*) for the main functional group from the final compounds were computed in transmittance (T%) and wavenumber (cm^−1^) [82]. Gas-chromatography coupled to mass spectrometry (CG/MS) was used to obtain spectra with chemical ionization at Bruker Amazon SL and data were analyzed in Compass version 1.3. SR2 software (Billerica, MA, USA). All procedures and methods were adapted from Santos-Júnior et al. [83]. All spectra and chemical characterization data can be found in the Supplementary Material of this manuscript. Finally, all experimental procedures described here are in accordance with studies previously published by our research team Cardoso et al. [47] and Silva et al. [81].

#### 3.1.2. General Procedure for the Synthesis of 3-Ketocoumarins and Ethyl-Coumarin-3-Carboxylate Intermediaries

Ethyl acetoacetate (1.5 mmol) or diethyl malonate and piperidine (0.1 mL) in ethanol absolute were mixed with salicylaldehyde derivatives (1.0 mmol). This mixture was stirred at room temperature or reflux for 24–48 hrs. The consumption of the starting material was observed on TLC and, after completion, the reaction was treated with 3.0 mL of 1M HCl solution. The crude precipitated products were filtered and washed with cold water. When necessary, these solids were recrystallized from ethanol or methanol to obtain the ketones **FN-06**, **FN-07**, **MP-03**, **MP-04**, and **MP-05** and an ester analog, **MP-01**. Their melting point, ^1^H and ^13^C NMR, and IR spectra agree with the study previously reported [47].

Description of 3. -Acetyl-7-hydroxy-2H-chromen-2-one (FN-06)

Yield: 76%, yellow amorphous powder; R_T_: 2.95 min; Purity: 99.0%; mp: 240–243 °C; ^1^H NMR (400 MHz, DMSO-*d_6_,* ppm): δ 2.51 (*s*, 3H); 6.71 (*br s*, 1H); 6.82 (*d*, *J =* 8.9, 1H); 7.74 (*d*, *J* = 8.5 Hz, 1H); 8.54 (*s*, 1H); ^13^C NMR (100 MHz, Acethone-*d_6_,* ppm): δ 30.49 (CH_3_); 102.94 (CH); 112.34 (CH); 114.99 (C); 121.09 (CH); 133.35 (C); 148.31 (CH); 158.68 (C); 160.15 (C−OH); 164.79 (C=O); 195.25 (C=O); ATR-FTIR (υ/cm^−1^): 3449 δ(O−H), 1714 *v*(OC=O), 1662 *v*(C=C_ene_), 1600 *v*(C=O), 817 δ(C=C_ene_); CG R_T_: 16.5 min; C_11_H_8_O_4_: 204.18 g/mol; MS: *m/z* 204 [M^+^].

Description of 3. -Acetyl-8-methoxy-2H-chromen-2-one (FN-07)

Yield: 80%, white amorphous powder; R_T_: 2.84 min; Purity: 95.0%; mp: 169–172 °C; ^1^H NMR (400 MHz, Acetone-*d*_6_, ppm): δ 2.61 (*s*, 3H); 3.97 (*s*, 3H); 7.35 (*d*, *J =* 7.6 Hz, 1H); 7.40 (*dd*, *J =* 8.3 and 1.5 Hz, 1H); 7.44 (*dd, J =* 7.6 and 1.6 Hz, 1H); 8.50 (*s*, 1H); ^13^C NMR (100 MHz, Acethone-*d*_6_, ppm): δ 30.62 (CH_3_); 56.66 (OCH_3_); 116.87 (CH); 119.90 (CH); 122.35 (C); 125.65 (CH); 125.93 (C); 145.60 (C); 147.80 (CH); 147.83 (C); 159.33 (OC=O); 195.53 (C=O); ATR-FTIR (υ/cm^−1^): 1687 *v*(C=O), 1726 *v*(C=O), 1566 *v*(C=C_ene_), 860 *δ*(C=C_ene_); CG R_T_: 14.6 min; C_12_H_10_O_4_: 218.21 g/mol; MS: 218 *m/z* [M^+^].

Description of Ethyl 2-oxo-2H-chromene-3-carboxylate (MP-01)

Yield: 49%, white amorphous powder; mp: 89–93 °C; ^1^H NMR (400 MHz, CDCl_3_, ppm): δ 1.36 (*t*, *J* = 7.1 Hz, 1H); 4.36 (*q*, *J =* 7.1 Hz, 1H); 7.32–7.28 (*m*, 2H); 7.63–7.58 (*m*, 2H); 8.49 (*s*, H1); ^13^C NMR (75 MHz, CDCl_3_) δ: 14.2 (CH_3_), 61.9 (CH_2_), 116.6 (C), 117.8 (CH), 118.1 (C), 124.8 (CH), 129.5 (CH), 134.3 (CH), 148.6 (CH), 155.0 (C), 156.8 C=O), 162.9 (C=O). ATR-FTIR (υ/cm^−1^): 1761 *v*(OC=O), 1681 *v*(C=C_ene_), 795 *δ*(C=C_ene_); CG R_T_: 14.3 min; C_20_H_17_NO_3_: 218.20 g/mol; MS: *m/z* 219 [M+H]^+^. All these data are in accordance with Cardoso et al. [47] and He et al. [84].

Description of 3. -Acetyl-6-nitro-2H-chromen-2-one (MP-03)

Yield: 38%, yellow amorphous powder; R_T_: 2.90 min; Purity: 98.1%; mp: 201–203 °C; ^1^H NMR (400 MHz, CDCl_3_, ppm): δ 2.71 (*s*, 3H, CH_3_); 7.50 (*d*, *J* = 9.0 Hz, 1H); 8.48 (*dd*, *J* = 8.9 and 2.3 Hz, 1H); 8.52 (*s*, 1H); 8.56 (*s*, 1H); ^13^C NMR (100 MHz, CDCl_3_, ppm): δ 30.57 (CH_3_); 118.19 (CH); 118.48 (C); 126.00 (C); 126.70 (CH); 128.78 (CH); 144.66 (C); 145.99 (CH); 157.81 (C); 158.63 (C=O); 194.40 (C=O); ATR-FTIR (υ/cm^−1^): 3099 and 3049 *v*(C−H), 1678 *v*(OC=O), 1749 *v*(C=O), 1533 and 1344 *v*(NO_2_), 1556 *v*(C=C_ene_), 819 *δ*(C=C_ene_); C_11_H_7_NO_5_: 233.03 g/mol; CG R_T_: 18.3 min; MS: *m/z* 233 [M^+^].

Description of 3. -Acetyl-7-methyl-2H-chromen-2-one (MP-04)

Yield: 88%, pale green amorphous powder; R_T_: 3.16 min; Purity: 99.8%; mp: 157–159 °C; ^1^H NMR (400 MHz, CDCl_3_, ppm): δ 2.50 (s, 3H, CH_3_); 2.73 (s, 3H, CH_3_); 7.17 (d, J = 8.2 Hz, 1H); 7.19 (s, 1H); 7.55 (d, J = 8.3 Hz, 1H); 8.51 (s, 1H); ^13^C NMR (100 MHz, CDCl_3_, ppm): δ 22.35 (CH_3_); 30.73 (CH_3_); 116.20 (C); 117.04 (CH); 123.62 (C); 126.51 (CH); 130.14 (CH); 146.63 (C); 147.69 (CH); 155.80 (C); 159.70 (C=O); 195.73 (C=O); ATR-FTIR (υ/cm^−1^): 2924 v(C−H), 1732 v(OC=O), 1674 v(C=C_ene_), 1595 v(C=O), 823 δ(C=C_ene_); CG R_T_: 13.4 min; C_12_H_10_O_3_: 202.06 g/mol; MS: *m/z* 203 [M+H]^+^.

Description of 3. -Acetyl-2H-chromen-2-one (MP-05)

Yield: 34%, yellow amorphous powder; R_T_: 3.03 min; Purity: 99.8%; mp: 126–127 °C; ^1^H NMR (400 MHz, CDCl_3_, ppm): δ 2.70 (*s*, 3H, CH3); 7.32 (*t*, *J* = 7.5 Hz, 1H); 7.35 (*d*, *J* = 8.6 Hz, 1H); 7.63–7.62 (*m*, 2H); 8.48 (*s*, 1H); ^13^C NMR (100 MHz, CDCl_3_, ppm): δ 30.70 (CH_3_); 116.92 (CH); 118.52 (C); 124.86 (C); 125.17 (CH); 130.42 (CH); 134.56 (CH); 147.57 (CH); 155.59 (C); 159.41 (C=O); 195.63 (C=O); ATR-FTIR (υ/cm^−1^): 2924 *v*(C−H), 1732 *v*(OC=O), 1676 *v*(C=C_ene_), 1610 *v*(C=O), 823 δ(C=C_ene_); CG R_T_: 12.0 min; C_11_H_8_O_3_: 188.0 g/mol; MS: *m/z* 188 [M^+^]. All these data are in accordance with He et al. [84].

#### 3.1.3. General Procedure for preparation of coumarin–chalcone (FN-10)

Compound **FN-10** was prepared with an aldol condensation reaction using the methodology described by Jagtap et al. [85].

Description of 3. -((E)-3-(4-(Dimethylamino)phenyl)acryloyl)-2H-chromen-2-one (FN-10)

Yield: 49%, red amorphous powder; R_T_: 3.42 min; Purity: 97.5%; mp: 111–112 °C; ^1^H NMR (600 MHz; CDCl_3_, ppm) δ: 3.05 (*s*, 6H); 6.68 (*d*, *J*= 9.0 Hz, 2H); 7.33 (*t*, *J*= 8.0 Hz, 1H); 7.38 (*d*, *J*= 8.0 Hz, 1H); 7.58 (*d*, *J*= 9.0 Hz, 2H); 7.65–7.61 (*m*, 2H); 7.73 (*d*, *J*= 15.7 Hz, 1H); 7.87 (*d*, *J*= 15.7 Hz, 1H); 8.55 (*s*, 1H); ^13^C NMR (150 MHz; CDCl_3_, ppm) δ: 40.1; 111.8; 116.6; 118.7; 122.7; 124.8; 126.1; 129.8; 131.1; 133.7; 146.5; 147.2; 152.3; 155.1; 159.2; 186.0. ATR-FTIR (υ/cm^−1^): 3039 *v*(N(CH_3_)_2_), 1732 *v*(C=O), 1334 and 1166 *δ*(C−N), 1521 *v*(C=C_ene_), 810 *δ*(C=C_ene_); CG R_T_: 29.3 min; C_12_H_10_O_4_: 319.1 g/mol; MS: *m/z* 320 [M+H]^+^. All data are in accordance with Helmy et al. [86].

#### 3.1.4. General Procedure for Preparation of Coumarin–N-acylhydrazone (FN-11)

Coumarin ester (**MP-01**) was dissolved in an 80% hydrazine solution in ethanol. Then, this reactional mixture was refluxed for 2 h. Finally, reflux with the corresponding aldehyde in ethanol for 12 h provides the desired product, **FN-11**. This procedure is in accordance with the methodology described by Cardoso et al. [47].

Description of (E)-N’-(4-(dimethylamino)benzylidene)-2-methylene-2H-chromene-3-carbohydrazide (FN-11)

Yield: 60%, red amorphous powder; R_T_: 3.26 min; Purity: 99.3%; mp: 125–127 °C; ^1^H NMR (600 MHz; CDCl_3_, ppm) δ: 3.04 (*s*, 3H, CH_3_); 6.71 (*d*, 1H, CH_Ar_, *J* = 8.6 Hz); 6.92 (*t*, 1H, CH_Ar_, *J* = 7.4 Hz); 7.00 (*d*, 1H, CH_Ar_, *J*= 8.3 Hz); 7.30–7.32 (*m*, 2H, CH_Ar_ and CH); 8.51 (br s, 2H, NH); 8.71 (s, 1H, OH); 11.96 (s, 1H, NH); ^13^C NMR (150 MHz; CDCl_3_, ppm) δ: 40.1; 111.6; 116.8; 119.2; 121.0; 129.8; 130.4; 131.8; 132.1; 152.6; 159.6; 162.5. ATR-FTIR (υ/cm^−1^): 3197 *v*(N−H), 1699 *v*(C=O), 1604 *v*(C=C_ene_), 1662 *v*(C=O), 1276 *v*(C−O); CG R_T_: 17.9 min; C_19_H_17_N_3_O_3_: 335.13 g/mol; MS: *m/z* 215 [M−120, C_8_H_10_N^•^].

#### 3.1.5. General Procedure for Synthesis of Semicarbazone–, Thiosemicarbazone–, and Isonicotinoylhydrazone–Coumarin Derivatives

Ketone derivatives **(FN-06)** or **(FN-07)** were dissolved in 10.0 mL of ethanol absolute. To this solution, an aqueous-ethanolic (50%) solution of semicarbazide hydrochloride or thiosemicarbazide (1.5 mmol, 5.0 mL) was added. Then, after 5 min, 2 drops of glacial acetic acid were added. The reactions were stirred at room temperature for 6–24 hrs. When the consumption of the starting material was verified with TLC, using dichloromethane and methanol as eluent, the reaction was stopped and left in the refrigerator overnight. Finally, the solids formed were filtered and washed with cold water and ethanol to furnish the desired products **FN-17**, **FN-19**, **FN-25**, **FN-27**, and **FN-29**.

Description of (1E)-1-(1-(7-Hydroxy-2-oxo-2H-chromen-3-yl)ethylidene)semicarbazide (FN-17)

Yield: 60%, yellow-green amorphous powder; R_T_: 3.16 min; Purity: 99.9%; mp: 226–229 °C; ^1^H NMR (400 MHz, DMSO-*d*_6_*,* ppm): δ 2.10 (s, 3H, CH_3_); 6.50 (*s*, 2H, NH_2_); 6.72 (*d*, *J* = 2.2 Hz, 1H); 6.81 (*dd, J* = 8.7 and 2.2 Hz, 1H); 7.58 (*d*, *J =* 8.5 Hz, 1H); 8.23 (*s*, 1H); 9.43 (*s*, 1H, NH); ^13^C NMR (100 MHz, DMSO-*d*_6_*,* ppm): δ 15.89 (CH_3_); 102.18 (CH); 111.95 (C); 114.01 (CH); 122.20 (C); 130.84 (CH); 142.10 (CH); 143.05 (C); 155.74 (C−O); 157.61 (C=N); 160.17 (C=O); 162.00 (C=O). ATR-FTIR (υ/cm^−1^): 3149 *δ*(O−H), 3514 and 3402 *v*(N−H), 1687 *v*(C=O), 1622 *v*(C=C_ene_), 812 *δ*(C=C_ene_); CG R_T_: 16.1 min; C_12_H_11_N_3_O_4_: 261.07 g/mol; MS: *m/z* 218 [M^+^−43, (CH_2_NO^•^)].

Description of (1E)-1-(1-(7-Hydroxy-2-oxo-2H-chromen-3-yl)ethylidene)thiosemicarbazide (FN-19)

Yield: 52%, yellow-orange amorphous powder; R_T_: 2.96 min; Purity: 99.9%; mp: 236–239 °C; ^1^H NMR (400 MHz, DMSO-*d*_6_, ppm): δ 2.22 (*s*, 3H, CH3); 6.73 (*d*, *J* = 2.2 Hz, 1H); 6.83 (*dd, J* = 8.5 and 2.4 Hz, 1H); 7.58 (*d, J* = 8.4 Hz, 1H); 7.91 (*br s*, 1H, NH); 8.36 (*s*, 1H, NH); 8.38 (*s*, 1H); 10.36 (*s*, 1H, NH); ^13^C NMR (100 MHz, DMSO-*d*_6_, ppm): δ 16.09 (CH_3_); 101.78 (CH); 111.44 (CH); 113.70 (C); 121.03 (C); 130.62 (CH); 142.66 (CH); 146.62 (C); 155.53 (C=N); 159.55 (COH); 161.92 (C=O); 179.05 (C=S). ATR-FTIR (υ/cm^−1^): 3340 *v*(N−H), 3236 δ(O−H), 3311 *v*(N−H), 1716 *v*(OC=O), 1683 *v*(C=C_ene_), 1217 *v*(C−N), 813 δ(C=C_ene_); CG R_T_: 15.7 min; C_13_H_13_N_3_O_3_S: 277.30 g/mol; MS: *m/z* 279 [M+2H]^+^.

Description of (1E)-1-(1-(8-Methoxy-2-oxo-2H-chromen-3-yl)ethylidene)semicarbazide (FN-25)

Yield: 46%, yellow amorphous powder; R_T_: 2.77 min; Purity: 98.0%; mp: 240–242 °C; ^1^H NMR (400 MHz, DMSO-*d*_6_, ppm): δ 2.12 (*s*, 3H, CH_3_); 3.91 (*s*, 3H, OCH_3_); 6.52 (*br s*, 2H, NH_2_); 7.30 (*br s*, 3H); 8.30 (*br s*, 1H); 9.52 (*s*, 1H, NH); ^13^C NMR (100 MHz, DMSO-*d*_6_*,* ppm): δ 15.20 (CH_3_); 56.07 (OCH_3_); 114.25 (CH); 119.56 (C); 120.06 (CH); 124.52 (CH); 126.42 (C); 141.05 (CH); 141.92 (C); 142.51 (C); 146.20 (C=N); 156.93 (C−O); 158.90 (C=O). ATR-FTIR (υ/cm^−1^): 3384 *v*(N−H), 3188 *v*(N−H), 1716 *v*(OC=O), 1653 *v*(C=C_ene_), 1681 *v*(C=O), 864 δ(C=C_ene_); CG R_T_: 17.8 min; C_13_H_13_N_3_O_4_: 337.11 g/mol; MS: *m/z* 260 [M−77, C_5_H_4_N^•^].

Description of (1E)-1-(1-(8-Methoxy-2-oxo-2H-chromen-3-yl)ethylidene)thiosemicarbazide (FN-27)

Yield: 46%, yellow amorphous powder; R_T_: 3.42 min; Purity: 97.5%; mp: 220–222 °C; ^1^H NMR (400 MHz, DMSO-*d*_6_*,* ppm): δ 2.24 (*s*, 3H, CH_3_); 3.91 (*s*, 3H, OCH_3_); 7.31 (*m*, 3H); 7.95 (*d*, *J* = 10.0 Hz, 1H); 8.39 (*s*, 1H); 8.44 (*s*, 1H); 10.42 (*s*, 1H); ^13^C NMR (100 MHz, DMSO-*d*_6_*,* ppm): δ 15.89 (CH_3_); 56.10 (OCH_3_); 114.56 (CH); 119.44 (C); 120.18 (CH); 124.60 (CH); 125.83 (C); 142.09 (CH); 142.66 (C); 145.82 (C); 146.23 (C=N); 158.73 (C=O); 179.25 (C=S). ATR-FTIR (υ/cm^−1^): 3456 and 3344 *v*(N−H), 3207 *v*(N−H), 1710 *v*(OC=O), 1612 *v*(C=C_ene_), 1240 and 1097 *v*(C−O), 1220 *v*(C−N), 835 δ(C=C_ene_); CG R_T_: 18.3 min; C_13_H_13_N_3_O_3_S: 291.07 g/mol; MS: *m/z* 291 [M^+^].

Description of (13E)-N’-(1-(8-Methoxy-2-oxo-2H-chromen-3-yl)ethylidene)isonicotinohydrazide (FN-29)

Yield: 64%, orange amorphous powder; R_T_: 2.78 min; Purity: 98.9%; mp: 202–206 °C; ^1^H NMR (400 MHz, DMSO-d_6_, ppm): δ 2.3 (s, 3H); 3.9 (s, 3H); 7.3–7.2 (m, 3H); 7.7 (*d*, *J*= 4.0 Hz, 2H); 8.2 (s, 1H); 8.7 (*d*, *J* = 2.4 Hz, 2H); 11.0 (s, 1H). ATR-FTIR (υ/cm^−1^): 3188 v(N−H), 1703 v(OC=O), 1606 v(C=C_ene_), 1678 v(C=O), 1278 v(C−O), 835 δ(C=C_ene_); CG R_T_: 18.3 min; C_18_H_15_N_3_O_4_: 337.33 g/mol; MS: *m/z* 232 [M−105, (C_6_H_4_NO^•^)].

### 3.2. Mushroom Inhibition Assays

The mushroom tyrosinase (Sigma-Aldrich) inhibition was performed following previously reported methods [43,87]. All assays were carried out with solutions containing phosphate buffer (50 mM, pH 6.8), L-DOPA (0.17 mM), EDTA (0.022 mM), tyrosinase (50–100 units), and varying concentrations of compounds, and were performed in triplicate at room temperature. The inhibitor solutions were prepared in DMSO with an initial concentration of 1 mM. Different aliquots were added to the solution containing buffer, L-DOPA, and EDTA, the enzyme being added in the end. The formation of dopachrome was determined by monitoring the absorbance at 475 nm with a T60U spectrophotometer (PG Instruments) equipped with quartz cells of 1 cm path length. Kojic acid and ascorbic acid were used as reference inhibitors with an initial concentration of 1 mM. The IC_50_ values were calculated from the equation generated with an exponential fit of the experimental data. The effectiveness of inhibition was expressed for the investigated compounds as the percentage of concentration necessary to achieve 50% inhibition (IC_50_), calculated using the following Equation (1):

% of Inhibition = {[(B_30_ − B_0_) – (A_30_ − A_0_)]/(B_30_ − B_0_)} × 100 (1)

where B_0_ = absorbance of L-DOPA + tyrosinase at t = 0 min, B_30_ = absorbance of L-DOPA + tyrosinase at t = 30 min, A_0_ = absorbance of L-DOPA + tyrosinase + inhibitor at t = 0 min, and A_30_ = absorbance of L-DOPA + tyrosinase + inhibitor at t = 30 min.

### 3.3. Kinetic Analysis of the Inhibition of Tyrosinase

A series of experiments was performed to determine the inhibition kinetics of the tested compounds following the already reported method [88,89]. The inhibitor concentrations for the tested compounds were 0.05 and 0.1 mM. The substrate L-DOPA concentration was between 0.1 and 0.25 mM in all kinetic studies, but for the **FN-06** and **FN-27** compounds, concentrations between 0.15 and 0.3 mM were used. Maximal initial velocity was determined from the initial linear portion of absorbance up to ten minutes after the addition of the enzyme. The inhibition type of the enzyme, Michaelis constant (K_m_), and maximal velocity (V_max_) were determined using Lineweaver–Burk plots of the inverse of velocities (1/V) versus the inverse of substrate concentration 1/[L-DOPA] mM^−1^.

### 3.4. Binding Analysis and Molecular Dynamics (MD) Simulations

All molecular docking simulations were performed using a Dell^®^ notebook, (Austin, TX, USA), model 5500U, Intel^®^ CoreTM 5th generation, *i*-7 processer, CPU 2.4 GHz, 16 GB RAM, and running at Windows^®^ 8.1 (Redmond, WA, USA). The *in-house* chemolibrary of coumarins was drawn, converted into three-dimensional structures, and energetically minimized by applying the Parameterized Model 3 (PM3) semi-empirical method, using ArgusLab^®^ v. 4.0.1 (Richland, WA, USA) [90]. The 3D-structure of tyrosinase from *Agaricus bisporus* fungus with tropolone inhibitor (PDB: 2Y9X) was obtained at the Research Collaboratory for Structural Bioinformatics Protein Data Bank (RCSB PDB, San Diego, CA, USA), website (https://www.rcsb.org/structure/2Y9X, accessed on 1 December 2022). GOLD^®^ v. 5.8.1 software (Cambridge, UK) [91] was used to perform virtual screening with docking simulations. Thus, all co-crystallized ligands, water molecules, and ions were removed. The Chemical Piecewise Linear Potential (CHEMPLP) was used as a scoring function for our protocol. Furthermore, all intermolecular interactions (H-bond, hydrophobic, and van der Waals) were individually analyzed using AutoDock Tools v. 1.5.6 (San Diego, CA, USA) [92]. All illustrations were elaborated using PyMol^®^ software, v. 0.99 [93]. Moreover, free tyrosinase and the **FN19**–tyrosinase complex were assumed as start points for our MD simulations to determine their stabilities during the simulated time. Lastly, all these procedures performed in this study are in accordance with recently published works by our research team involving in silico approaches [75,77,94,95,96,97,98,99,100]. All MD simulations were carried out using an Asus^®^ desktop (Taipei, Taiwan), with a Xeon^®^ Core 2^nd^ generation processer, CPU 3.2 GHz, 12 GB RAM, Video card Asus TUF NVIDIA^®^ GeForce RTX 2060—6GB GDDR6 192 bits Dual, 1920 NVIDIA CUDA^®^ cores, and running a Linux^®^ platform. All MD simulations were performed using the Desmond module in Maestro^®^ v. 2022.1 (academic licensed by D. E. Shaw group of companies, https://www.deshawresearch.com/resources.html, accessed on 20 January 2023). Initially, the molecular system was preprocessed by adding missing hydrogens and assuming a pH of 7.4 (PROPKA). The **FN19**–tyrosinase complex was placed into a 10 Å^3^ triclinic box, containing water molecules and Na^+^ and Cl^-^ ions at the physiological concentration (0.15 M). In total, fourteen sodium counterions (Na^+^) were added into the system to obtain electroneutrality. The **FN19**–tyrosinase complex, including water molecules and ions, was minimized using the OPLS2005 force field. The Simple Point Charge (SPC) model was used to describe the water solvation model. The complete system described as a total of 37,034 atoms was subsequently energetically minimized to relax potential steric clashes in the structure, and then the constant Number of particles, Pressure, and Temperature (NPT) equilibration was assumed at 300 K and 1 bar pressure. Posteriorly, MD simulations of 100 ns were performed to obtain data on the stability of the resulting complexes, using a recording interval (trajectory) of 100 ps, and approximately 1000 frames. *UCSF Chimera^®^* software was used to perform a cluster analysis for the **FN19**–tyrosinase complex to obtain the most stable representative structure for further investigations, considering each 5 ns interval of trajectory snapshots comprised within 40–85 ns, which was found to be the most stable period of simulation. Furthermore, Root-mean-square deviation (RMSD), root-mean-square fluctuation (RMSF), and interactions plots were generated using Maestro^®^ v. 2022.1. This protocol is in agreement with other previously published works from our research team [75,96,100,101]. Finally, the stereochemical quality of free tyrosinase and the **FN19**–tyrosinase complex was determined with Ramachandran plots using the web software *SAVES* (https://saves.mbi.ucla.edu/, accessed on 1 February 2023) by applying the *PROCHECK* tool [102].

### 3.5. Molecular Mechanics Poisson–Boltzmann Surface Area (MM/PBSA) Calculations

To perform MM/PBSA calculations, a 100 ns MD dynamics simulation was performed using *GROMACS^®^* v. 2018.3 software [103] to generate *.xtc* and *.tpr* files, which are required for this type of calculation. This protocol is in agreement with other previous studies performed by our research team [75,81]. The most stable MD trajectories (from 40 to 85 ns, see Supplementary Information) were used to obtain the free binding energy for tropolone and **FN-19** in the presence of explicit water, using the MM/PBSA method to estimate their accurate binding energies. To perform MM/PBSA calculations, the *g_mmpbsa* package was used via *GROMACS*, which includes several python scripts to determine the energetic contributions of each amino acid residue in a protein–ligand interaction (http://rashmikumari.github.io/g_mmpbsa, accessed on 1 February 2023). Thus, their binding energy can be decomposed on a per residue basis, in which the energy components *E_MM_,* in the bonded and unbonded forms (Equation (2)) [104,105,106].

(2) $$E_{MM}=E_{bonded}+E_{unbonded}$$

## 4. Conclusions

In this study, a series of coumarin-based compounds were designed, synthesized, and evaluated as inhibitors of the tyrosinase enzyme. Among the compounds tested, **FN-19**, a coumarin–thiosemicarbazone derivative, was the most potent inhibitor. This compound showed better inhibitory activity (IC_50_: 42.16 ± 5.16 µM) than ascorbic acid and kojic acid, which are used as reference compounds, having IC_50_ values of 386.50 ± 11.95 and 72.27 ± 3.14 µM, respectively. However, it was also observed that compounds **FN-06**, **FN-10**, and **FN-27** could be considered promising inhibitors as well. These three coumarin analogs are better tyrosinase inhibitors than ascorbic acid, whereas only **FN-10** showed equivalent activity to kojic acid. Kinetic studies indicated that **FN-19** performs tyrosinase inhibition by a mixed mode, displaying *V_max_* and *K_m_* values of 0.23 and 0.05, respectively. Similarly, **FN-06**, **FN-10**, and **FN-27** are mixed inhibitors of the tyrosinase enzyme, according to the Lineweaver–Burk plots. Still, it was verified that **FN-19** can significantly reduce the signal for L-DOPA oxidation by tyrosinase in UV-vis studies. In addition, the in silico studies involving docking and MD simulations were carried out for this compound. Thus, it was verified that the **FN-19**-tyrosinase complex has good stability throughout the simulated time (100 ns), showing that the ionic ligand–target interactions are the most frequent ones. Furthermore, the binding mode studies (along with MD simulations results) suggested a possible bidentate interaction for **FN-19**, being responsible for its great biological activity, remaining complexed with the copper(II) ions during the entire simulation. Moreover, hydrophobic interactions involving Ans^260^, Phe^264^, Gly^281^, Ser^282^, and Val^283^ residues were observed, while a H-bond is performed with Glu^256^ residue. Finally, the MM/PBSA calculations demonstrated that both **FN-19** and tropolone, a tyrosinase inhibitor, have similar binding energy values of −76.7 ± 0.87 and −78.0 ± 2.64 kcal/mol, respectively. This work developed an in silico and experimental model capable of contributing to a greater elucidation of the interactions between the target and ligand, allowing to understand how **FN-19** interacts at the binding site. Concerning the promising results presented herein, **FN-19** is a promising prototype for additional modification aiming to improve its activity.

## Figures and Tables

**Table 1:** Inhibitory activity of coumarin-based compounds on mushroom tyrosinase.

Compound	IC_50_ [µM]	±SD
**FN-06** 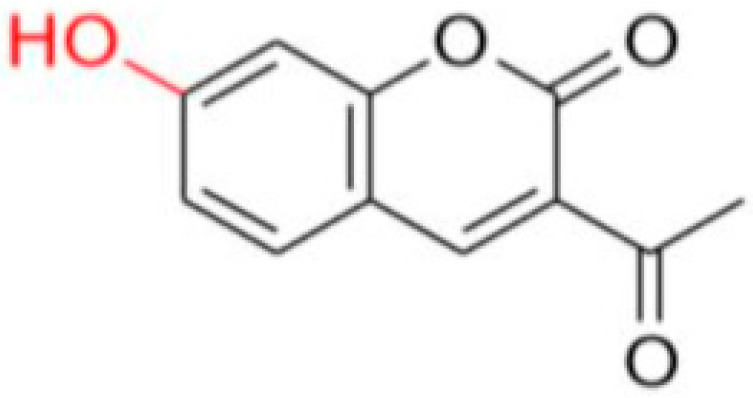	86.72	7.29
**FN-07** 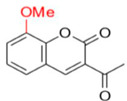	289.12	17.75
**FN-10** 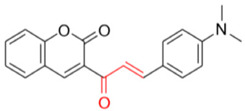	72.40	6.53
**FN-11** 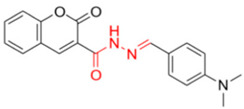	158.65	14.63
**FN-17** 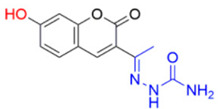	112.79	4.64
**FN-19** 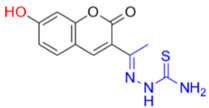	42.16	5.16
**FN-25** 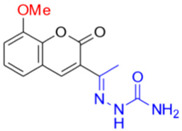	192.35	11.08
**FN-27** 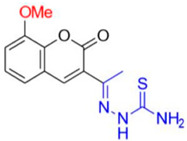	89.75	4.14
**FN-29** 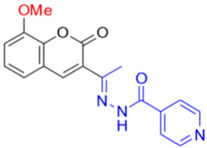	precipitates	
**FN-40** 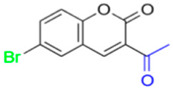	317.82	31.11
**MP-03** 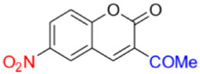	266.71	12.28
**MP-04** 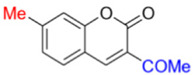	222.03	30.80
**MP-05** 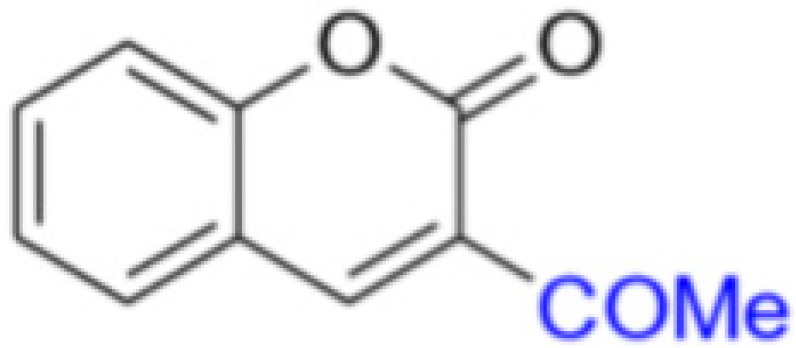	155.68	17.43
**Ascorbic acid**	386.50	11.95
**Kojic acid**	72.27	3.14

**Table 2:** Binding modes of coumarin analogs toward mushroom tyrosinase enzyme.

Compound at 0.1 mM	Inhibitory Mechanism	*V_max_*	*K_m_*
**FN-06**	Mixed	0.97	4.775
**FN-07**	Mixed	28.25	14.98
**FN-10 (0.05 mM)**	Mixed	2.02	0.73
**FN-11**	Mixed	0.44	0.29
**FN-17**	Mixed	1.33	1.73
**FN-19**	Mixed	0.23	0.05
**FN-25**	Mixed	0.96	0.74
**FN-27**	Mixed	0.42	1.15
**FN-29**	Mixed	0.41	0.03
**FN-40**	Competitive	17.68	14.84
**MP-03**	Uncompetitive	1.00	0.52
**MP-04**	Noncompetitive	3.16	1.52
**MP-05**	Mixed	0.50	0.87

**Table 3:** Different MM/PBSA terms for the relative change in the enthalpy of the tyrosinase binding site in complex with tropolone and FN-19 inhibitor at 300 K.

Energy Term	Tropolone (kcal/mol) ± SD	FN-19 (kcal/mol) ± SD
$∆E_{vdW}$	−75.8 ± 6.38	−126.6 ± 10.00
$∆E_{elec}$	−2.2 ± 0.79	49.7 ± 13.10
$\Delta E_{MM}$	−78.0 ± 2.64	−76.7 ± 0.87

Tyrosinase (PDB id: 2Y9X); vdW: van der Waals; elec: electrostatic; SD: standard deviation; MM: molecular mechanics.

## Data Availability

The data presented in this study are available in Supporting Information.

## Supplementary Materials

The supporting information can be downloaded at: https://www.mdpi.com/article/10.3390/ijms24065216/s1.
