# Supplementary material for: Coumarin-Based Compounds as Inhibitors of Tyrosinase/Tyrosine Hydroxylase: Synthesis, Kinetic Studies, and In Silico Approaches
Source: Int J Mol Sci. 2023 Mar 9;24(6):5216. doi: 10.3390/ijms24065216 (PMC10048804; doi:10.3390/ijms24065216)

International Journal of
Molecular Sciences

MDPI

Article

Coumarin-based Compounds as Inhibitors of Tyrosinase/Tyrosine Hydroxylase: Synthesis, kinetic studies, and *in silico* approaches

Jéssica Alves Nunes [1 †], Rodrigo Santos Aquino de Araújo [2 †], Fabrícia Nunes da Silva [3], Joanna Cytarska [4], Krzysztof Z. Łączkowski [4], Sílvia Helena Cardoso [3], Francisco Jaime Bezerra Mendonça-Júnior [2 *], Edeildo Ferreira da Silva-Júnior [1]

[1] *Biological and Molecular Chemistry Research Group, Institute of Chemistry and Biotechnology, Federal University of Alagoas, AC Simões Campus, Lourival Melo Mota Avenue, s/n, 57072-970, Maceió, Alagoas, Brazil.*

[2] *Laboratory of Synthesis and Drug Delivery, Department of Biological Sciences, State University of Paraíba, João Pessoa 58429-500, Paraíba, Brazil.*

[3] *Laboratory of Organic and Medicinal Synthesis, Federal University of Alagoas, Campus Arapiraca, Manoel Severino Barbosa Avenue, Arapiraca 57309-005, Alagoas, Brazil.*

[4] *Department of Chemical Technology and Pharmaceuticals, Faculty of Pharmacy, Collegium Medicum, Nicolaus Copernicus University, Jurasza 2, 85-089 Bydgoszcz, Poland*

* *Corresponding author: Tel.: +55-83-9-9924-1423; e-mail: franciscojaime@servidor.uepb.edu.br (F.J.B. Mendonça-Júnior).*

† The authors contributed equally.

** SUPPLEMENTARY INFORMATION **

SERIE A

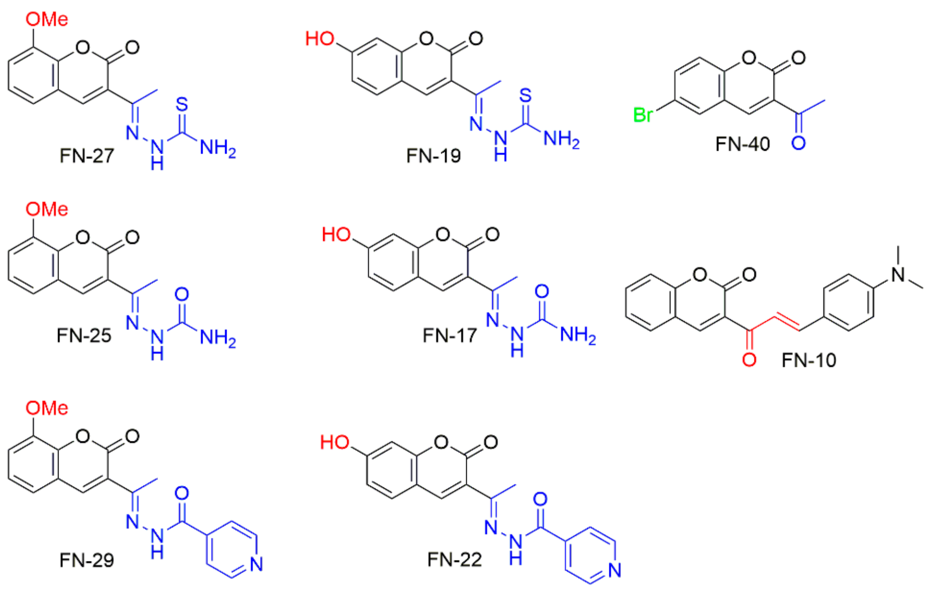

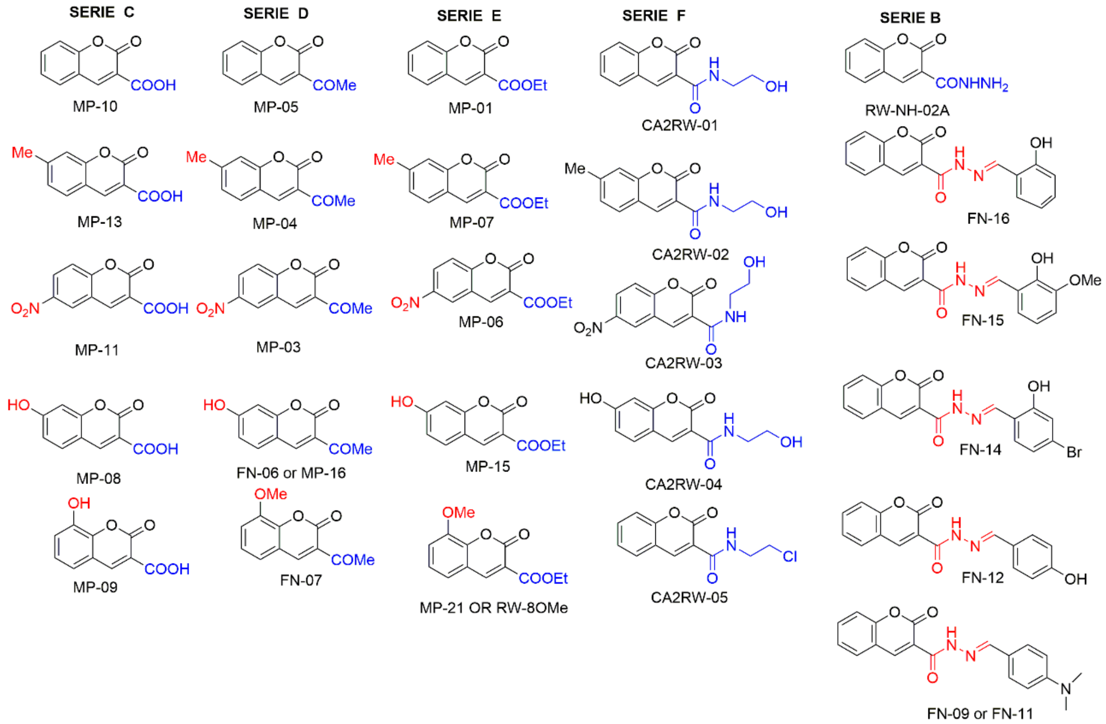

Coumarins-3-cetone derivatives

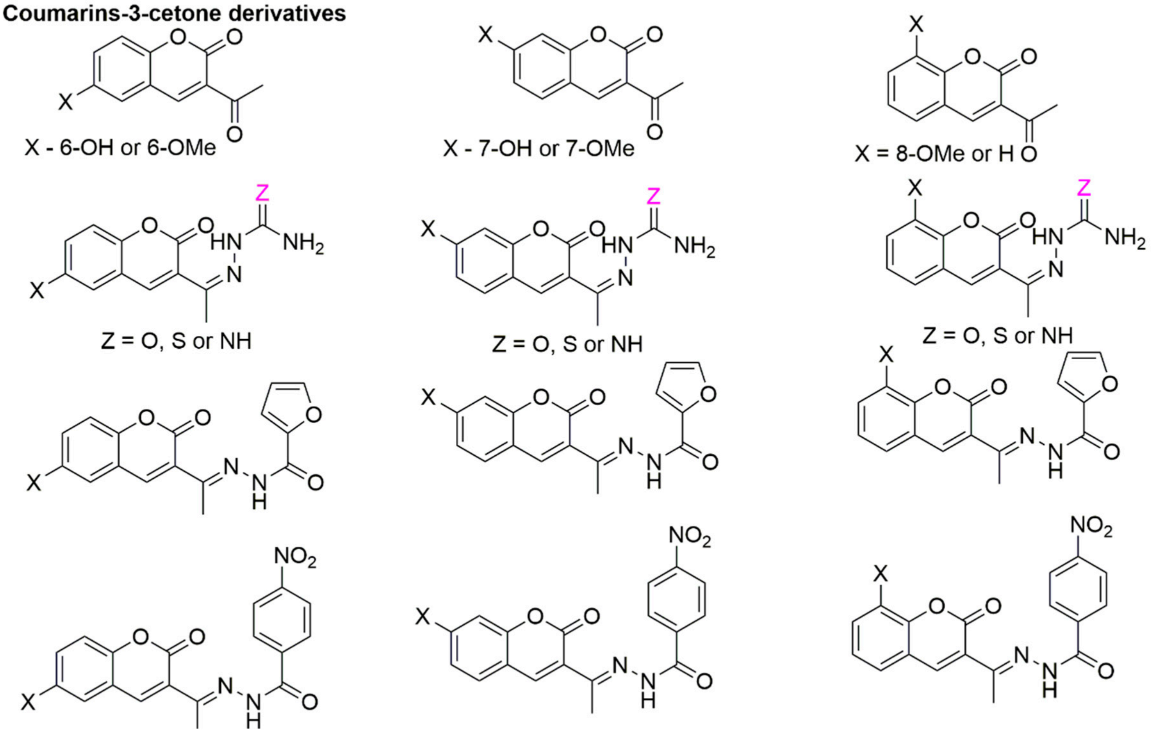

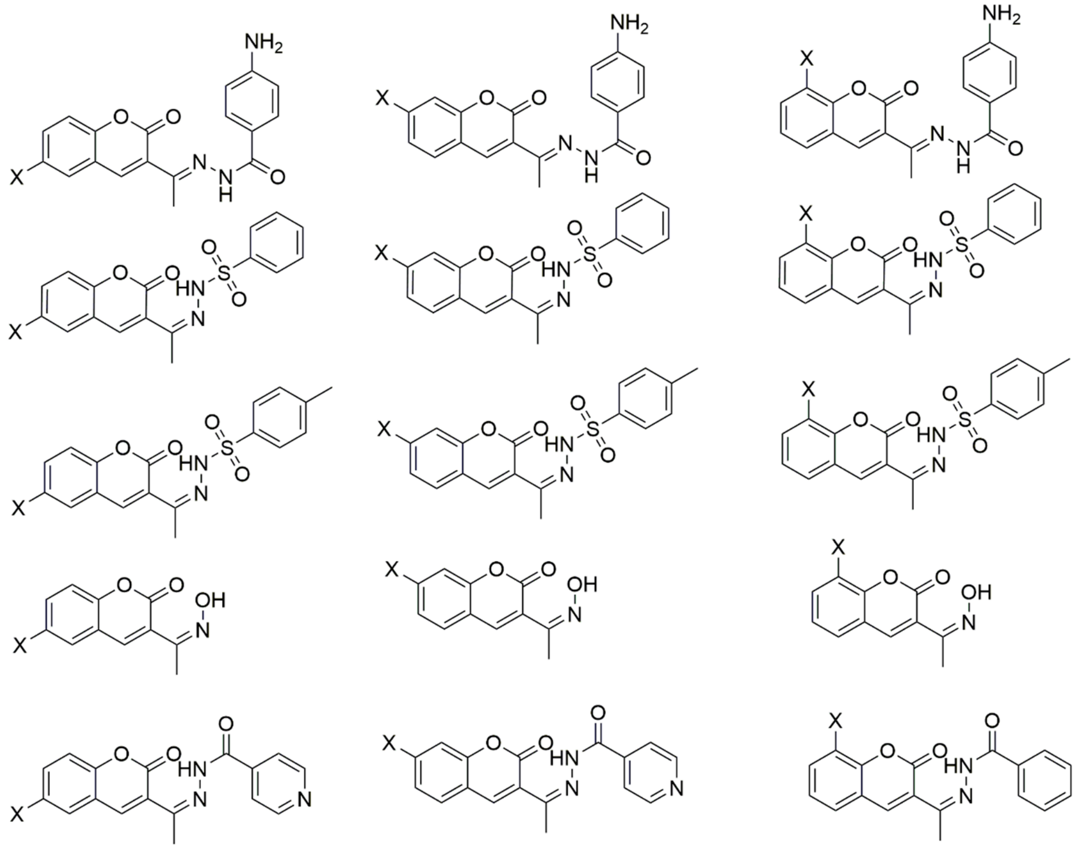

3-coumarins-esthers derivatives

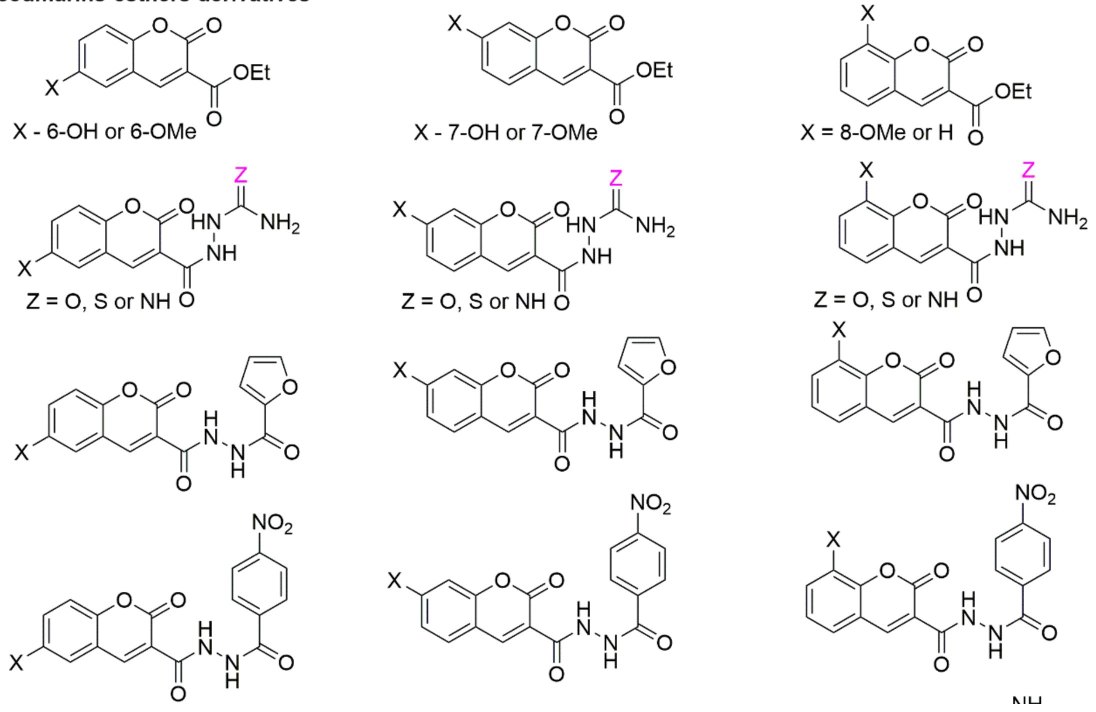

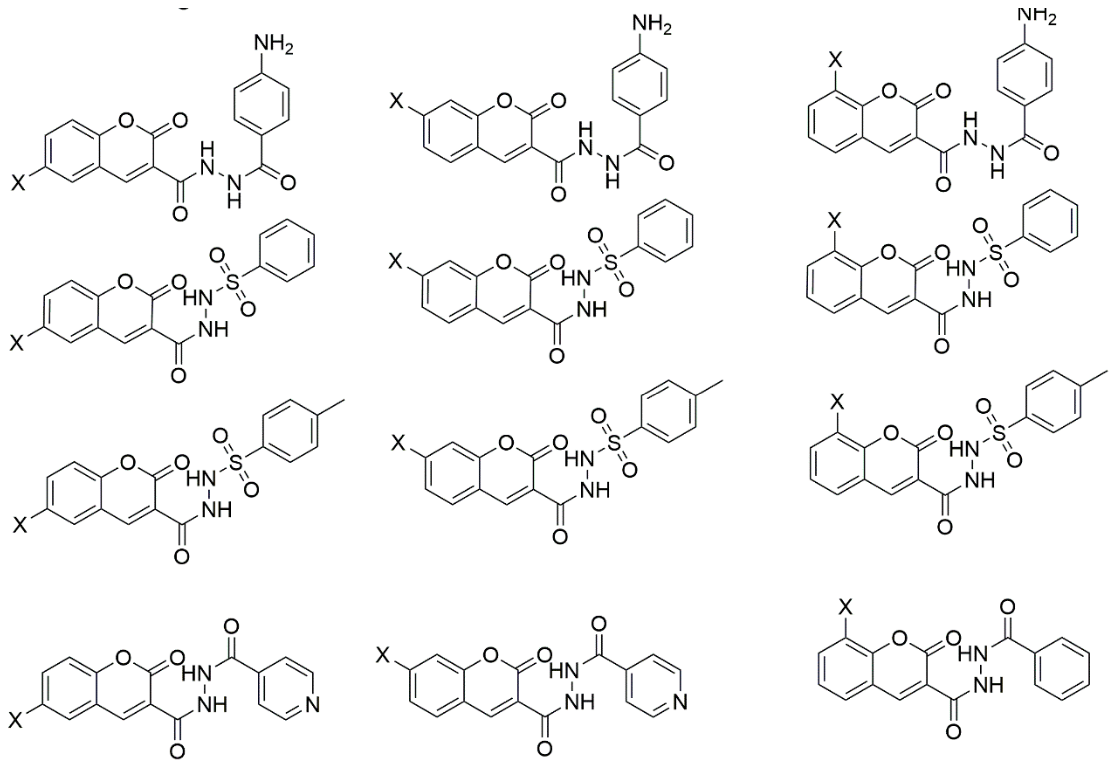

HPLC-DAD chromatograms for the synthesized compounds.

FN-06

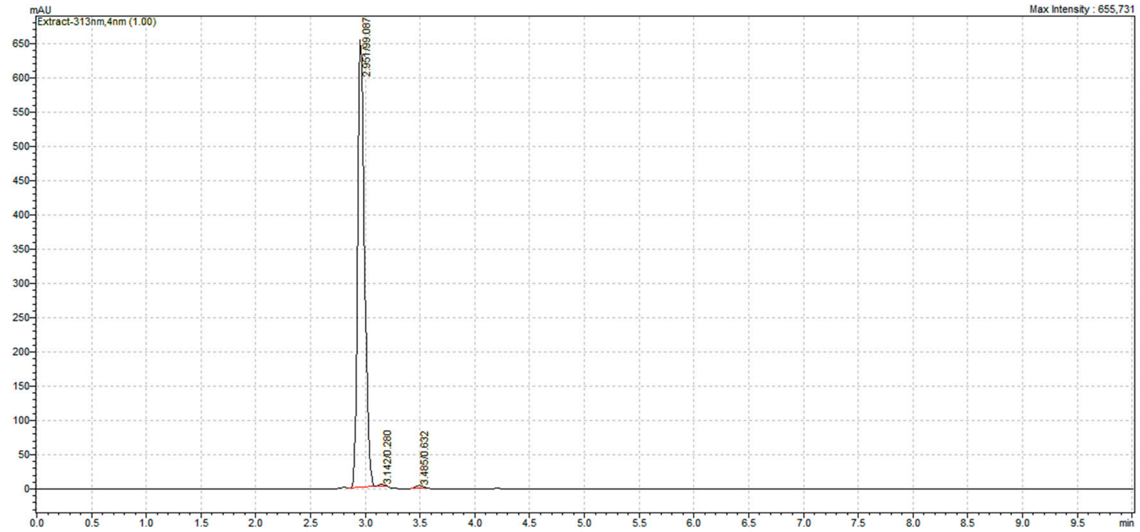

FN-07

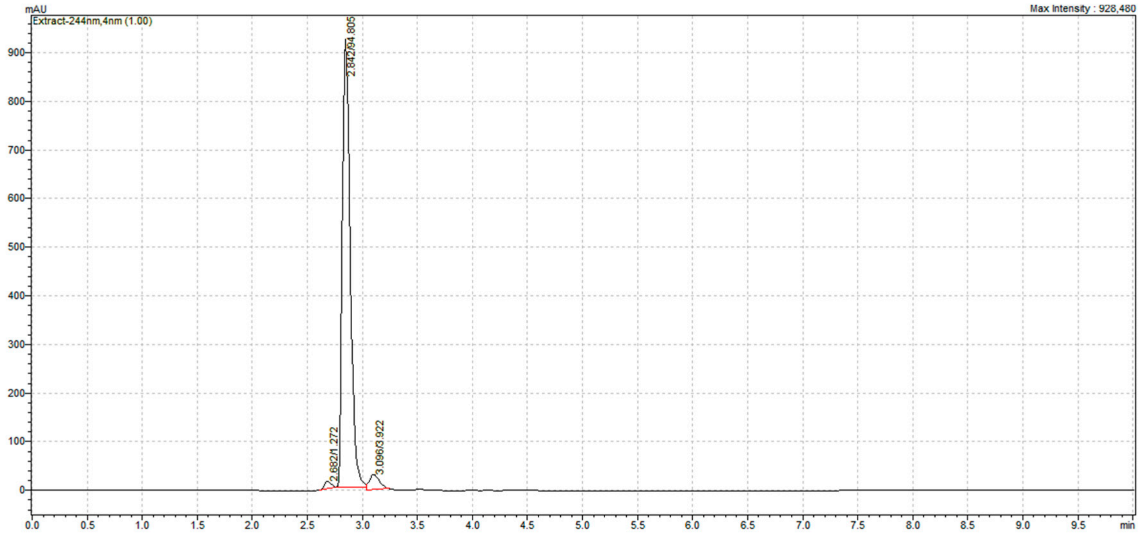

FN-10

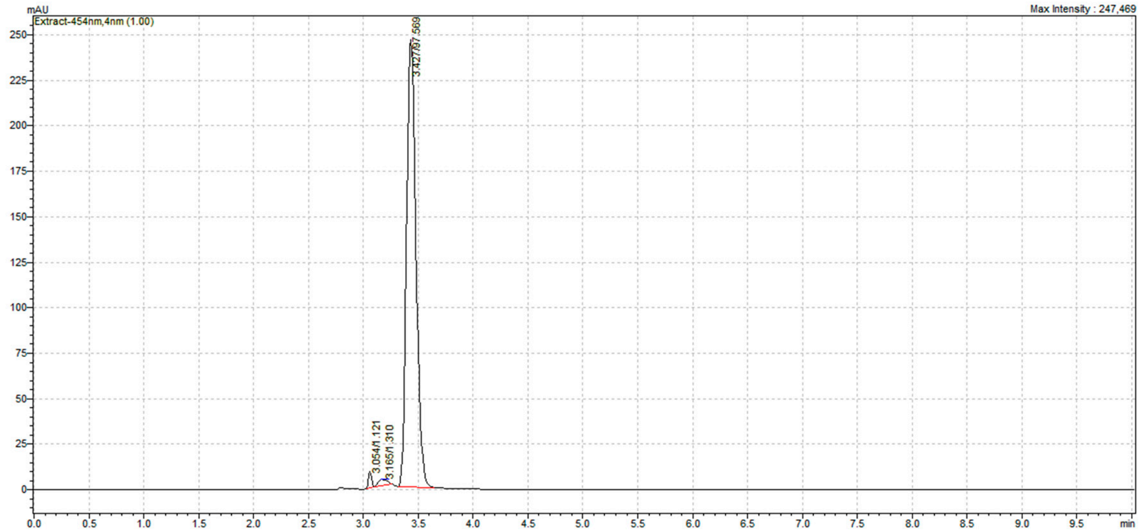

FN-11

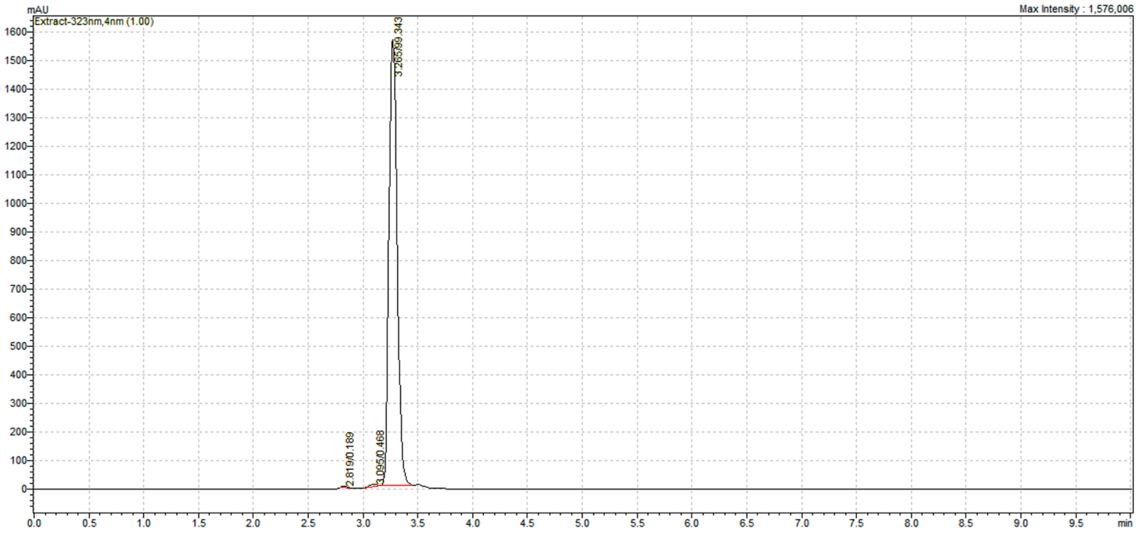

FN-17

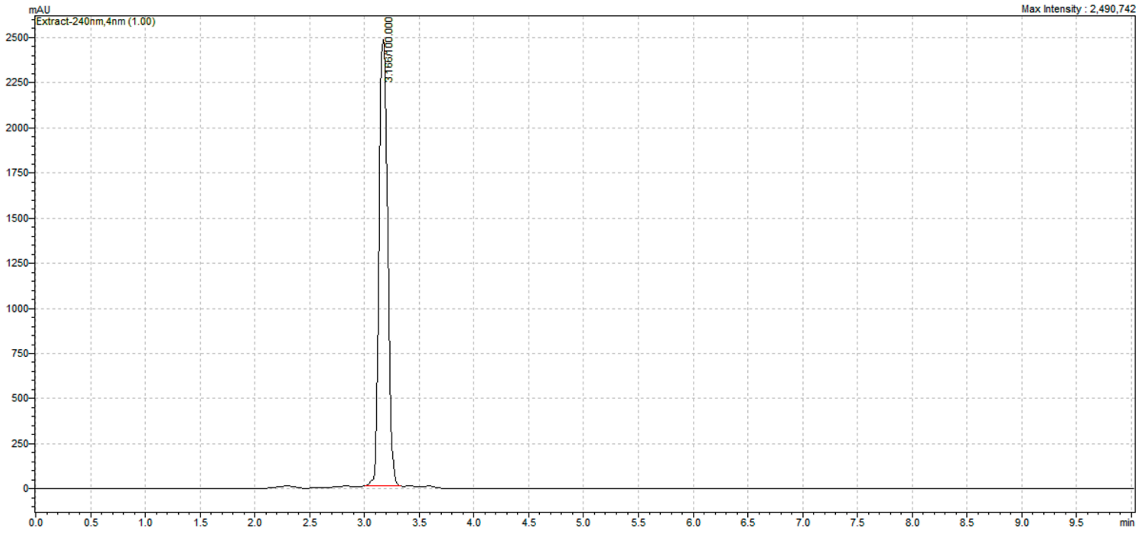

FN-19

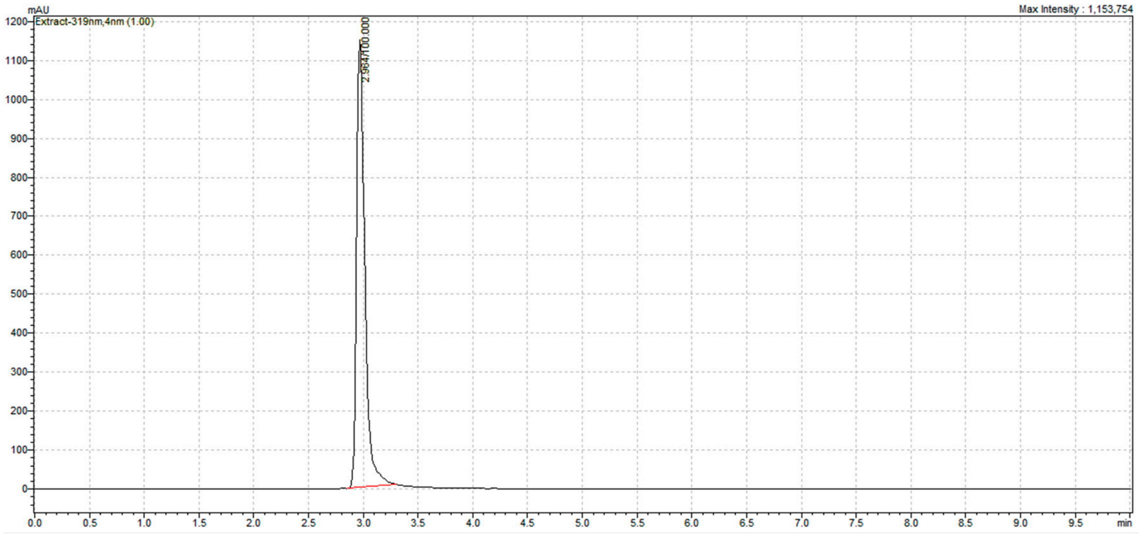

FN-25

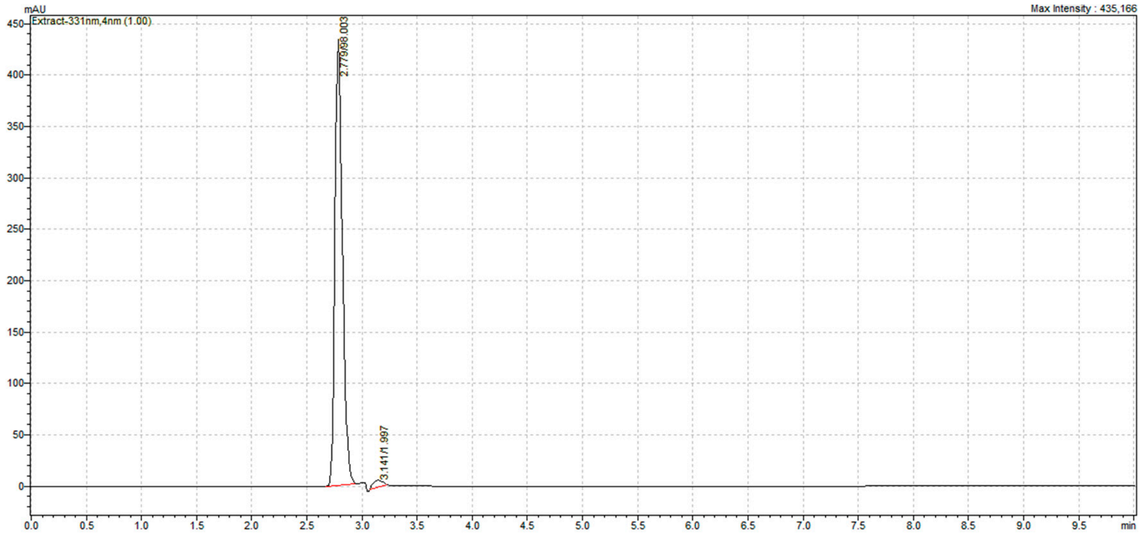

FN-27

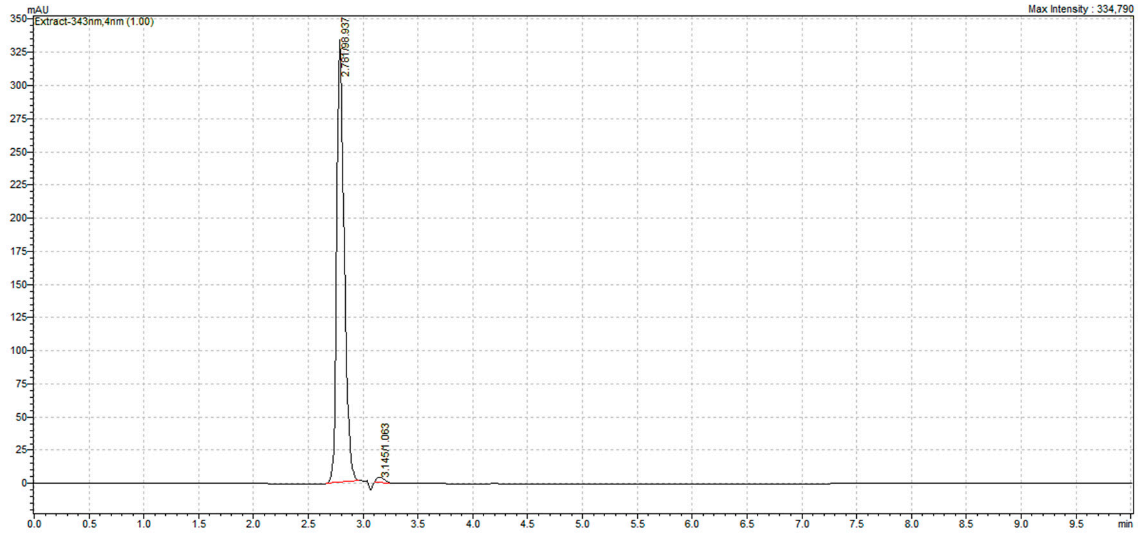

FN-29

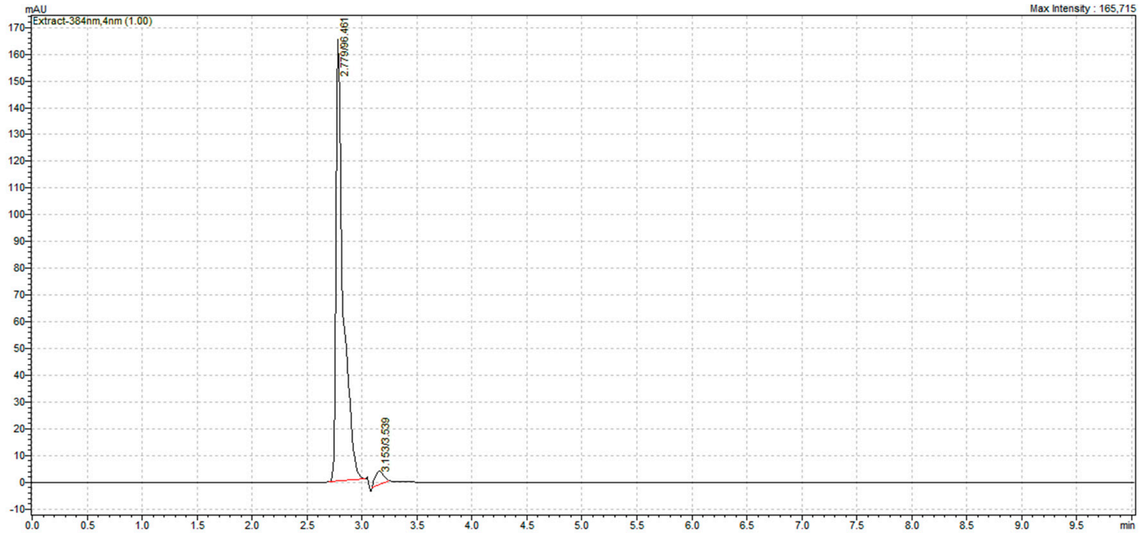

FN-40

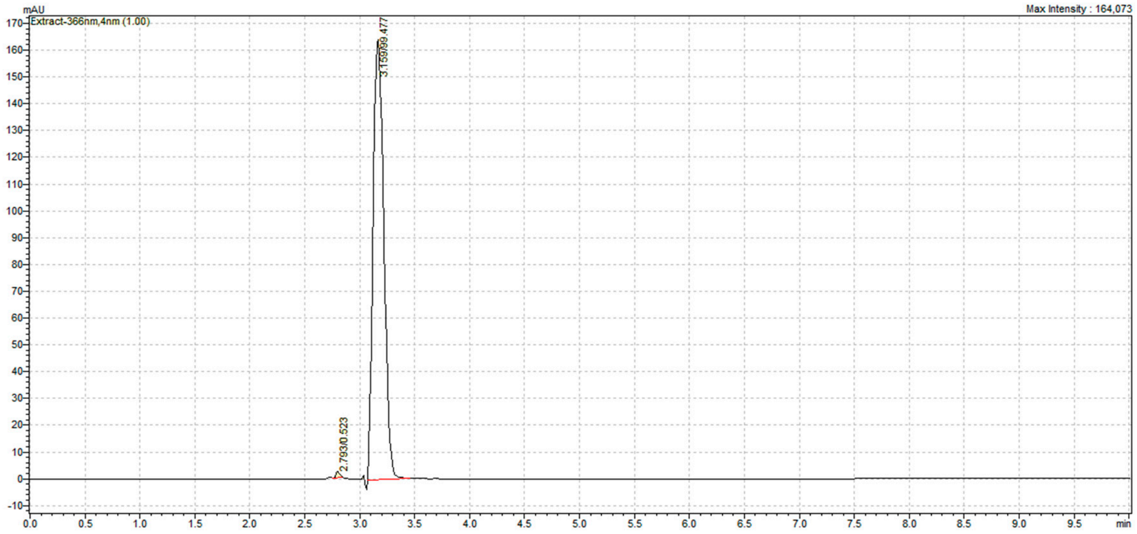

MP-03

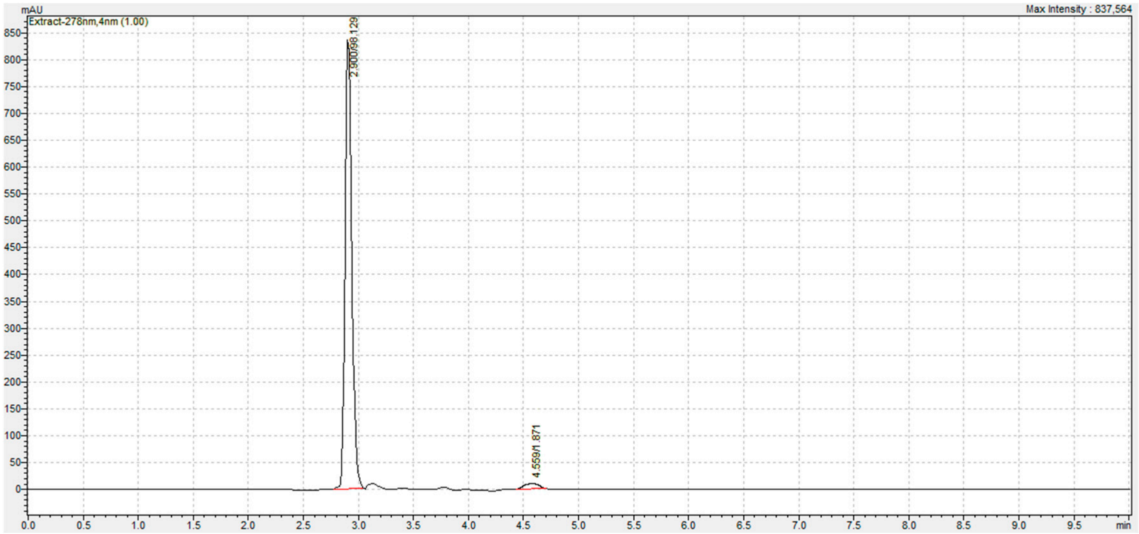

MP-04

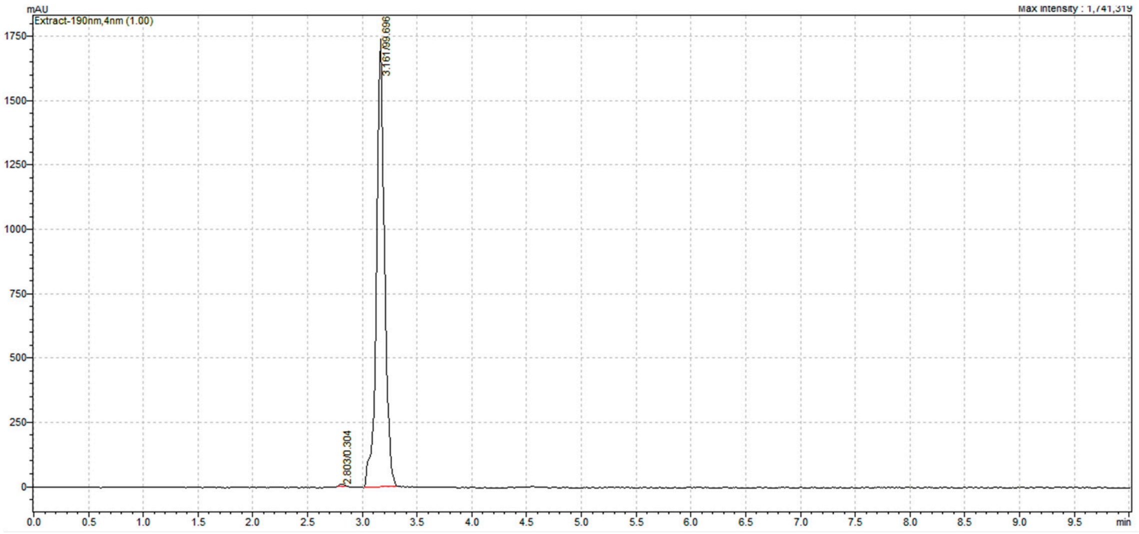

MP-05

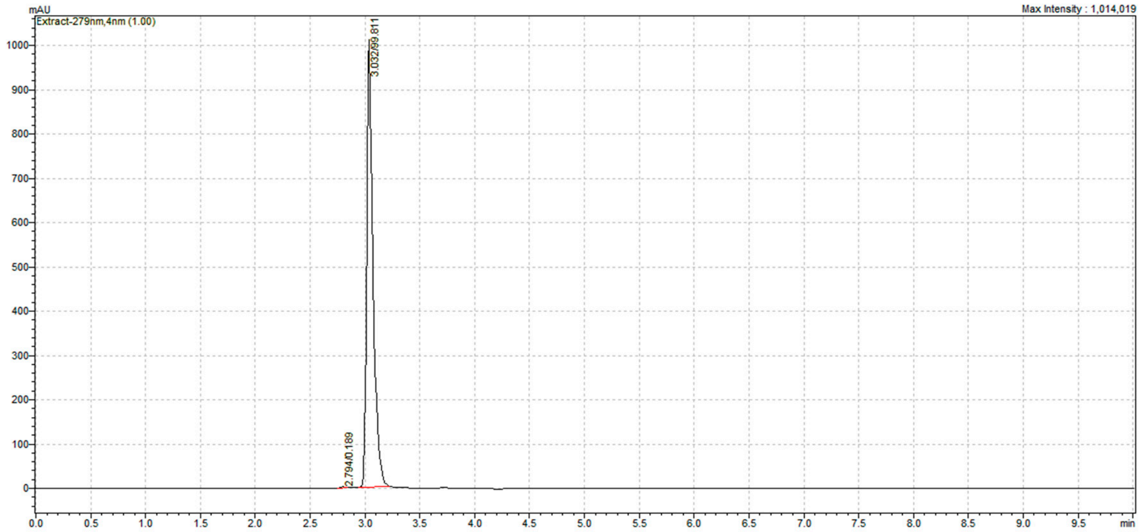

mAU
Extract-279nm,4nm (1.00)
Max Intensity : 1,014,019
3.032/99.811
2.794/0.189
1000
900
800
700
600
500
400
300
200
100
0
0.0
0.5
1.0
1.5
2.0
2.5
3.0
3.5
4.0
4.5
5.0
5.5
6.0
6.5
7.0
7.5
8.0
8.5
9.0
9.5
min

Lineweaver-Burk plots for tyrosinase inhibition

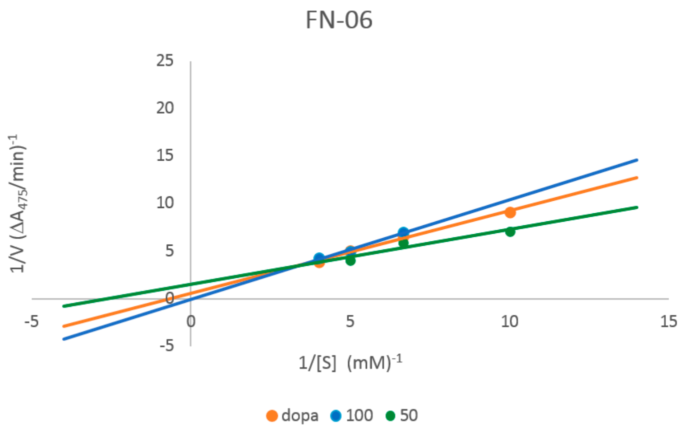

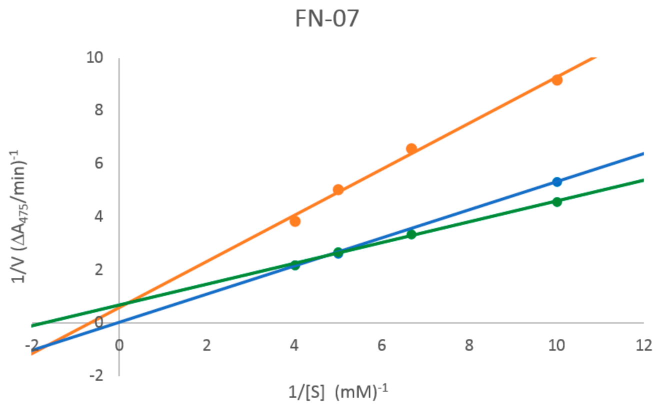

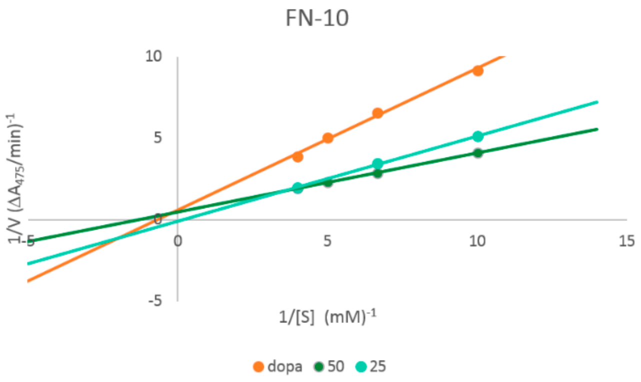

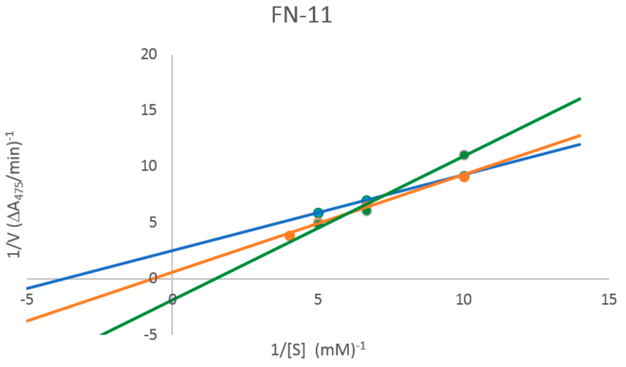

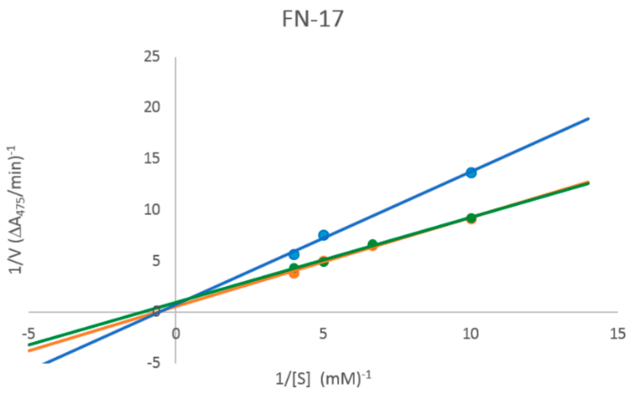

FN-17
1/V (ΔA475/min)-1
1/[S] (mM)-1

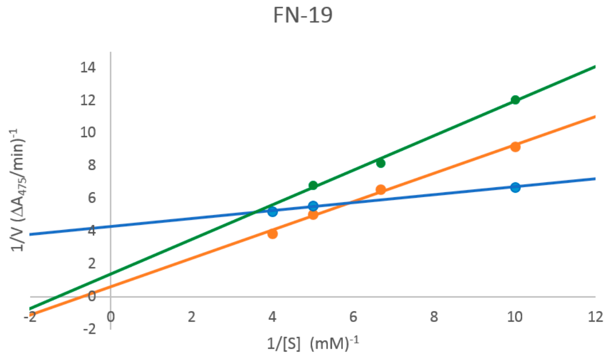

FN-19
1/V (ΔA475/min)-1
1/[S] (mM)-1

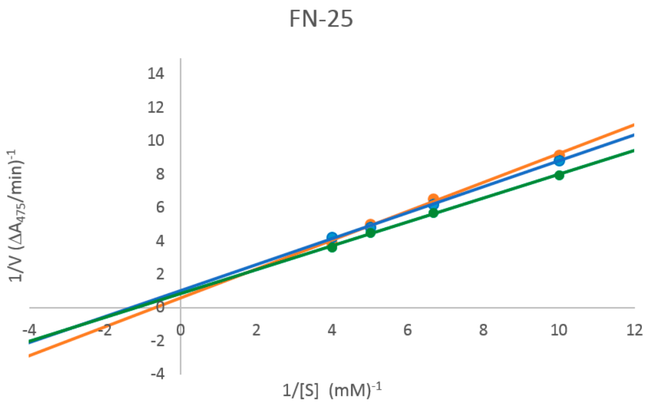

FN-25
1/V (ΔA475/min)-1
1/[S] (mM)-1

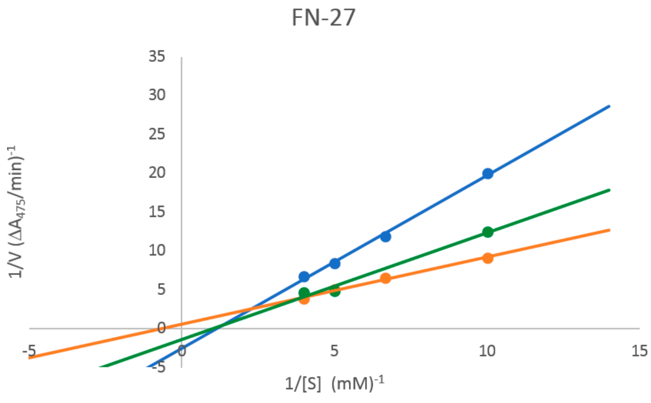

FN-27
1/V (ΔA475/min)-1
1/[S] (mM)-1

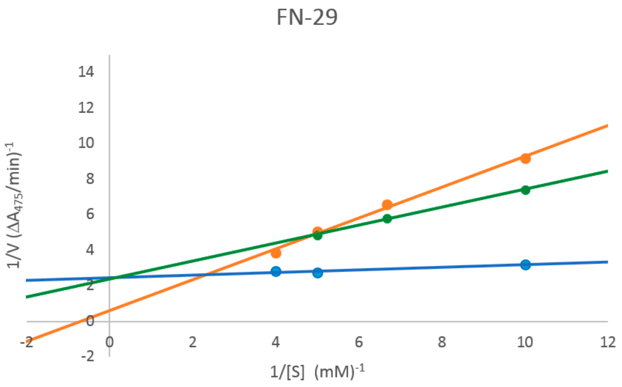
FN-29
1/V (ΔA475/min)-1
1/[S] (mM)-1

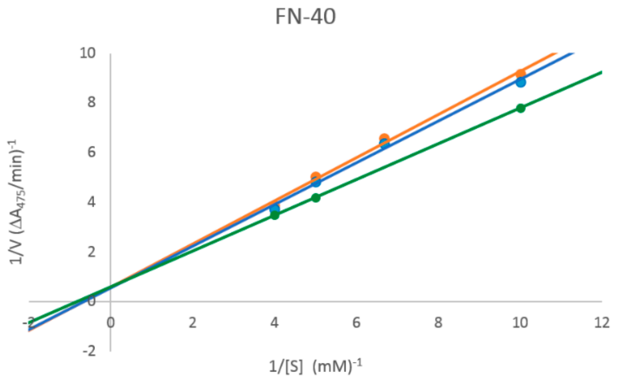
FN-40
1/V (ΔA475/min)-1
1/[S] (mM)-1

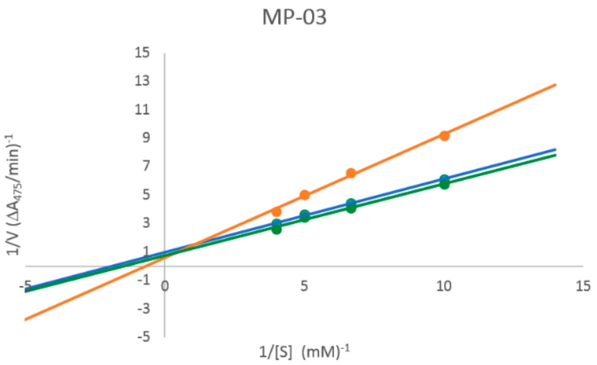
MP-03
1/V (ΔA475/min)-1
1/[S] (mM)-1

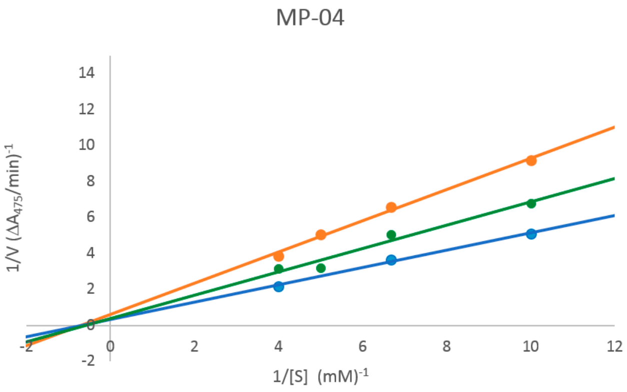
MP-04
1/V (ΔA475/min)-1
1/[S] (mM)-1

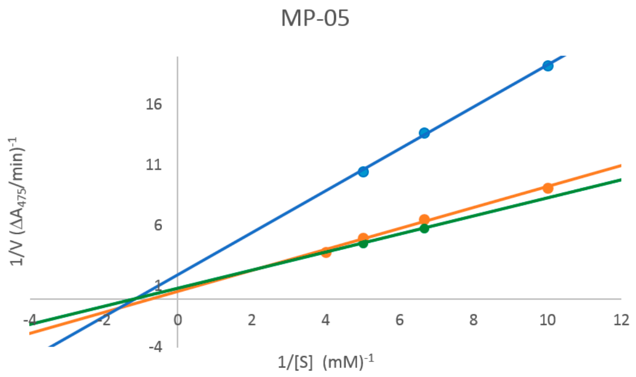

MP-05
1/V (ΔA475/min)-1
16
11
6
1
-4
-4
-2
0
2
4
6
8
10
12
1/[S] (mM)-1

Ramachandran plot for free Tyrosinase within 100 ns MD simulations.

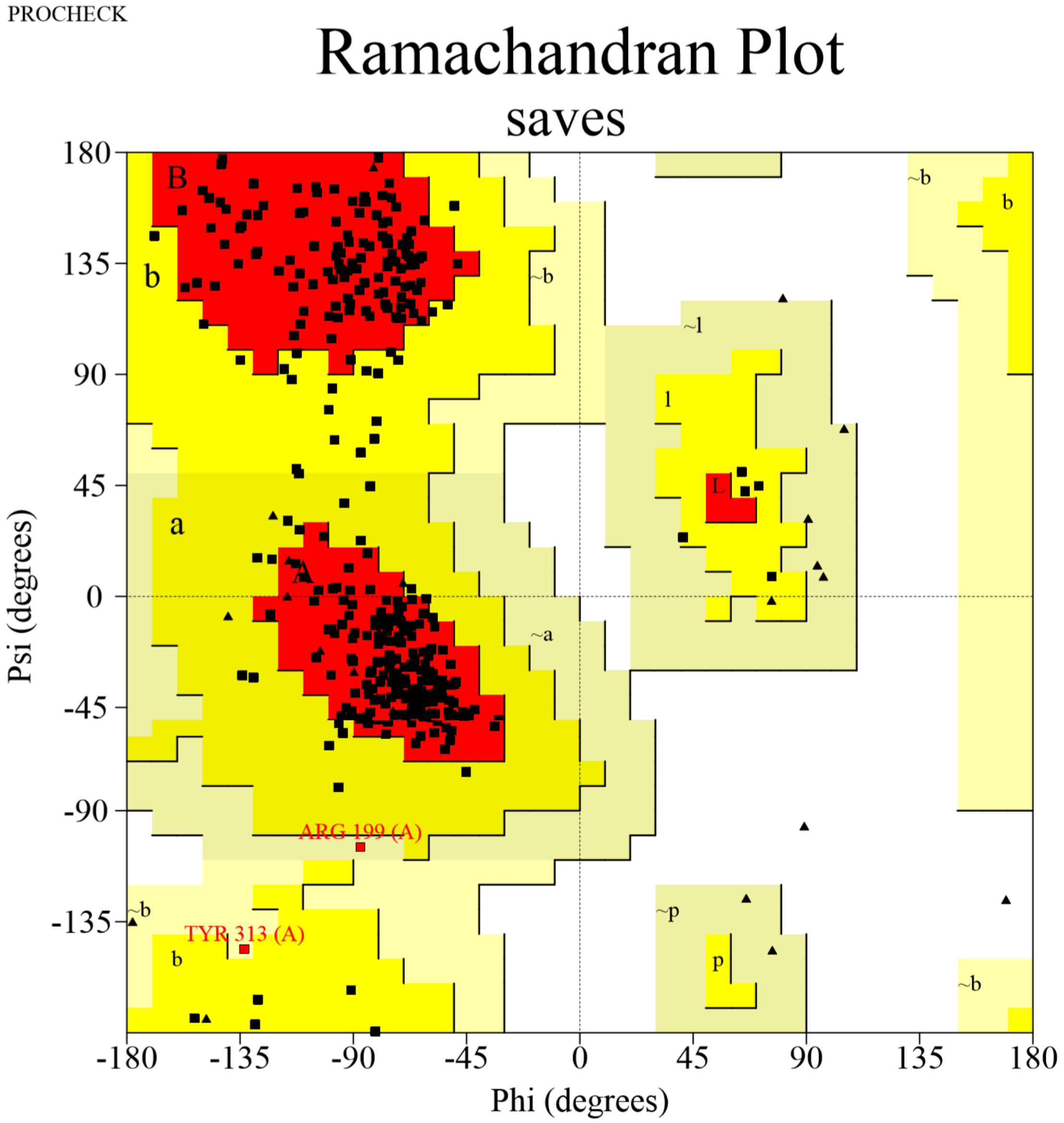

Plot statistics

Residues in most favoured regions [A,B,L]	293	86.2%
Residues in additional allowed regions [a,b,l,p]	45	13.2%
Residues in generously allowed regions [~a,~b,~l,~p]	2	0.6%
Residues in disallowed regions	0	0.0%
	----	------
Number of non-glycine and non-proline residues	340	100.0%
Number of end-residues (excl. Gly and Pro)	4	
Number of glycine residues (shown as triangles)	25	
Number of proline residues	25	

Total number of residues	394	

Based on an analysis of 118 structures of resolution of at least 2.0 Angstroms and R-factor no greater than 20%, a good quality model would be expected to have over 90% in the most favoured regions.

Ramachandran plot for FN19-Tyrosinase complex within 100 ns MD simulations.

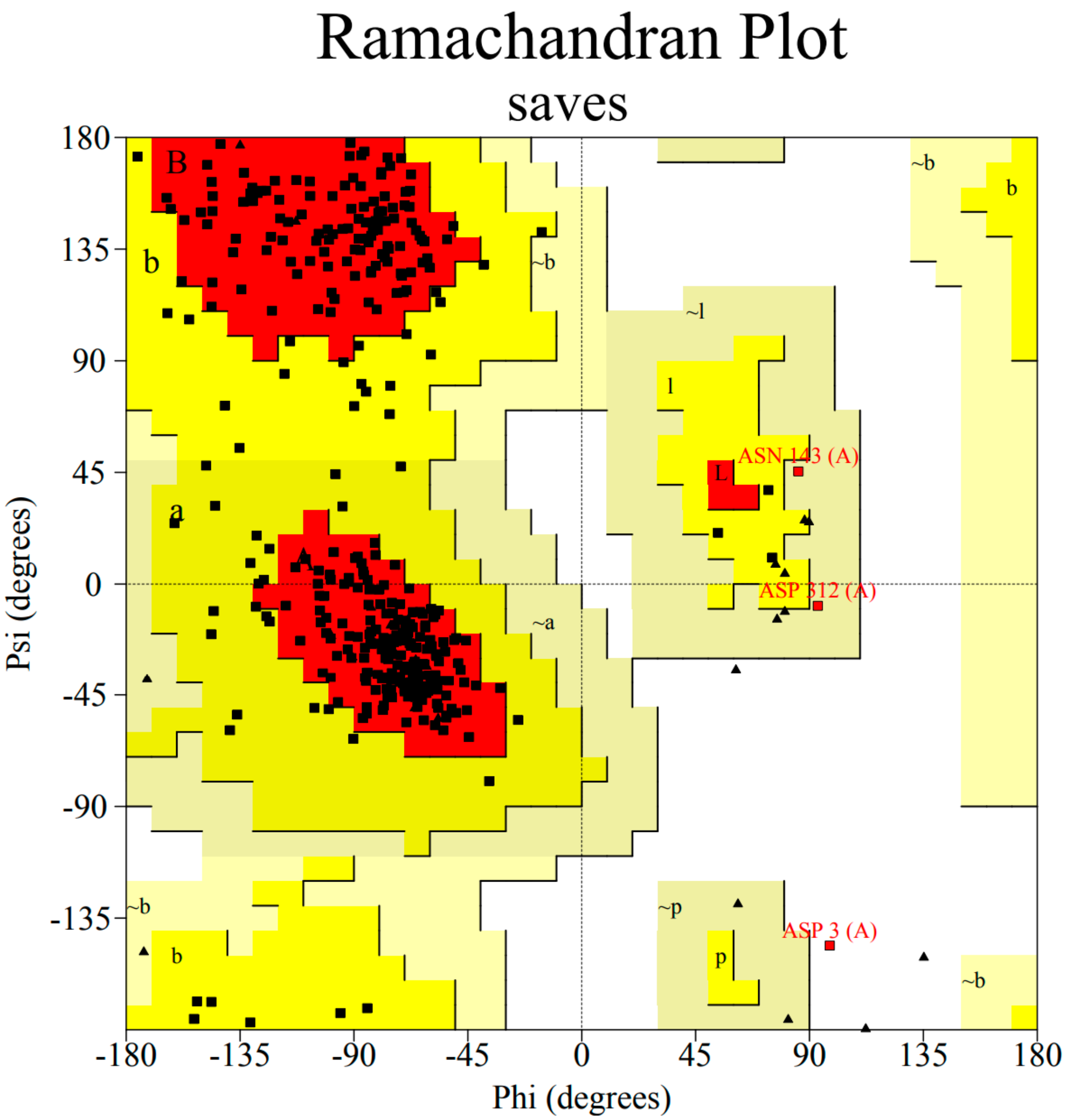

Plot statistics

Residues in most favoured regions [A,B,L]	288	84.7%
Residues in additional allowed regions [a,b,l,p]	49	14.4%
Residues in generously allowed regions [~a,~b,~l,~p]	2	0.6%
Residues in disallowed regions	1	0.3%
	----	------
Number of non-glycine and non-proline residues	340	100.0%
Number of end-residues (excl. Gly and Pro)	4	
Number of glycine residues (shown as triangles)	25	
Number of proline residues	25	

Total number of residues	394	

Based on an analysis of 118 structures of resolution of at least 2.0 Angstroms and R-factor no greater than 20%, a good quality model would be expected to have over 90% in the most favoured regions.

RMSD plot for tropolone-tyrosinase complex within 100 ns MD simulations.

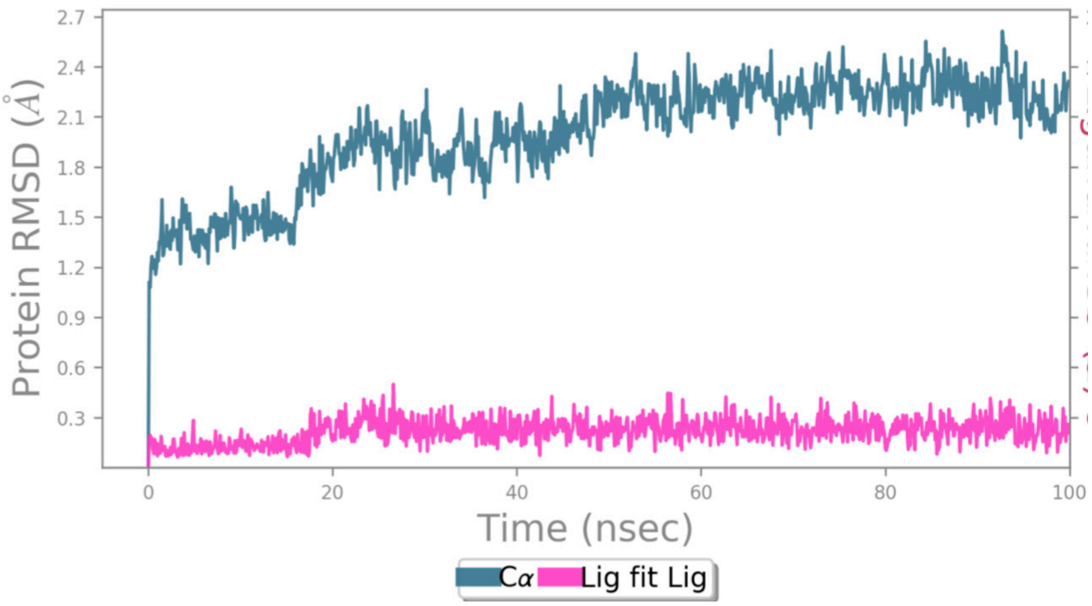

RMSF plot for tropolone-tyrosinase complex within 100 ns MD simulations.

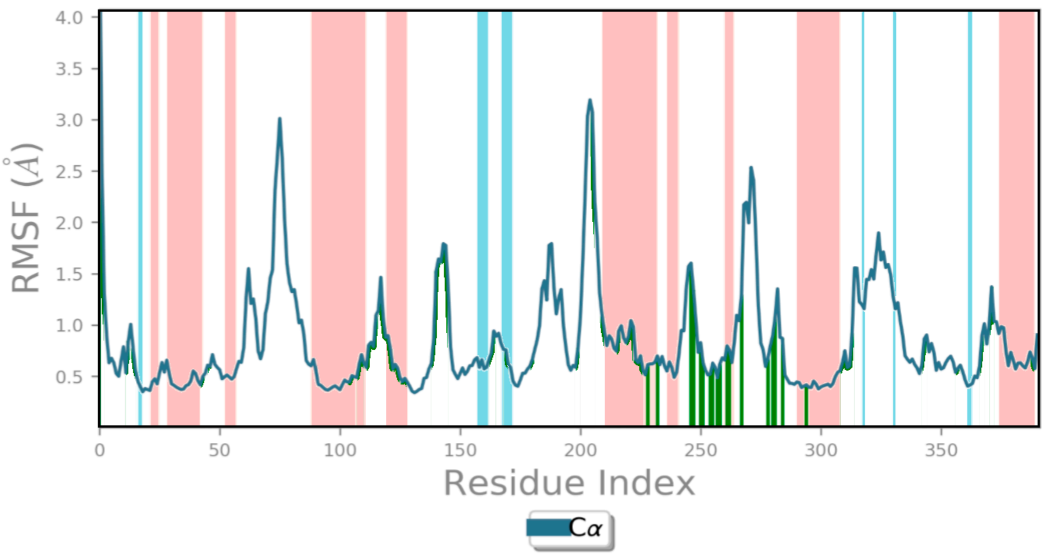

Interactions fraction plot for tropolone-tyrosinase complex within 100 ns MD simulations.

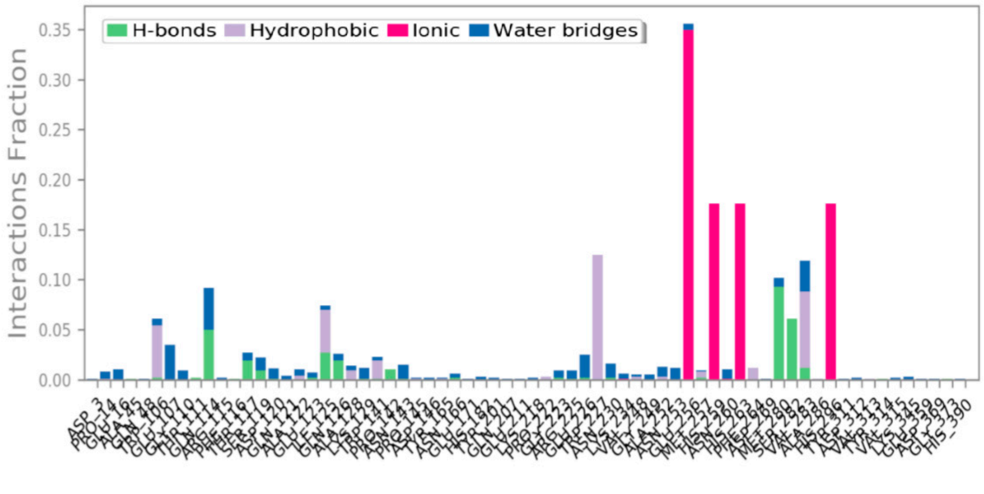

PDB tyrosinase and post-MD tyrosinase superposed.

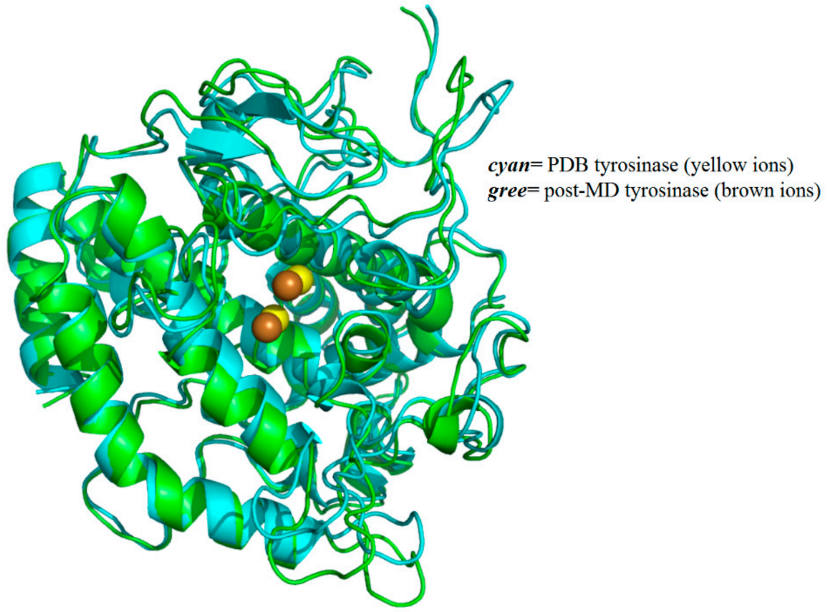

Chemical Characterization

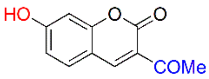

FN-06

*3-Acetyl-7-hydroxy-2H-chromen-2-one **(FN-06)***

ATR-FTIR

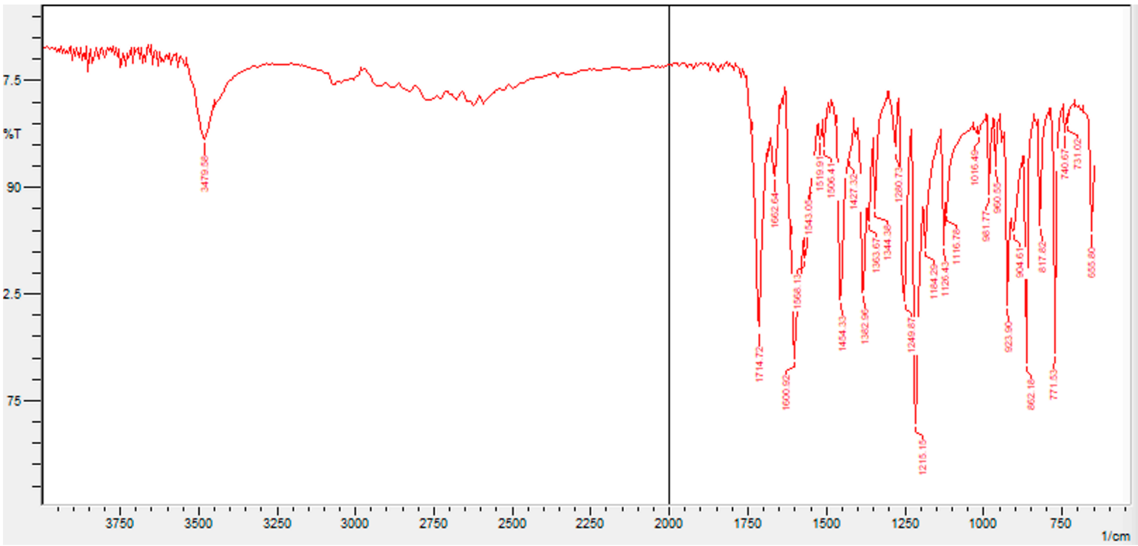

^{1}H NMR (400 MHz, DMSO-d_6, ppm)

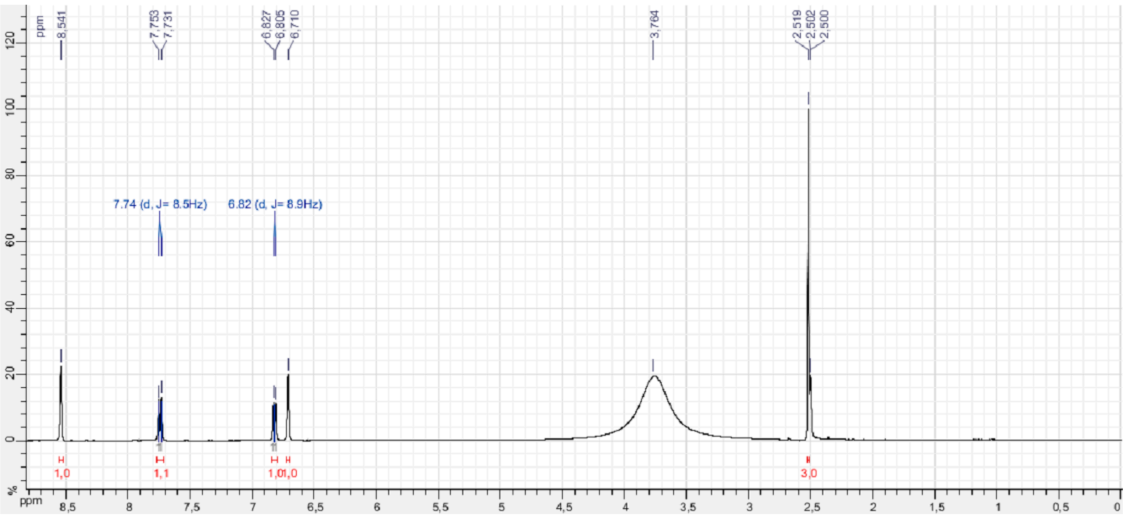

^{13}C NMR (100 MHz, Acethone-d_6, ppm)

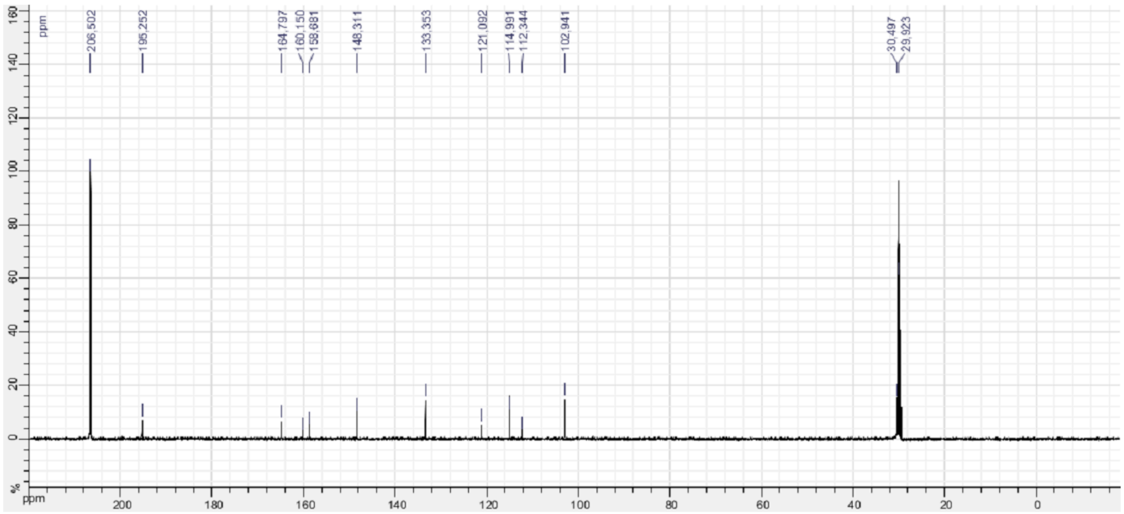

Line#:1 R.Time:16.567(Scan#:1389)
MassPeaks:34
RawMode:Averaged 16.558-16.575(1388-1390) BasePeak:203(641122)
BG Mode:Calc. from Peak Group 1 - Event 1 Scan

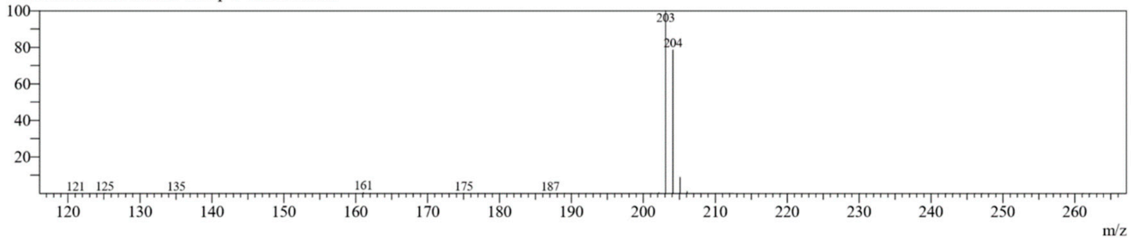

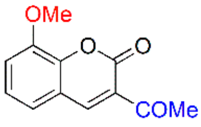

FN-07

3-Acetyl-8-methoxy-2H-chromen-2-one ***(FN-07)***

ATR-FTIR

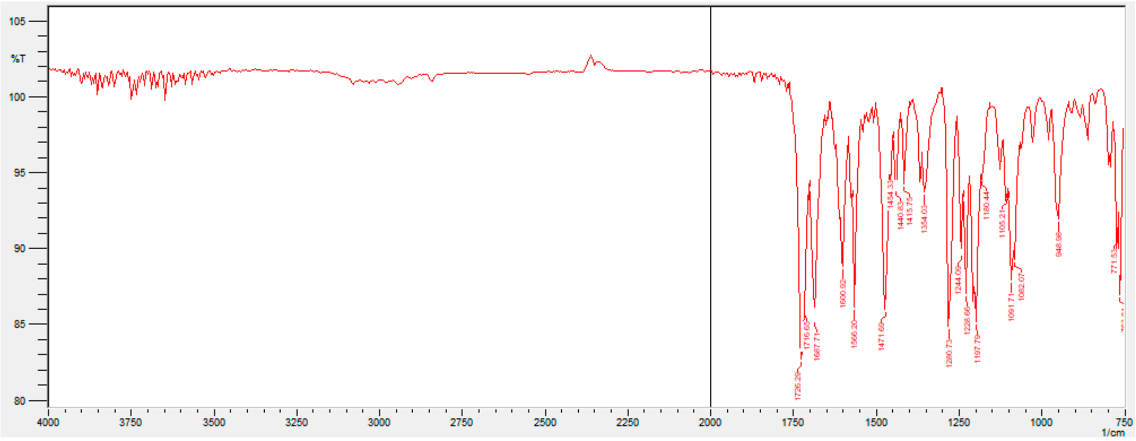

^{1}H NMR (400 MHz, Acethone-d_6, ppm)

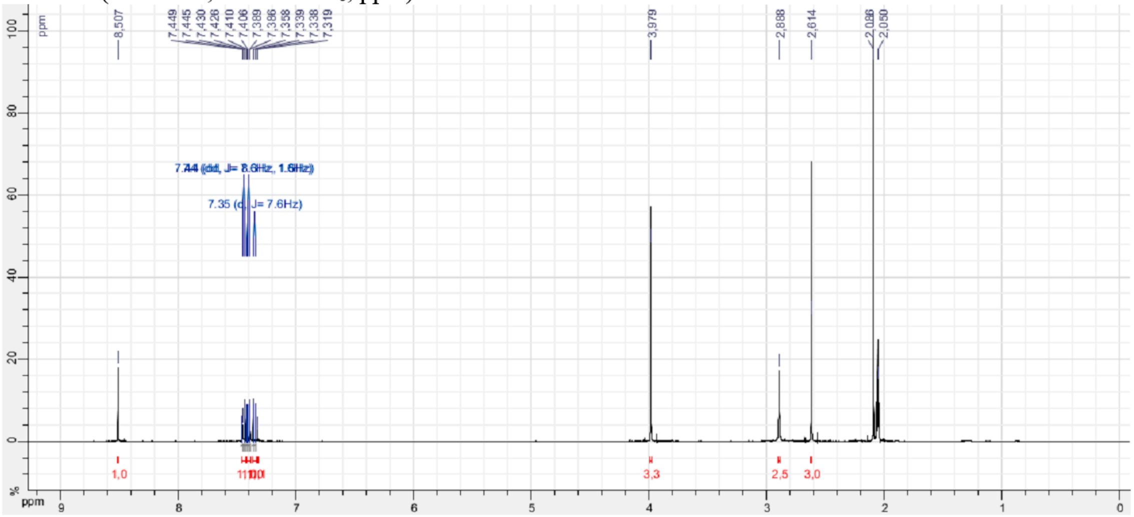

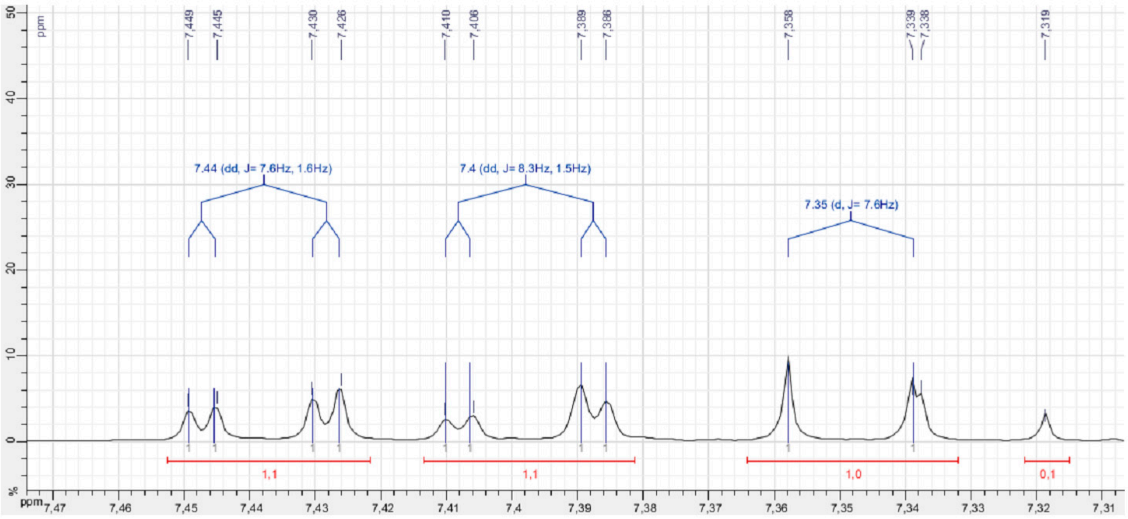

^{13}C NMR (100 MHz, Acethone-d_6, ppm)

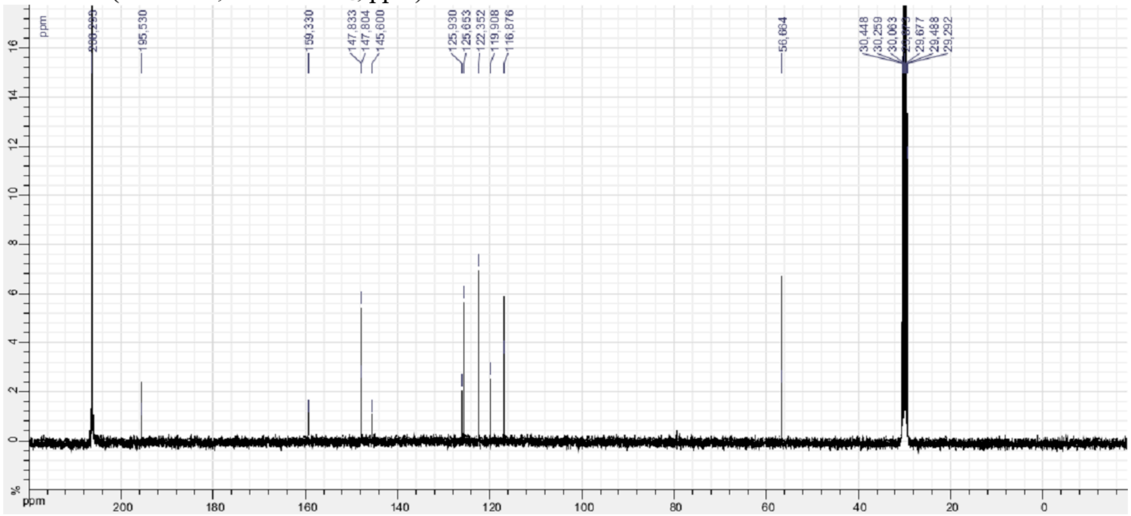

Line#:1 R.Time:14.650(Scan#:1159)
MassPeaks:34
RawMode:Averaged 14.642-14.658(1158-1160) BasePeak:218(1767982)
BG Mode:Calc. from Peak Group 1 - Event 1 Scan

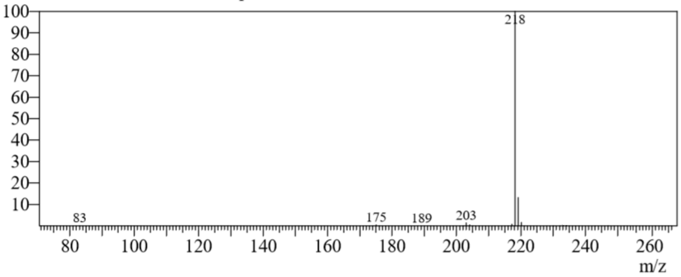

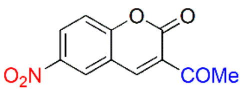

MP-03

3-Acetyl-6-nitro-2H-chromen-2-one ***(MP-03)***

ATR-FTIR

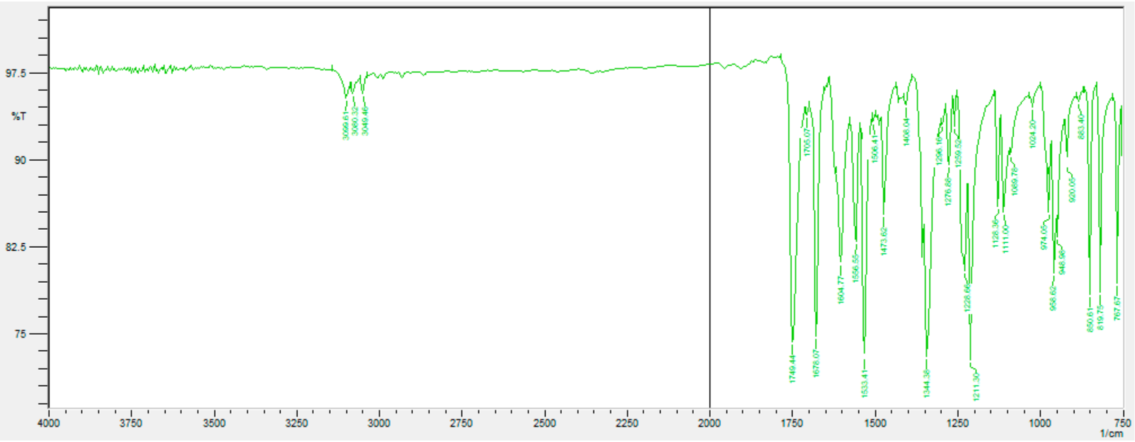

^1H NMR (400 MHz, $CDCl_3$, ppm)

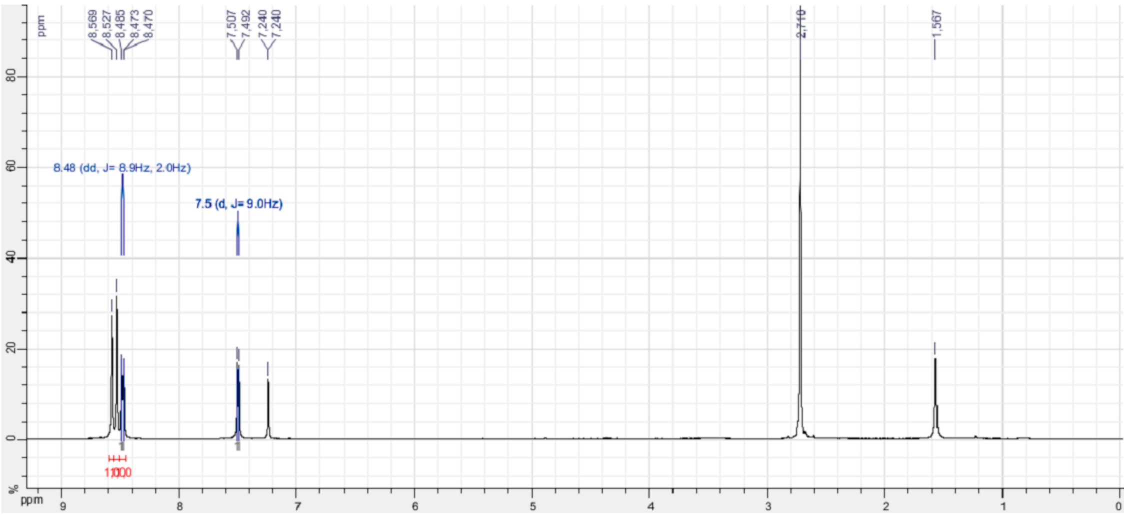

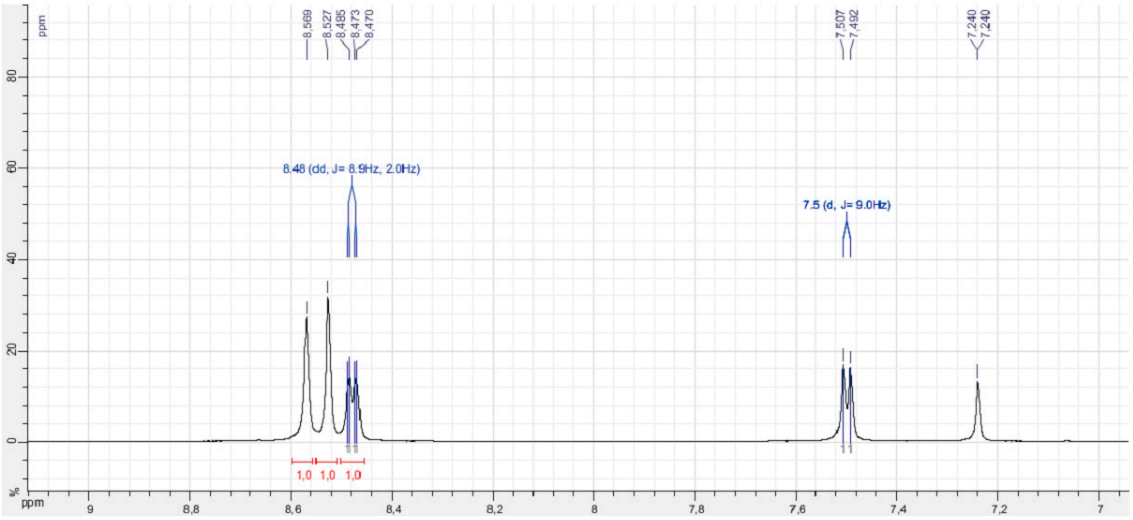

DEPT ^{13}C (100 MHz, $CDCl_3$, ppm)

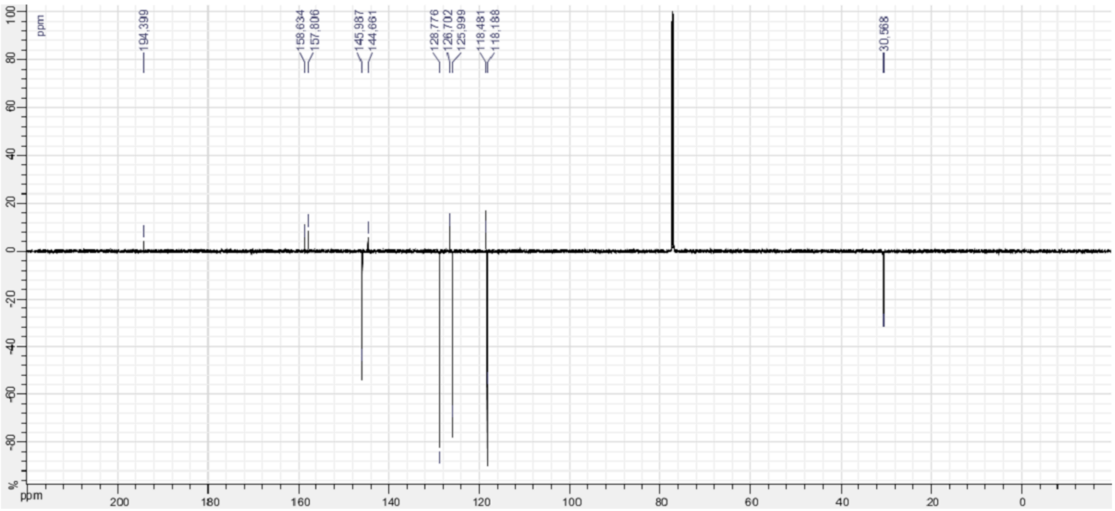

MS

Line#:8 R.Time:18.308(Scan#:1598)
MassPeaks:32
RawMode:Averaged 18.300-18.317(1597-1599) BasePeak:232(8275)
BG Mode:Calc. from Peak Group 1 - Event 1 Scan

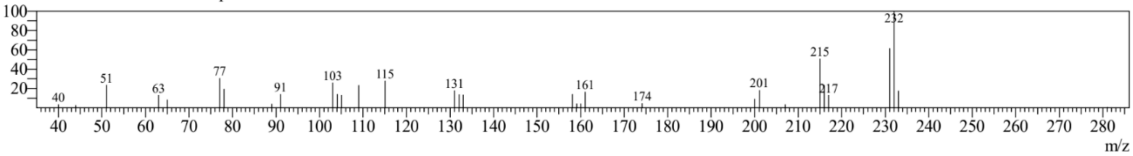

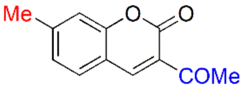

MP-04

3-Acetyl-7-methyl-2H-chromen-2-one ***(MP-04)***

ATR-FTIR

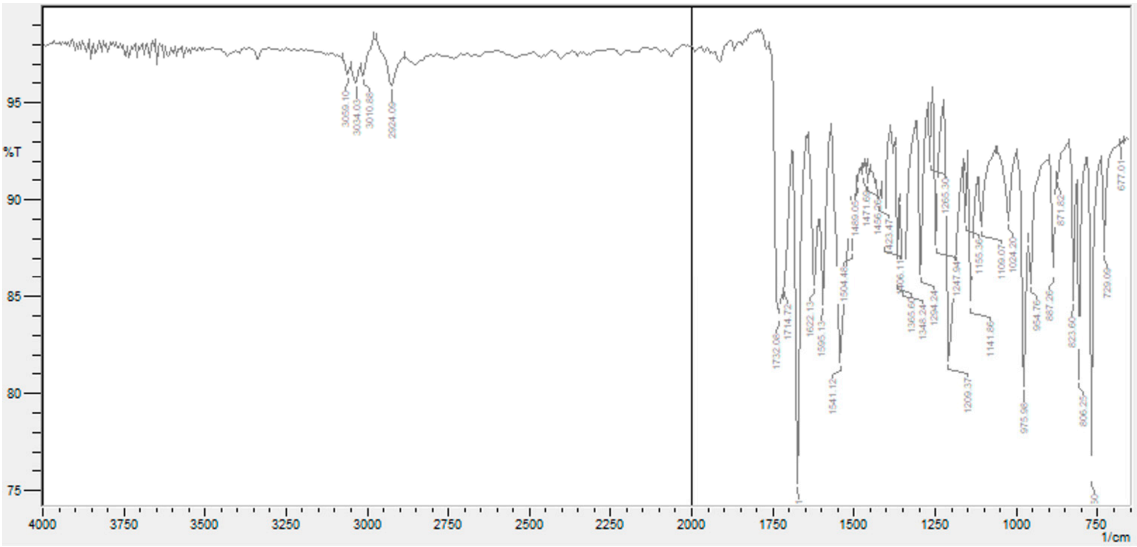

^{1}H NMR (400 MHz, $CDCl_3$, ppm)

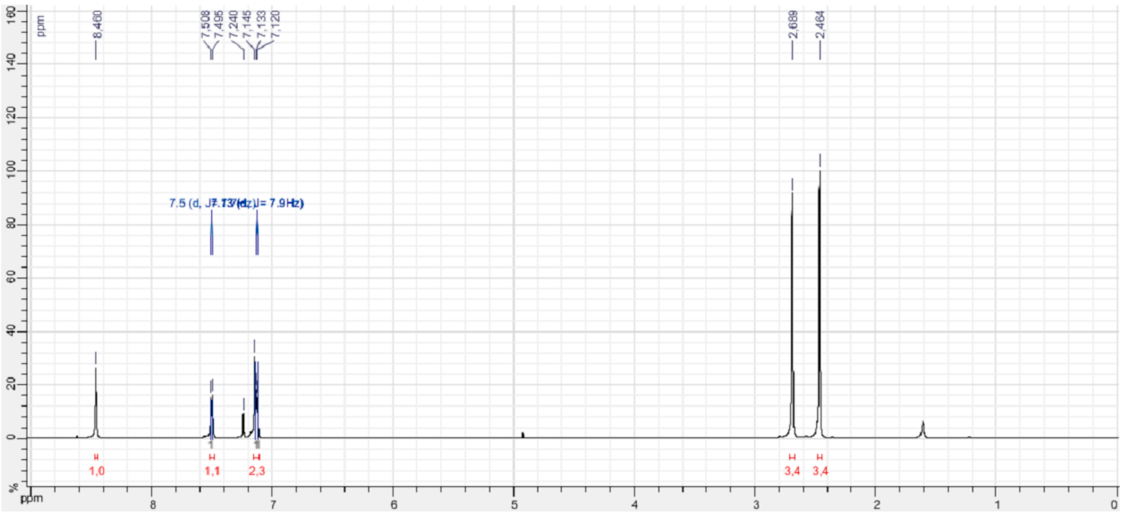

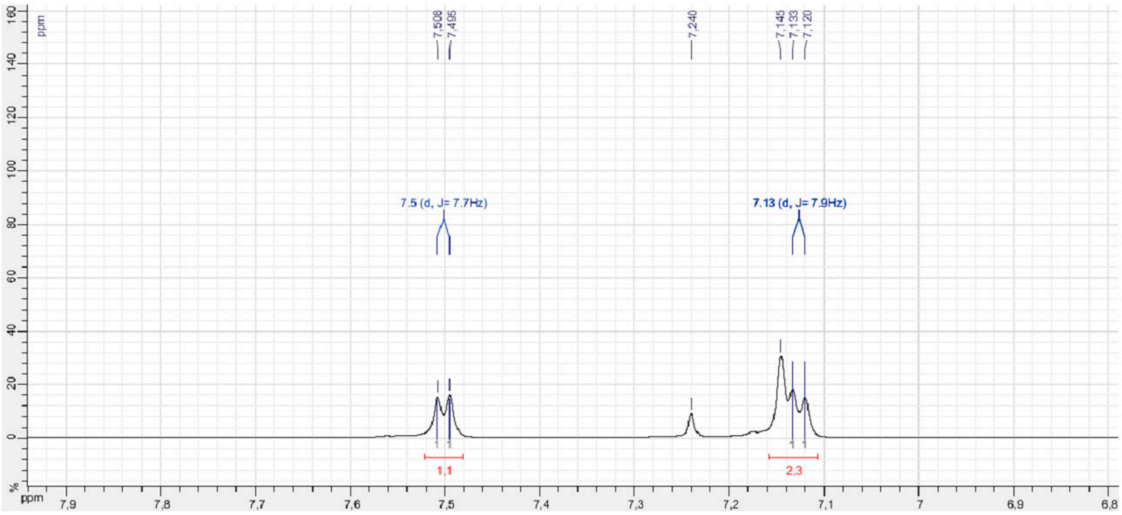

DEPT ^{13}C (100 MHz, $CDCl_3$, ppm)

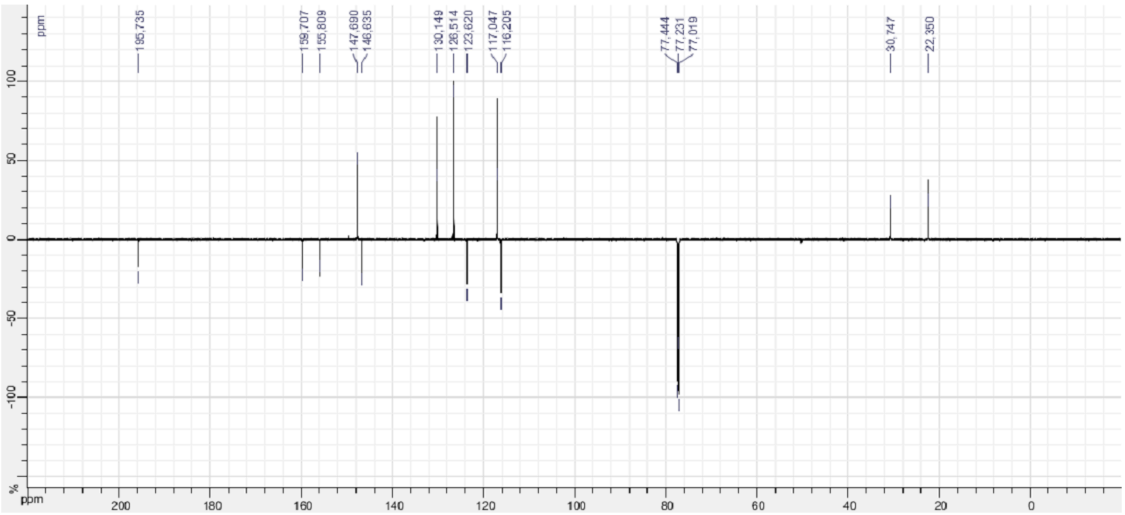

MS

Line#:3 R.Time:13.483(Scan#:1019)
MassPeaks:115
RawMode:Averaged 13.475-13.492(1018-1020) BasePeak:187(7050568)
BG Mode:Calc. from Peak Group 1 - Event 1 Scan

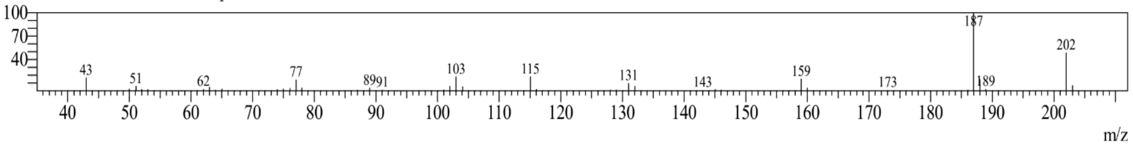

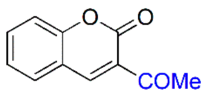

MP-05

3-Acetyl-2H-chromen-2-one ***(MP-05)***

ATR-FTIR

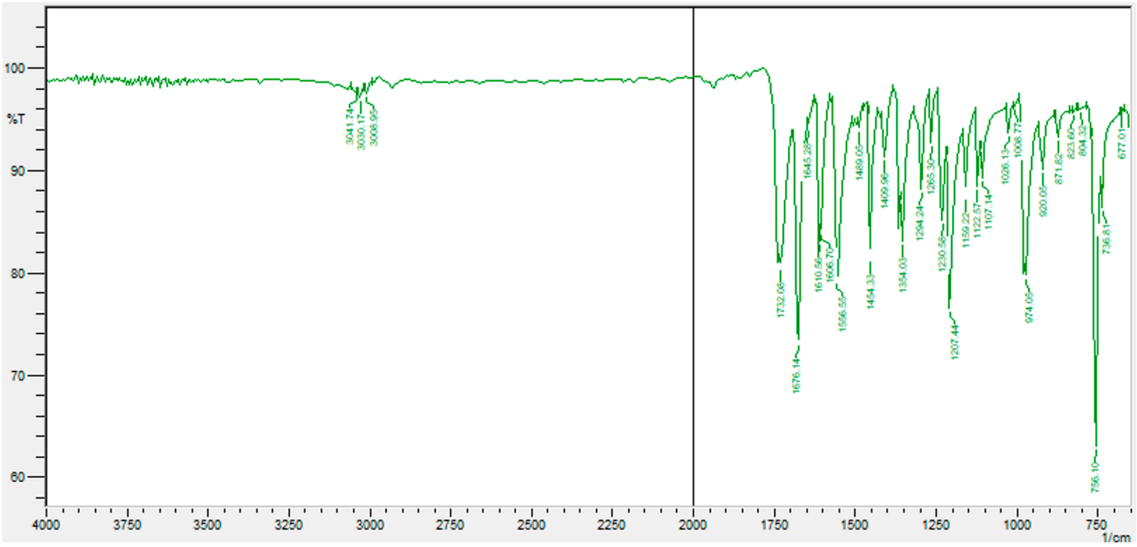

^{1}H NMR (400 MHz, $CDCl_3$, ppm)

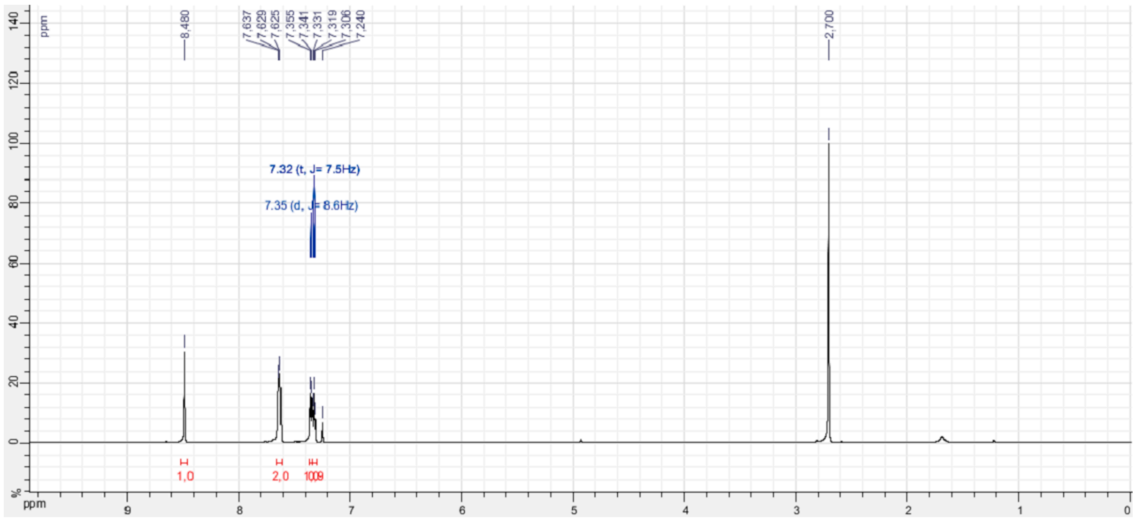

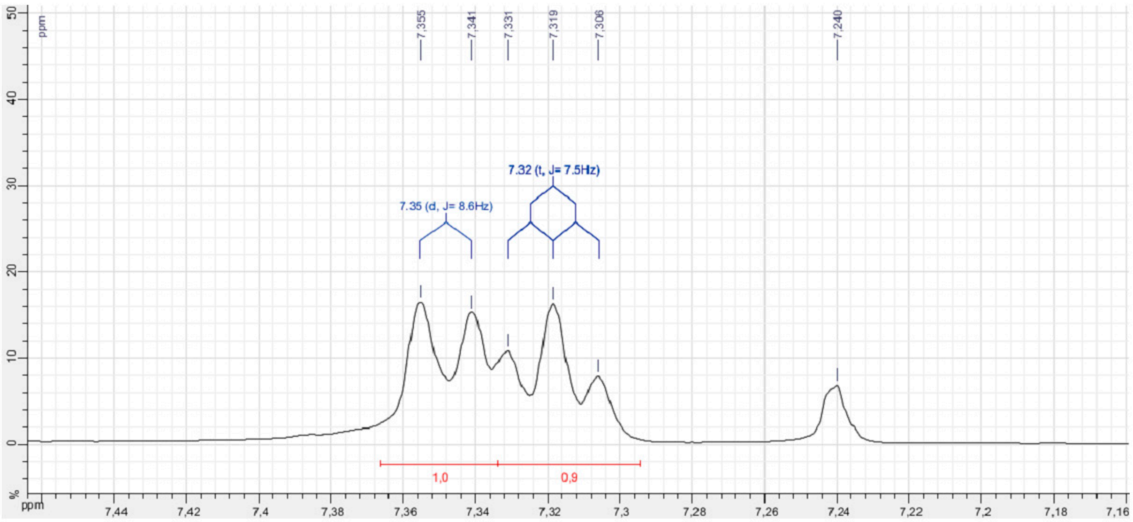

DEPT ^{13}C (100 MHz, $CDCl_3$, ppm)

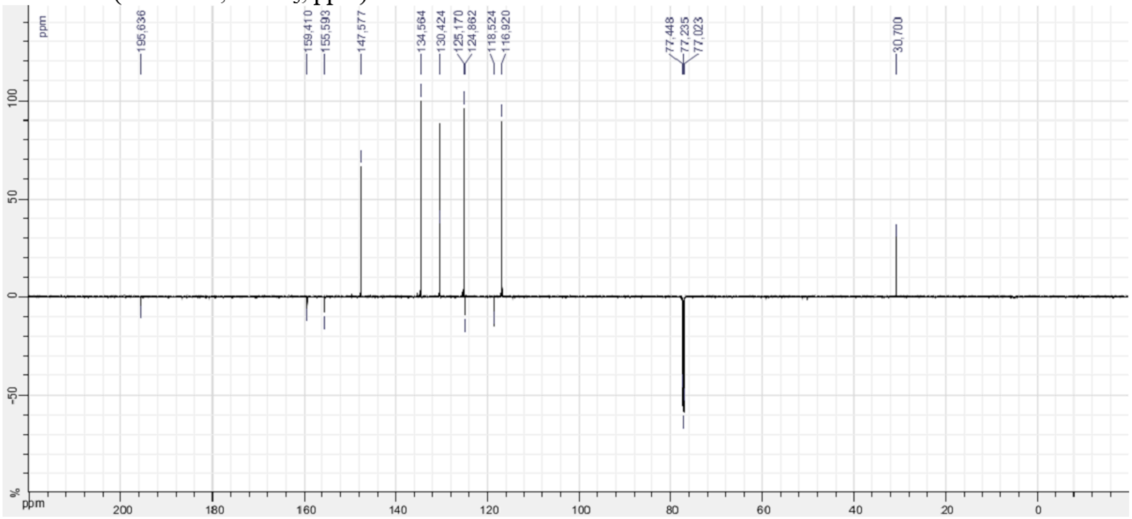

MS

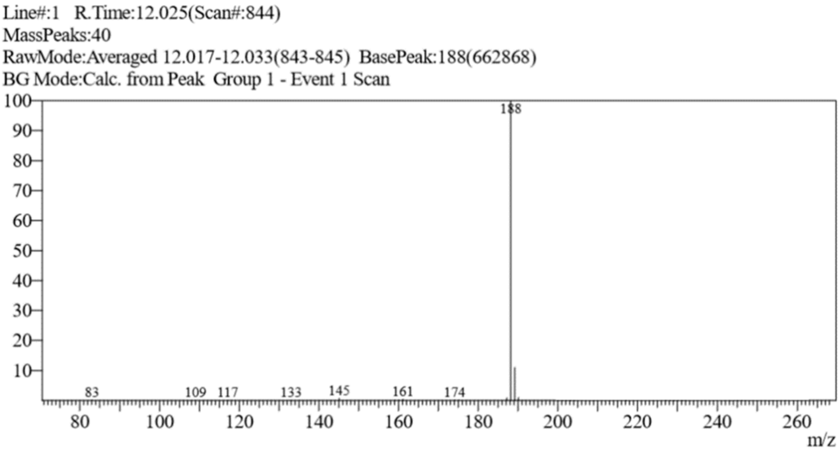

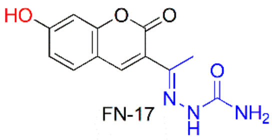

(1E)-1-(1-(7-Hydroxy-2-oxo-2H-chromen-3-yl)ethylidene)semicarbazide ***(FN-17)***

ATR-FTIR

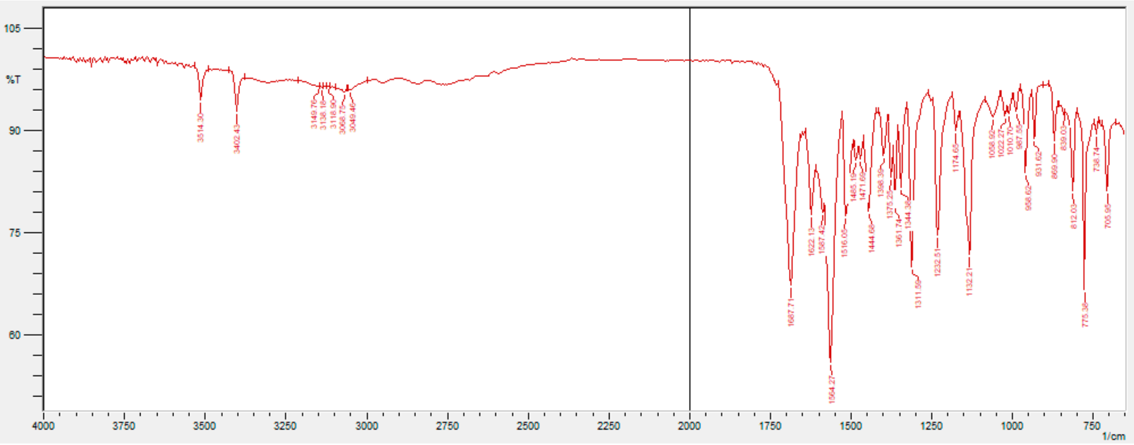

^{1}H NMR (400 MHz, DMSO-d_6, ppm)

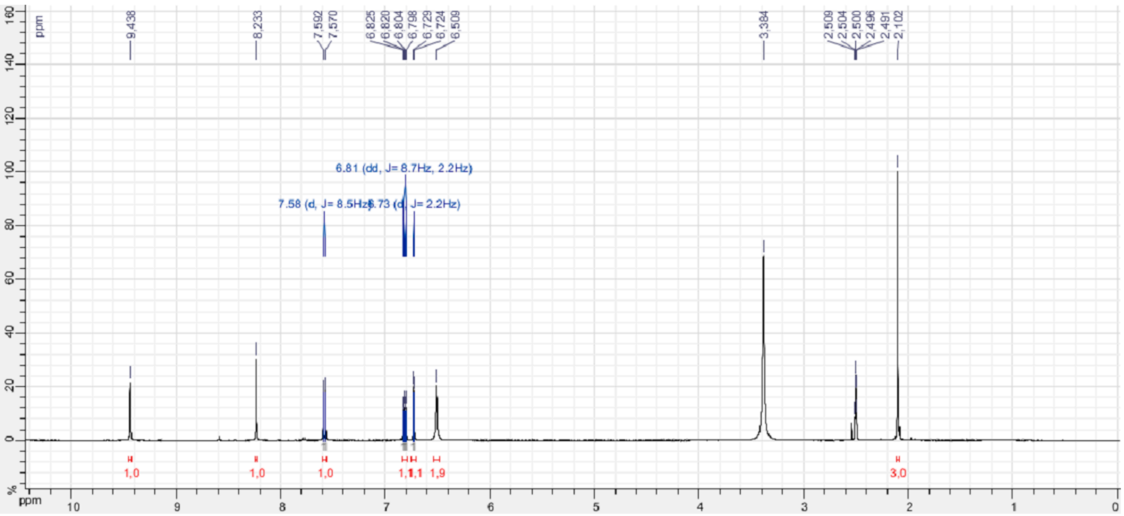

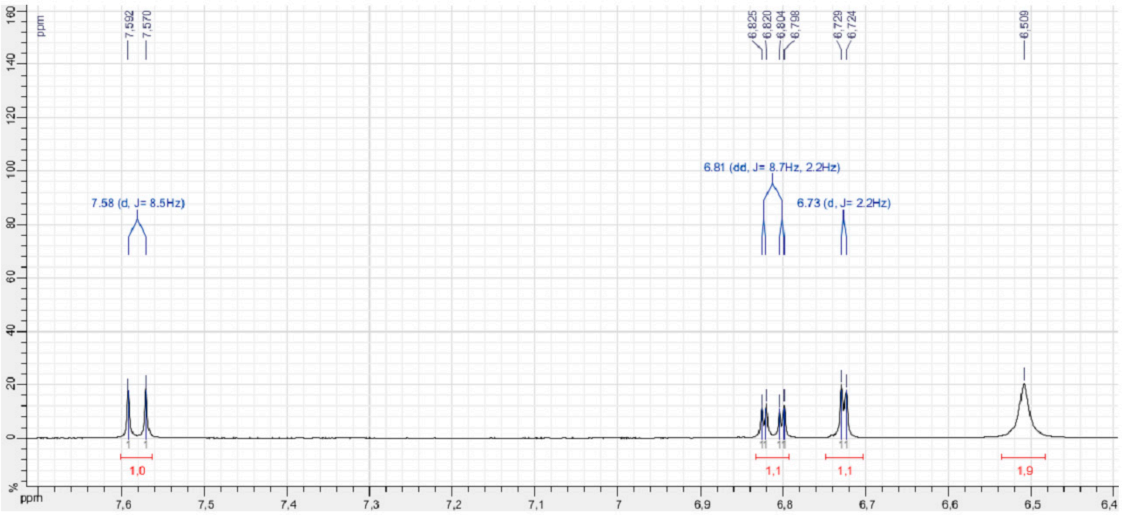

^{13}C NMR (100 MHz, DMSO-d_6, ppm)

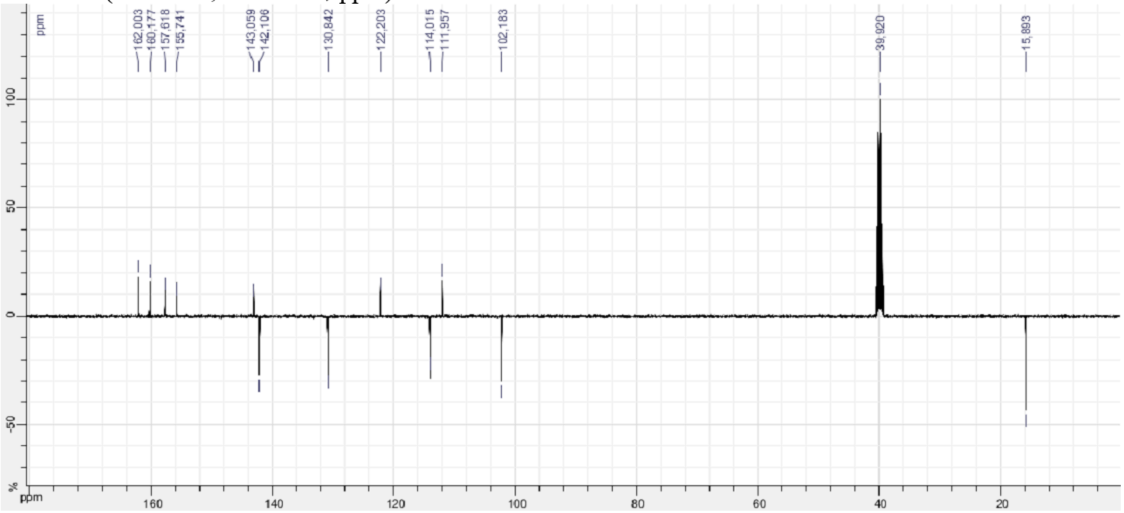

MS

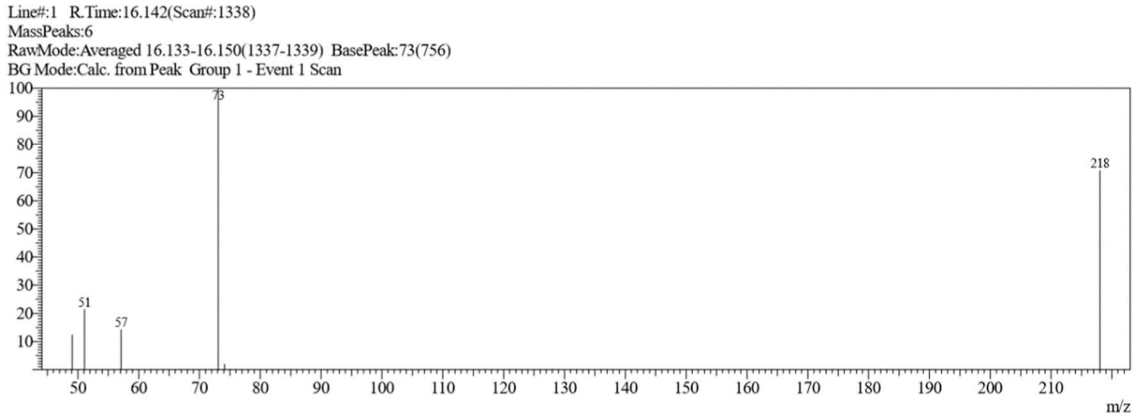

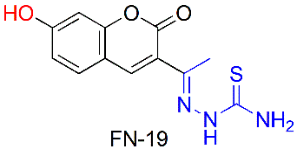

(1E)-1-(1-(7-Hydroxy-2-oxo-2H-chromen-3-yl)ethylidene)thiosemicarbazide ***(FN-19)***

ATR-FTIR

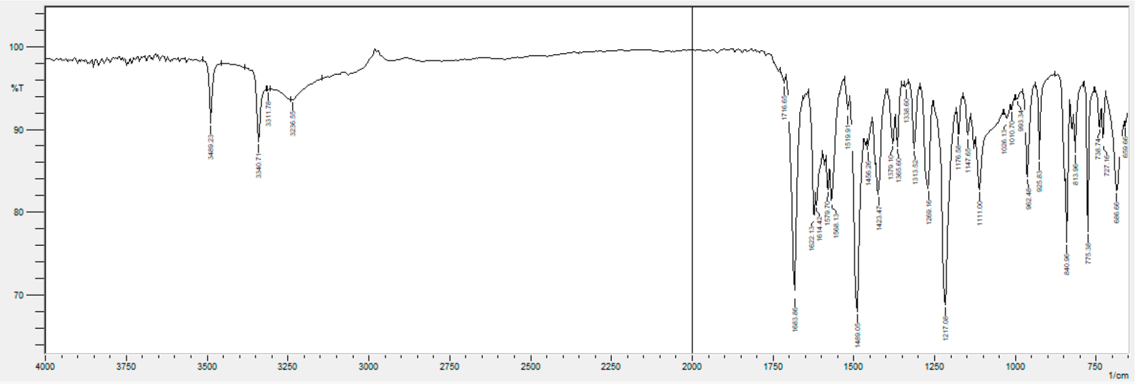

¹H NMR (400 MHz, DMSO-d_6, ppm)

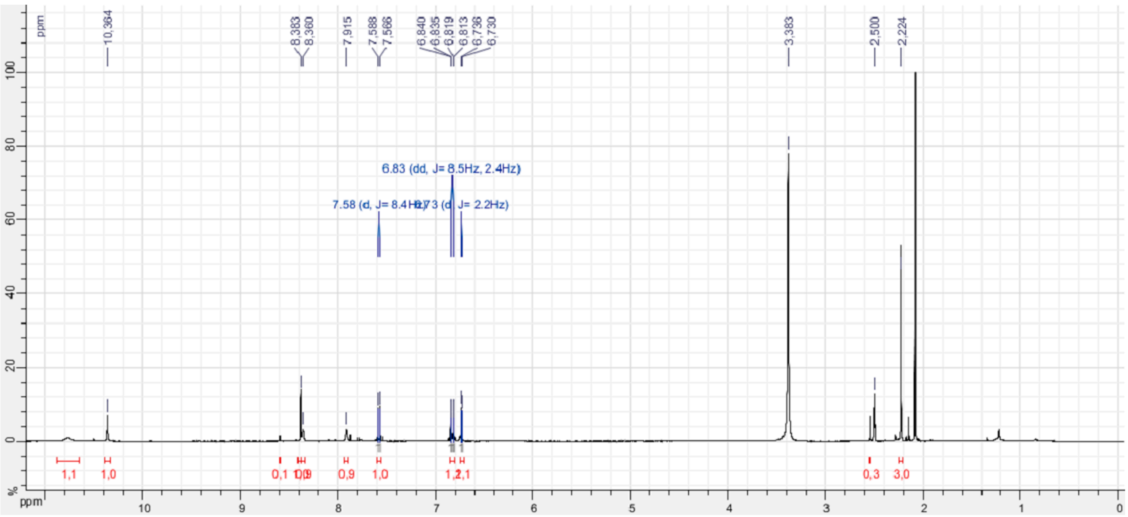

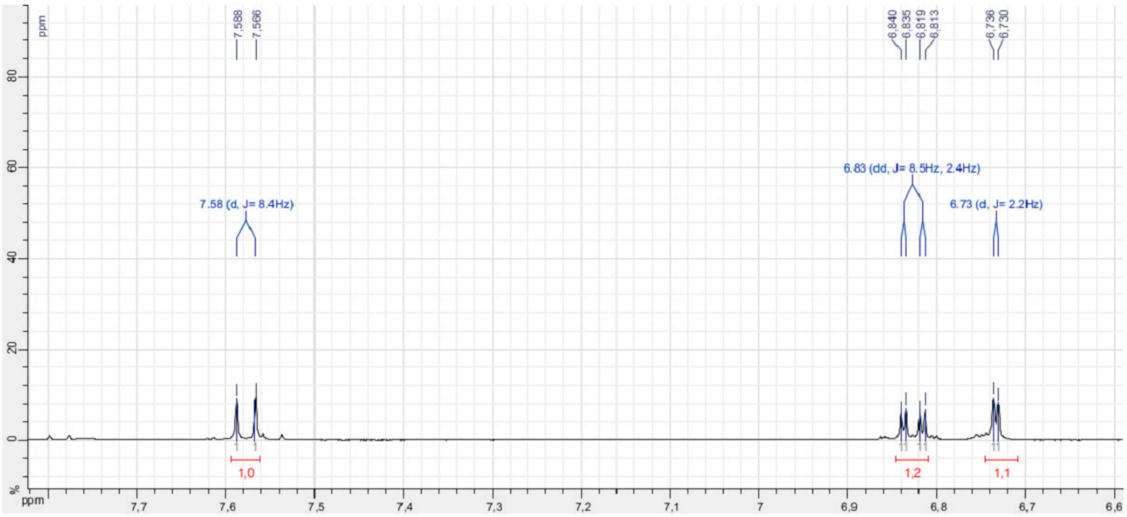

^{13}C NMR (100 MHz, DMSO-d_6, ppm)

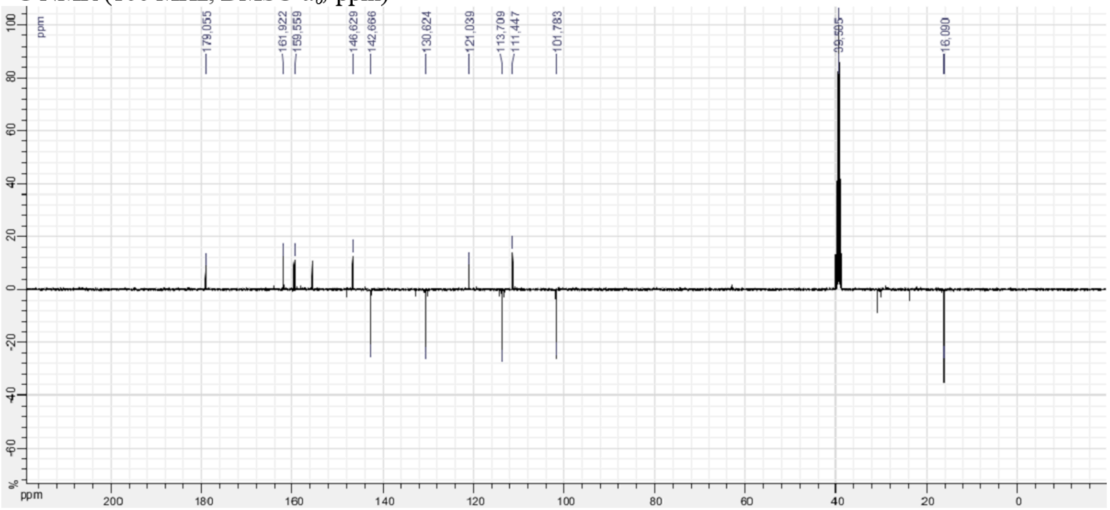

MS

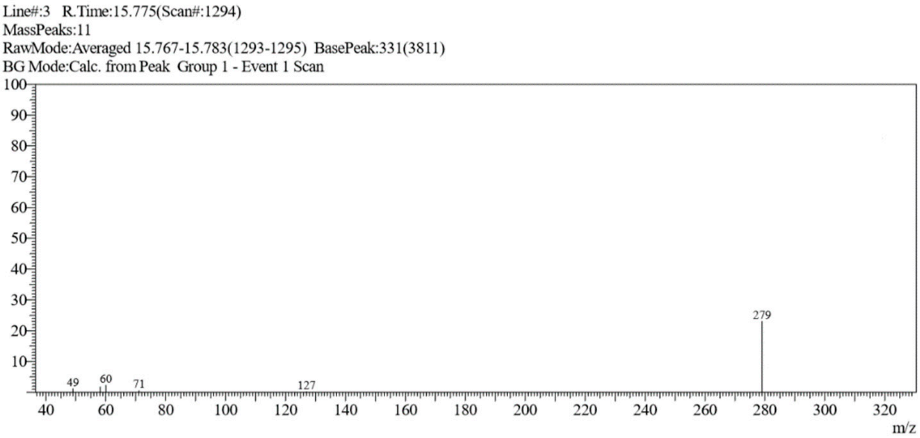

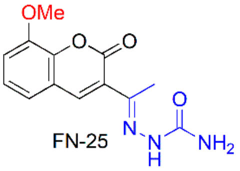

FN-25

(1E)-1-(1-(8-Methoxy-2-oxo-2H-chromen-3-yl)ethylidene)semicarbazide ***(FN-25)***

ART-FTIR

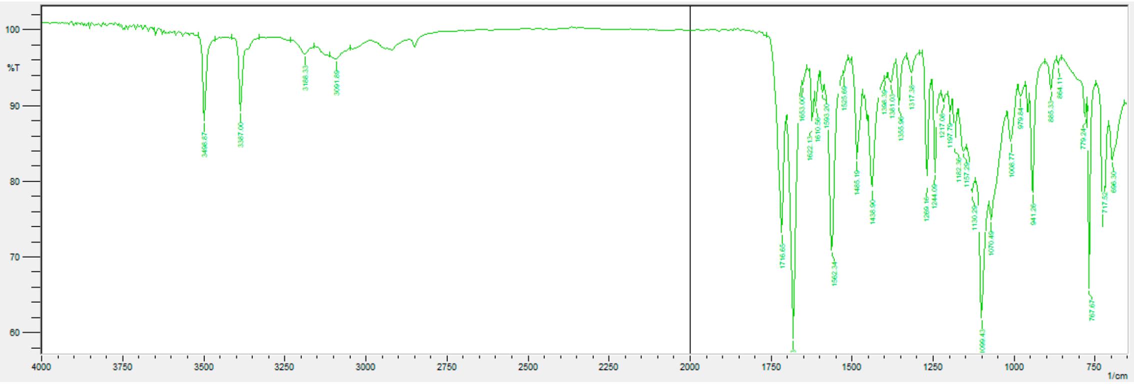

^{1}H NMR (400 MHz, DMSO-d_6, ppm)

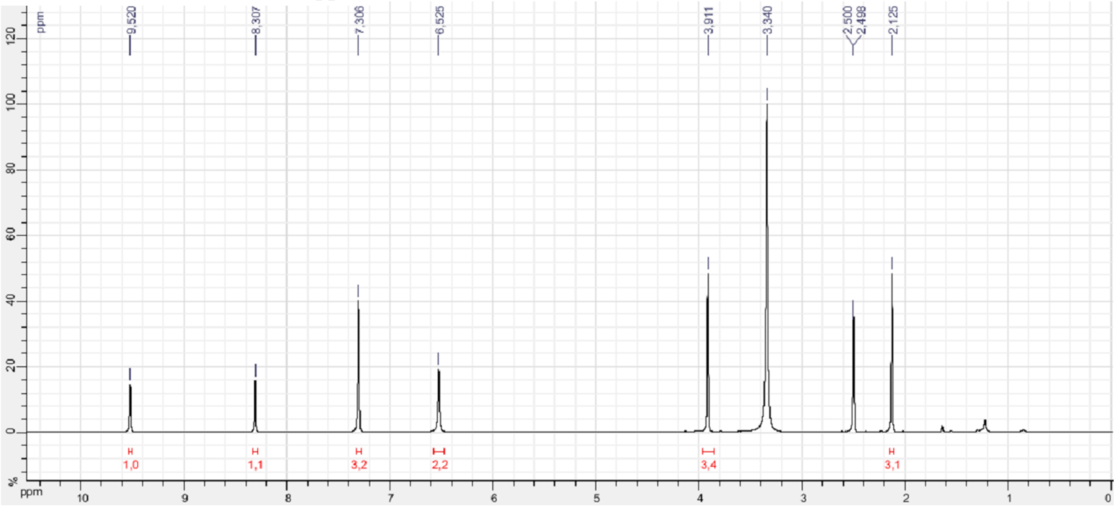

DEPT ^{13}C (100 MHz, DMSO-d_6, ppm)

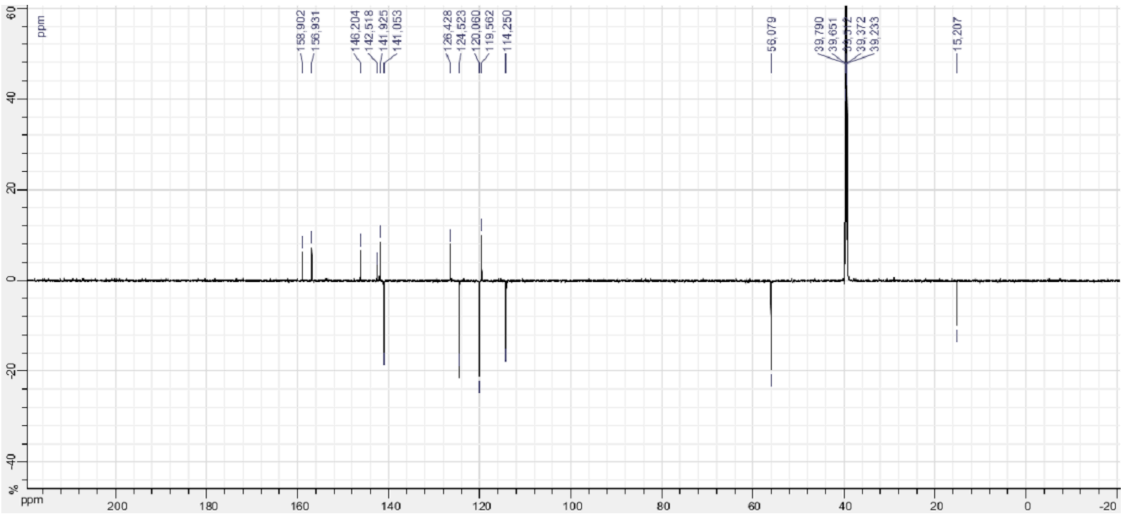

MS

Line#:1 R.Time:17.833(Scan#:1541)
MassPeaks:7
RawMode:Averaged 17.825-17.842(1540-1542) BasePeak:232(19654)
BG Mode:Calc. from Peak Group 1 - Event 1 Scan

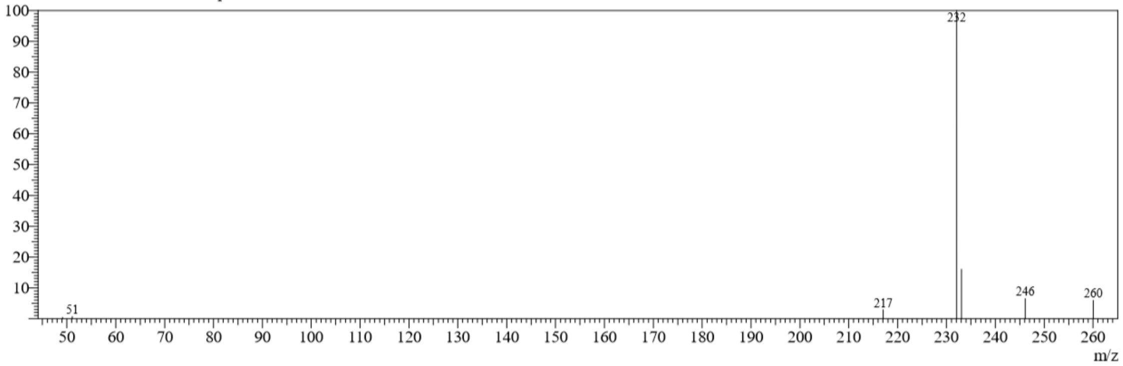

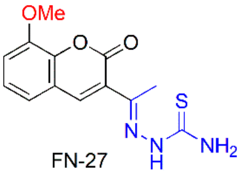

(1E)-1-(1-(8-Methoxy-2-oxo-2H-chromen-3-yl)ethylidene)thiosemicarbazide ***(FN-27)***

ATR-FTIR

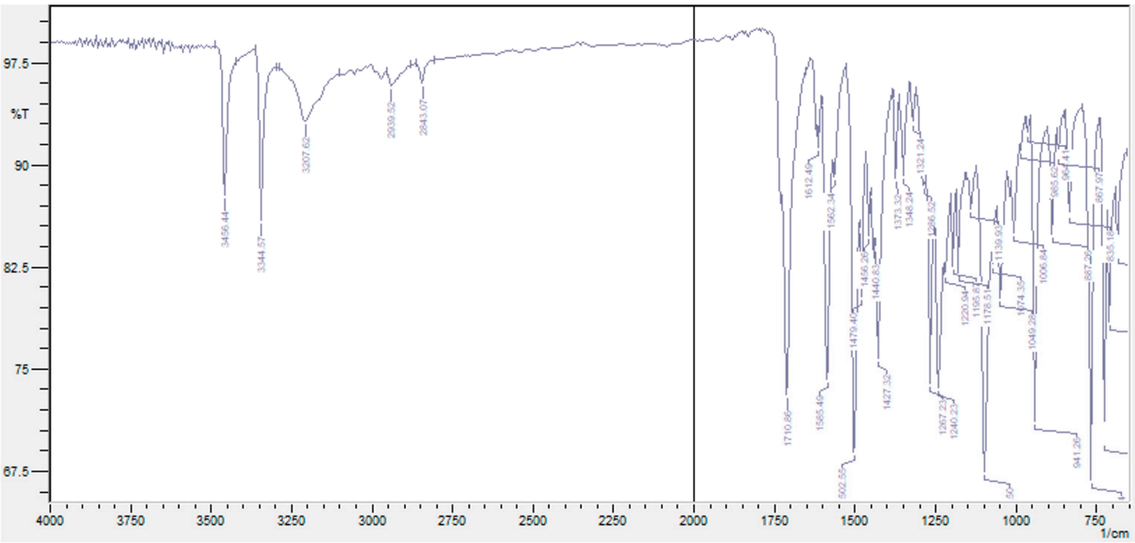

¹H NMR (400 MHz, DMSO-d_6, ppm)

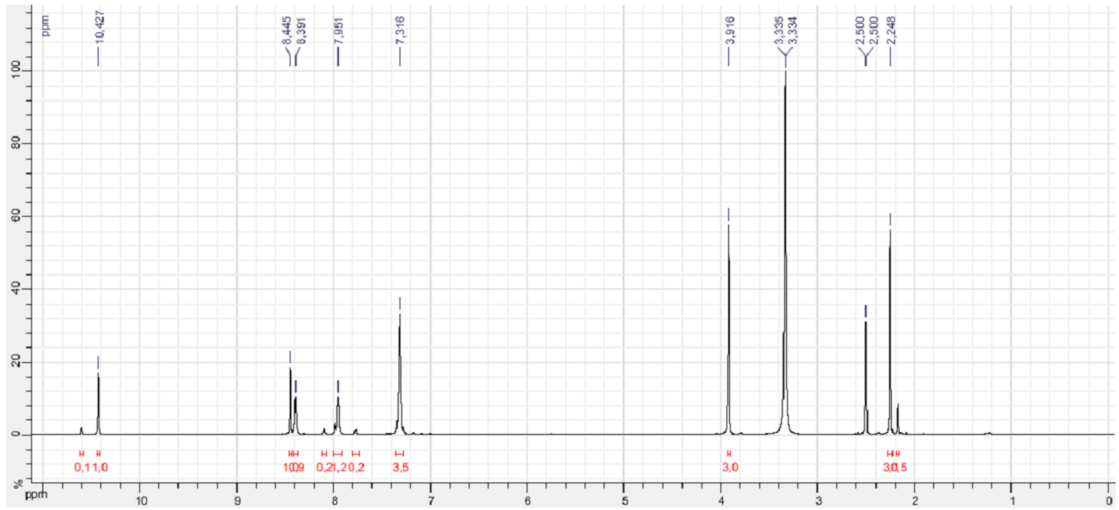

^{13}C NMR (100 MHz, DMSO-d_6, ppm)

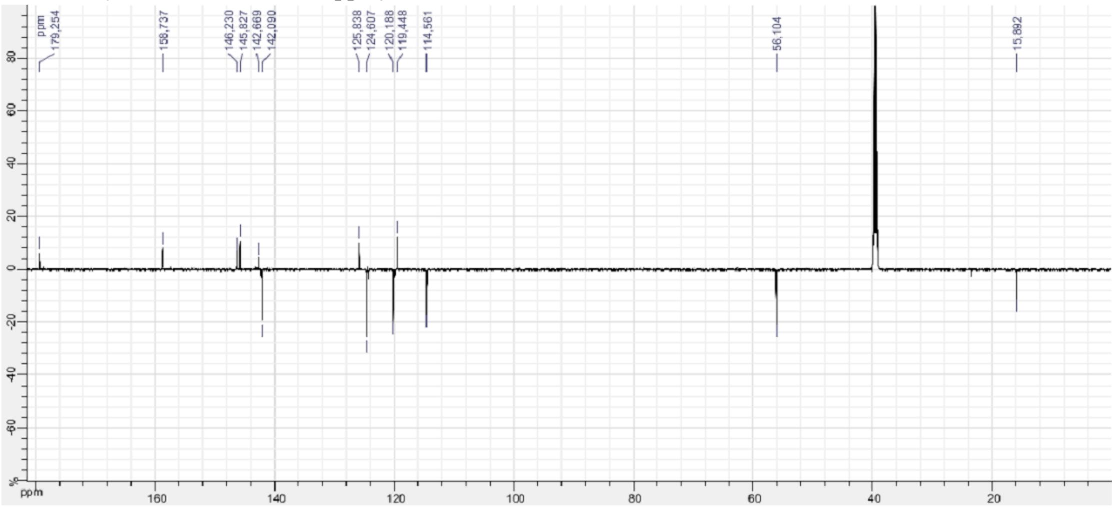

MS

Line#:1 R.Time:18.308(Scan#:1598)
MassPeaks:177
RawMode:Averaged 18.300-18.317(1597-1599) BasePeak:232(312707)
BG Mode:Calc. from Peak Group 1 - Event 1 Scan

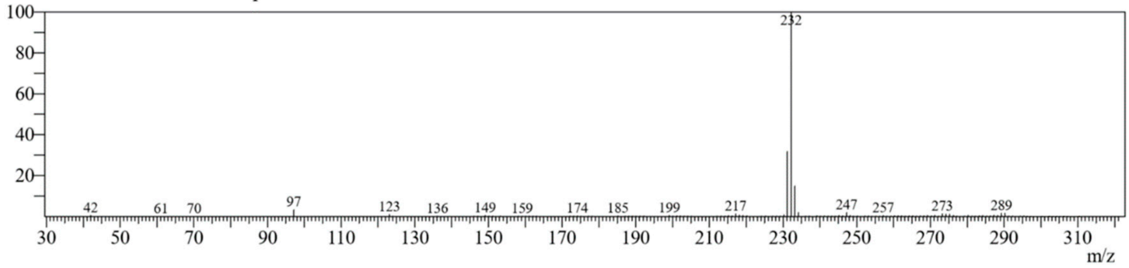

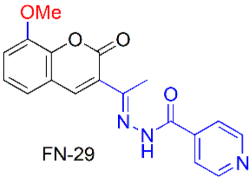

(13E)-N'-(1-(8-Methoxy-2-oxo-2H-chromen-3-yl)ethylidene)isonicotinohydrazide ***(FN-29)***

ATR-FTIR

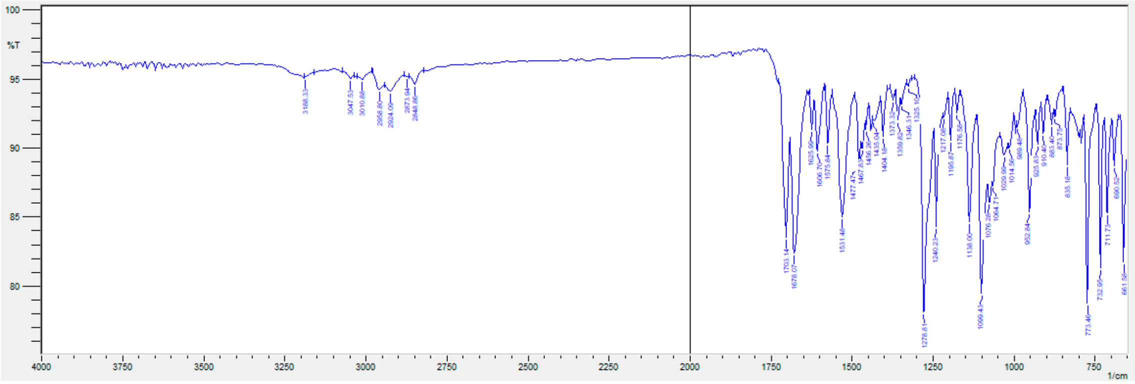

MS

Line#:8 R.Time:18.308(Scan#:1598)
MassPeaks:32
RawMode:Averaged 18.300-18.317(1597-1599) BasePeak:232(8275)
BG Mode:Calc. from Peak Group 1 - Event 1 Scan

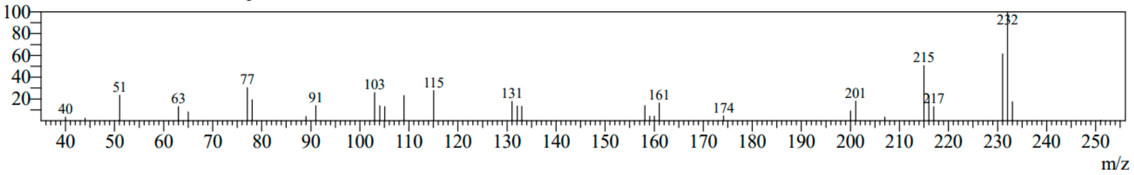

RMSD plot for free-tyrosinase and FN19-tyrosinase complex generated by GROMACS v. 2018.3 within a 100 ns molecular dynamics simulation.

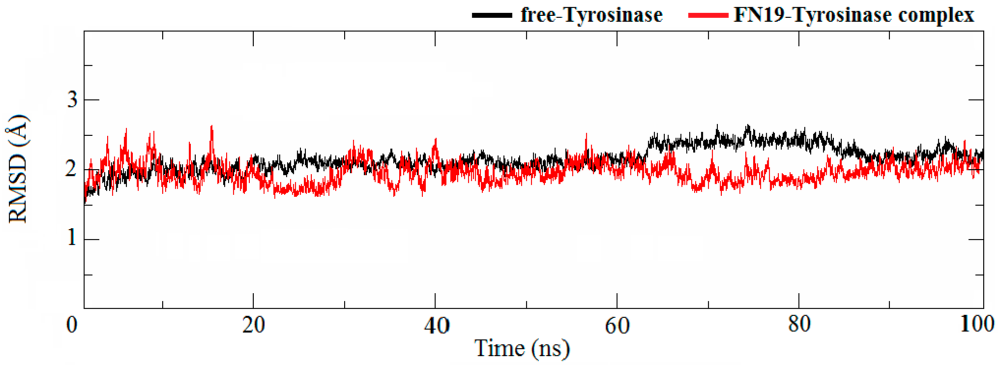

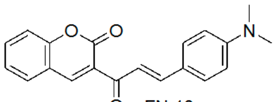

FN-10

3-((E)-3-(4-(Dimethylamino)phenyl)acryloyl)-2H-chromen-2-one ***(FN-10)***

ATR-FTIR

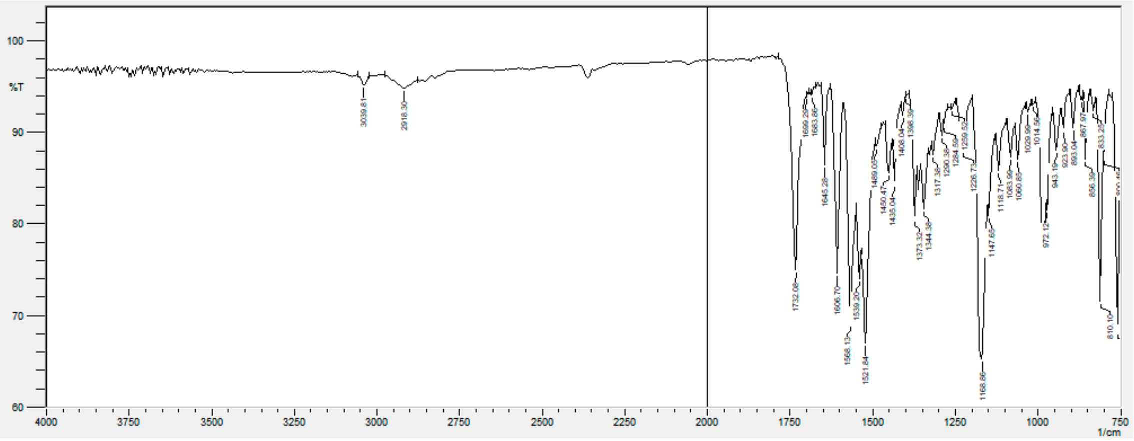

MS

Line#:1 R.Time:29.367(Scan#:2925)
MassPeaks:20
RawMode:Averaged 29.358-29.375(2924-2926) BasePeak:319(22824)
BG Mode:Calc. from Peak Group 1 - Event 1 Scan

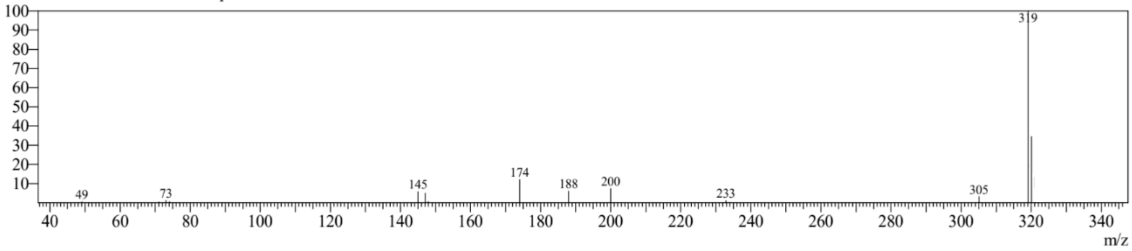

Supplement: Supplementary file 1 [file ijms-24-05216-s001.zip › ijms-2230511-supplementary.pdf]
